# Systematic Review of Instruments Assessing Psychosocial Adaptation and Outcomes Among Families of Children With Congenital Heart Disease

**DOI:** 10.1093/jpepsy/jsad015

**Published:** 2023-05-23

**Authors:** Karen J Eagleson, Desiree McCombs, Tiffany M Gerlich, Robert N Justo, Nadine A Kasparian, Samudragupta Bora

**Affiliations:** Mater Research Institute, Faculty of Medicine, The University of Queensland, Australia; Queensland Paediatric Cardiac Service, Queensland Children’s Hospital, Australia; Mater Research Institute, Faculty of Medicine, The University of Queensland, Australia; Mater Research Institute, Faculty of Medicine, The University of Queensland, Australia; School of Psychology, Faculty of Health and Behavioural Sciences, The University of Queensland, Australia; Queensland Paediatric Cardiac Service, Queensland Children’s Hospital, Australia; Cincinnati Children’s Center for Heart Disease and Mental Health, Heart Institute and Division of Behavioral Medicine and Clinical Psychology, United States of America; Department of Pediatrics, University of Cincinnati College of Medicine, United States of America; Mater Research Institute, Faculty of Medicine, The University of Queensland, Australia; School of Psychology, Faculty of Health and Behavioural Sciences, The University of Queensland, Australia; Department of Pediatrics, University Hospitals Rainbow Babies & Children's Hospital, Case Western Reserve University School of Medicine, United States of America

**Keywords:** congenital heart disease, family, high-risk infant, neurodevelopment, psychological functioning, quality of life

## Abstract

**Objective:**

This systematic review identified instruments quantitatively assessing psychosocial adaptation and outcomes in families of children with congenital heart disease (CHD) and evaluated instrument psychometrics.

**Methods:**

Following Preferred Reporting Items for Systematic Reviews and Meta-Analyses guidelines and a prospectively registered protocol, electronic databases (CINAHL, Embase, PubMed/MEDLINE, PsycINFO, and SCOPUS) were searched from inception until June 20, 2021 for peer-reviewed articles published in English, reporting quantitative data on psychosocial outcomes among parents/caregivers, siblings, or family system. Instrument characteristics and psychometrics were extracted, and adapted COnsensus-based Standards for the selection of health Measurement INstruments (COSMIN) criteria were applied to assess instrument quality. Descriptive statistics and narrative synthesis were used for analysis.

**Results:**

Overall, 108 articles reporting on 107 distinct samples across 26 countries met inclusion. Across those articles, 40 instruments assessed psychological functioning or distress, 12 assessed coping, 11 assessed quality of life constructs, 10 assessed parenting stress/caregiver burden, 10 assessed family functioning/impact, 10 assessed stress appraisal, 5 assessed sibling psychosocial outcomes, and 2 assessed couple relationship satisfaction/strain. Applying COSMIN criteria to available data on original instrument development articles/manuals for English language instruments (*n* = 54), 67% scored a positive property evidence rating for content validity, 39% for internal consistency, 4% for test–retest reliability, and 9% for responsiveness (longitudinal validity).

**Conclusions:**

Studies vary widely in instruments used to assess psychosocial adaptation and outcomes among families of children with CHD. Instrument selection informed by robust key psychometrics, increased psychometric reporting, development of both a “toolkit” approach and a comprehensive CHD-specific family instrument are among key recommendations.

## Introduction

There is increasing recognition of the impacts of congenital heart disease (CHD) on families. Parents of children with CHD are at elevated risk for psychological distress, lower quality of life, and challenges to coping and overall family functioning (Gregory et al., 2018; Jackson et al., 2015; Wei et al., 2015). There is, however, wide variability in reported outcomes across studies (Gregory et al., 2018; Jackson et al., 2015; Soulvie et al., 2012; Wei et al., 2015; Woolf-King et al., 2017). Rates of parental psychological distress, for example, are reported as higher (Lawoko & Soares, 2002; Wray et al., 2018), similar (Fischer et al., 2012), or lower (Spijkerboer et al., 2007; Visconti et al., 2002) than population-based norms or the general community. Findings on consequences of CHD on family life and functioning are also varied (Sood et al., 2021), with greater financial, social, and daily living challenges reported in some studies (Connor et al., 2010; Gregory et al., 2018; Werner et al., 2014; Wray & Maynard, 2005), and increased cohesiveness of the family unit and strengthening of familial relationships in others (Wei et al., 2015; Wray et al., 2018). While there is growing evidence of parental experiences within the CHD context (Sood et al., 2021; Tesson et al., 2019; Woolf-King et al., 2017), there is a paucity of data on couple relationship satisfaction as well as the experiences of siblings, with variability in outcomes also evident. Levels of relationship satisfaction, both lower and comparable to typical pregnant samples, 1 month after prenatal CHD diagnosis have been reported (Lisanti et al., 2021a; Rychik et al., 2013). Recently published reviews of the impact of CHD on siblings (Parker et al., 2020; Schamong et al., 2022) identify impaired psychosocial wellbeing in 14–40% of siblings across studies, as well as inconclusive evidence regarding the role of disease severity as a factor impacting sibling wellbeing.

Variability in reported psychosocial outcomes among family members may be attributed, at least to some extent, to factors such as CHD complexity and severity (Mörelius et al., 2002; Wei et al., 2015; Wray et al., 2018), the timing of psychosocial assessment across the medical trajectory (Jantien Vrijmoet-Wiersma et al., 2009; Landolt et al., 2011), sociodemographic factors such as education, occupation, country of origin (Franck et al., 2010; Landolt et al., 2011; Spijkerboer et al., 2007; Yildiz et al., 2009), and familial role (Gregory et al., 2018; Wray et al., 2018). In studies involving siblings, data are often collected via parent-proxy reports (than via direct sibling self-report), highlighting the potential risks of report source bias (Senner & Fish, 2012; Van Roy et al., 2010).

Another potential source of variability is associated with the measurement of the constructs (Soulvie et al., 2012; Wei et al., 2015; Woolf-King et al., 2017). A “lack of consensus on how and what to measure” (Wei et al., 2015), and wide variation and inconsistency in instrument use may contribute to variance in results across studies (Bratt & Moons, 2015; Garcia Rodrigues et al., 2022; Gregory et al., 2018). Thus, a better understanding of instruments used to assess psychosocial outcomes, including their psychometric properties, is important to appreciate the true impact of CHD on families as reported in existing studies and to improve instrument selection in future research to enable study comparison and meta-analysis of outcomes data, generalizability of findings, and evaluation of interventions (Sood et al., 2021; Tesson et al., 2019; Wei et al., 2015).

Prioritization of the psychological needs of families of children with CHD, inclusive of routine screening as part of usual congenital cardiac care, is also needed (Woolf-King et al., 2017, Kasparian et al., 2016, Kasparian & Kovacs, 2022). Psychosocial assessment standards and guidelines have been established in clinical populations such as pediatric oncology (Kazak et al., 2015) and the perinatal period (Austin and Highet, 2017) including routine and systematic assessment recommendations informed by systematic reviews of relevant instruments. Similarly, psychosocial care consensus recommendations (Utens et al., 2018) and more recently, the American Heart Association scientific statement of psychological outcomes and interventions for individuals with CHD have been published (Kovacs et al., 2022), with Standards of Care for Childhood-onset Heart Disease (Sholler & Selbie, 2022) currently in development in Australia. Nonetheless, to date, no systematic review of instruments to assess the experience and outcomes of families with a child with CHD has been undertaken.

Accordingly, the aims of this systematic review were twofold. First, to identify and critically appraise instruments used to quantitatively assess psychosocial adaptation and outcomes among family members (parents, caregivers, and siblings) of children with CHD and within the family system. Second, to evaluate instrument characteristics and psychometric properties, including using the COnsensus-based Standards for the selection of health Measurement Instrument (COSMIN) criteria for good measurement properties (Prinsen et al., 2016; Terwee et al., 2007) for content validity, internal consistency, test–retest reliability, and responsiveness (i.e., longitudinal validity). These data will be synthesized to provide key recommendations for future research and clinical practice.

## Methods

### Data Sources and Search Strategies

This systematic review was conducted in accordance with the Preferred Reporting Items for Systematic Reviews and Meta-Analyses guidelines (Page et al., 2021), and the study protocol was prospectively registered via PROSPERO (CRD42019128611). Before protocol development, the scope of the work was conceptualized through a comprehensive review of relevant CHD literature and conceptual frameworks (Lawoko, 2007; Lisanti, 2018; Mussatto, 2006). The search strategy was developed in conjunction with an academic librarian, using MeSH terms, keywords, and relevant database syntax adaption with Boolean operators. The full list of search terms and search strings for various databases are provided in Supplementary Material 1.

Original, peer-reviewed journal articles were eligible for inclusion if published in the English language and reported on quantitative data on psychosocial adaptation and outcomes for parents, caregivers, siblings, and/or the family unit in the context of CHD. No age limit was set regarding the child with CHD in the family. While inclusion of studies with >50% sample of families of children with CHD was initially planned, the decision was made to exclude studies with any non-CHD samples, thereby including only instruments specifically chosen to examine families of children with CHD. No limitations were set in terms of study design.

A comprehensive search of five electronic databases (CINAHL, Embase, PubMed/MEDLINE, PsycINFO, and SCOPUS) was undertaken by two independent reviewers (KE and DM) from database inception on 16 February 2019 and updated on 20 June 2021 (KE and SB). Citations were exported to EndNoteX8 referencing software and after duplicate removal, the remaining articles were screened first by title and abstract and then by full text, with any discrepancies resolved through discussion among reviewers. Additional hand searching of the reference lists of key existing review articles (Gregory et al., 2018; Jackson et al., 2015; Kasparian et al., 2019; Parker et al., 2020; Schamong et al., 2022; Soulvie et al., 2012; Wei et al., 2015; Woolf-King et al., 2017) and reference lists of final articles for additional publications for inclusion was also undertaken. Where duplicate or overlapping samples were identified, studies with the maximum number of relevant instruments or the largest sample were retained.

### Data Extraction and Analysis

Forms were collaboratively developed by the first and senior/last authors and amended based on pilot data extraction. Data extraction was undertaken by the first author and verified for accuracy by the senior/last author (CHD study characteristics) and a research assistant (instrument characteristics and psychometrics), with all discrepancies resolved by discussion with reviewers.

Data captured included (a) CHD study characteristics (author, publication year, country, relevant constructs and instruments, response rate, child with CHD characteristics, and parent/family participant characteristics, and conceptual framework), (ii) instrument characteristics (author, publication year, type, constructs assessed, domains/scales, item number, response scale, timeframe of response, administration method, completion time, scoring, licensing/cost, and languages available), and (iii) psychometric properties (original instrument development–sample, internal consistency, test–retest reliability, construct, and criterion validity; CHD study–internal consistency).

Identified instruments were categorized as assessing “psychological functioning and distress,” “parenting stress and caregiver burden,” “family impact or functioning,” “coping,” “health-related-/quality of life,” “stress appraisal,” “couple relationship satisfaction or strain,” and “sibling psychosocial outcomes.” Categorization was an iterative process informed by the relevant literature and conceptual frameworks, the purpose of the instrument as identified by original development and included CHD articles, and research team expertise. Construct definitions are further described in the relevant results section below. Instruments assessing constructs in more than one category were included in all relevant categories. Instrument characteristics and psychometric properties were extracted predominantly from primary sources of instrument development, that is, peer-reviewed articles and technical manuals. Additionally, instrument publisher/distributor websites were searched for instrument characteristic data. To analyze the data, both descriptive statistics (count and percentage) and narrative synthesis were used.

An adapted version of the COSMIN criteria for good measurement properties (Prinsen et al., 2016; Terwee et al., 2007) was used to evaluate the psychometric characteristics of identified instruments. These criteria for appraisal of good measurement properties were established to enable meaningful comparison of instruments in systematic reviews (Terwee et al., 2007) and to guide instrument selection in the development of core outcome sets (Prinsen et al., 2016). Of the nine measurement properties identified as most relevant through an expert Delphi study, we included four with the highest percentage (≥90%) of international consensus, and that include the most important measurement properties, that is, content validity and internal structure (Prinsen et al., 2016). COSMIN criteria were applied to the original articles of instrument development and instrument technical manuals, where accessible. A consensus approach by the first and senior/last authors was used. This assessment was restricted to validated instruments and those in the English language because of the linguistic competency of the study team.

### Quality Assessment

The methodological quality of included CHD studies was assessed independently by the first and senior/last authors using adapted Newcastle Ottawa Scale (Wells et al., 2019) for cohort and cross-sectional studies to ensure homogeneity of assessment across different study designs. Scores indicated a good (≥7), fair (5–6), or poor (4) quality rating. Where appropriate, the National Institutes of Health quality assessment tool for randomized and nonrandomized controlled interventions was also used, providing good, fair, and poor ratings.

## Results

The study search process is illustrated in Supplementary Figure 2. Methodological characteristics of studies are presented in Supplementary Table 3. Of the 108 final articles, three-quarters (*n* = 79; 73%) were published since 2010. The final articles represented 107 distinct samples across 26 countries, with most studies conducted in the USA (*n* = 37) and Australia (*n* = 12), and eight studies undertaken in low/low- to middle-income countries. Samples predominantly included families of children with heterogeneous CHD types (*n* = 94). Twenty-eight studies reported solely on outcomes for families of infants (aged ≤12 months) and 54 studies included children aged ≥5 years. Of the 106 studies with parent/caregiver participants, 27 focused solely on mothers, 58 reported participation of fathers/male caregivers, and 21 studies did not report participant sex or gender. No studies focused exclusively on fathers’ experiences. Eight studies reporting sibling psychosocial outcomes using full instruments and subscales were identified. Just one-quarter of studies (*n* = 29) identified the use of a conceptual/theoretical framework to inform study protocol. For the quality of included studies, 52 were considered good, 38 were fair, and 18 were poor quality.

Overall, 97 instruments, including long, short, and modified versions, assessing psychosocial adaptation and outcomes were identified. Almost all instruments (95/97) captured self-report data, with one clinician observation instrument (Campbell et al., 1992) and one instrument capturing parental perception of the effect of caregiving on sibling wellbeing (Breslau et al., 1981). Subscales of three parent/caregiver instruments capturing their reporting of sibling experiences were also identified (Azhar et al., 2016; Pai et al., 2008; Stein & Riessman, 1980). Instruments were predominantly generic (62/97), with illness-focused (20/97), study-specific (7/97) instruments, and one CHD-specific instrument also identified (Jackson et al., 2017). Six identified instruments were adaptable to a specific context (Carver, 1997; Carver et al., 1989; Foa et al., 1997; Folkman & Lazarus, 1985; Lisanti et al., 2021a; Svavarsdottir & McCubbin, 1996; Werner et al., 2019; Weiss & Charles, 1997). The frequency of instrument usage is shown in Supplementary Figure 4. In total, COSMIN criteria for good measurement properties were applied to 54 instruments and were not applied where instruments were non-English (*n* = 24), study-specific/not validated (*n* = 11), inaccessible (*n* = 7), or a clinician-observed (*n* = 1) instrument.

Instrument usage, characteristics, and psychometric data are reported below by construct and in order of frequency of use across studies. Instrument characteristics and psychometric properties including CHD study internal consistency data, are presented as Supplementary Table 5 and 6, respectively, with COSMIN results in Table I. Measurement property and criteria rating definitions are detailed in Table II.

**Table I.** COnsensus-based Standards for the Selection of Health Measurement INstruments (COSMIN) Criteria for Good Measurement Properties Results

Instrument	Content Validity	Internal Consistency	Reliability	Responsiveness
Psychological distress and functioning				
Brief Symptom Inventory (BSI)	0	+	?	0
General Health Questionnaire (GHQ)	0	?	0	0
Perceived Stress Scale (PSS-10)	0	+	0	0
General Stress Item	?	?	0	0
Index of Clinical Stress	0	–	0	0
Depression Anxiety Stress Scales (DASS)	+	+	0	0
Depression Anxiety Stress Scales (DASS-21)	?	?	0	0
Hospital Anxiety and Depression Scale (HADS)	0	?	0	0
Beck Depression Inventory (BDI-2)	+	+	?	0
Center for Epidemiologic Studies Depression Scale (CES-D)	+	+	?	+
Patient Health Questionnaire (PHQ)	+	?	?	–
Edinburgh Postnatal Depression Scale (EPDS)	+	?	0	0
State-Trait Anxiety Inventory (STAI)	+	+	?	0
Generalized Anxiety Disorder-7 (GAD-7)	+	+	+	0
Self-Rating Anxiety Scale (SAS)	+	0	0	0
Visual Analogue Scale (Anxiety)	+	0	?	–
Taylor Manifest Anxiety Scale (TMAS)	–	0	?	–
Impact of Event Scale—Revised (IES-R)	?	+	?	0
PTSD Checklist—Civilian Version (PCL-C)	0	+	?	?
Posttraumatic Diagnostic Scale (PDS)	0	?	+	0
Maslach’s Burnout Inventory (MBI)	+	+	?	0
Acute Stress Disorder Scale (ASDS)	0	+	?	0
Maternal Worry Scale (MWS)	+	+	?	0
Beck Hopelessness Scale (BHS)	+	+	0	+
Life Orientation Test (LOT)	+	+	?	0
Personal Feelings Questionnaire-2 (PFQ-2)	?	?	?	0
Parenting stress and caregiver burden				
Parenting Stress Index-4 (PSI-4)	+	+	?	0
Parenting Stress Index-Short Form-4 (PSI-SF-4)	+	+	?	0
Pediatric Inventory for Parents (PIP)	+	?	0	0
Quality of life and health-related quality of life				
36-Item Short-Form Health Survey (SF-36)	+	+	?	–
Pediatric Quality of Life (PedsQL) Family Impact Module^a^	+	?	0	0
Perceived Quality of Life Scale (PQOL)	+	0	0	0
World Health Organization Quality of Life (WHOQOL-100)	+	+	0	0
World Health Organization Quality of Life BREF (WHOQOL-BREF)	+	?	?	0
Family Member Well-being Index (FMWB)	0	?	0	0
Family impact or functioning				
Psychosocial Assessment Tool (PAT 2.0)	+	–	?	0
Family Assessment Device (FAD)	?	?	0	0
Impact on Family Scale (IOFS)	+	?	0	0
Impact on Family Scale Revised (IOFS-R)	+	+	0	0
Family Inventory of Life Events and Changes (FILE)	+	–	?	0
Pediatric Quality of Life (PedsQL) Family Impact Module^a^	+	?	0	0
Family Hardiness Index (FHI)	0	–	?	0
Coping				
Coping Health Inventory for Parents (CHIP)	+	?	0	0
COPE Inventory	+	–	?	0
Brief COPE	+	–	0	0
Ways of Coping Questionnaire (WCQ)	+	?	0	0
Response to Stress Questionnaire (RSQ-CHD)^a^	+	?	0	0
Stress appraisal				
Hassles and Uplifts Scale Combined (HSUP)	+	0	0	0
Life Experiences Survey (LES)	?	0	?	+
Social Readjustment Rating Scale (SRRS)	?	0	0	0
Parent Stressor Scale: Infant Hospitalization (PSS:IH)	+	?	?	0
Parent Stressor Scale: Neonatal Intensive Care Unit (PSS:NICU)	+	?	0	0
Neonatal Unit Parental Stressor Scale (NUPS)	+	+	0	+
Response to Stress Questionnaire (RSQ-CHD)^a^	+	?	0	0
Couple relationship satisfaction or strain				
Dyadic Adjustment Scale (DAS)	+	+	0	0
Sibling psychosocial outcomes				
Sibling Perception Questionnaire (SPQ)	+	?	0	+

a Reported in two categories.

**Table II.** Adapted COnsensus-based Standards for the Selection of Health Measurement INstrument (COSMIN) Criteria for Good Measurement Properties (Prinsen et al., 2016; Terwee et al., 2007)

Measurement Property	Definition	Rating	Criteria
Content validity	The extent to which the domain of interest is comprehensively sampled by the items in the instrument	+	A clear description is provided in the measurement aims, and the item selection AND target population and (investigators OR experts) were involved in item selection
		?	A clear description of above-mentioned aspects is lacking OR only target populations involved OR doubtful design or method
		–	No target population involvement
		0	No information found on target population involvement
Internal consistency	The extent to which items in a (sub)scale are intercorrelated, thereby measuring the same construct	+	Factor analysis performed on adequate sample size (7 × No. of items and ≥100) AND Cronbach’s alpha(s) calculated per dimension AND Cronbach’s alpha(s) between .70 and .95
		?	No factor analysis OR Cronbach’s alpha(s) OR doubtful design or method
		–	Cronbach’s alpha(s) <.70 or >.95, despite adequate design and method
		0	No information found on internal consistency
Test–retest reliability	The degree to which the measurement is free from measurement error	+	Intraclass Correlation Coefficient or weighted Kappa ≥.70
		?	Intraclass Correlation Coefficient or weighted Kappa not reported
		–	Criteria for + not met
		0	No information found on reliability
Responsiveness	The ability of a measurement instrument to detect change over time in the construct to be measured	+	At least 75% of the results are in accordance with the hypotheses
		?	No correlations with changes in instrument(s) measuring related construct(s) AND no differences between changes in relevant groups reported
		–	Criteria for + not met
		0	No information found on responsiveness

### Psychological Functioning and Distress (40 Instruments)

Thirty-nine self-report instruments were used across 61 (57%) distinct samples (Alkan et al., 2017; Awaad & Darahim, 2015; Berant et al., 2001; Bevilacqua et al., 2013; Bishop et al., 2019; Blue et al., 2015; Bratt et al., 2019; Brosig et al., 2007b; Callahan et al., 2019; Campbell et al., 1995; 1992; Cantwell-Bartl & Tibballs, 2017; Chien et al., 2021; Cohn, 1996; Coşkuntürk and Gözen, 2018; Davis et al., 1998; Denniss et al., 2019; Diffin et al., 2016; Doherty et al., 2009; Dulfer et al., 2015; Fischer et al., 2012; Franich-Ray et al., 2013; Gaskin et al., 2021; Guan et al., 2014; Hancock et al., 2018; Helfricht et al., 2008; Hoehn et al., 2004; Hunt et al., 2020; Jackson et al., 2020; Jordan et al., 2014; Kiliçarskan-Törüner et al., 2012; Kumar et al., 2019; Landolt et al., 2011; Lawoko & Soares, 2006; Li et al., 2018; Lisanti et al., 2017, 2021a,b; López et al., 2016; McCusker et al., 2010; 2012; McKechnie et al., 2016; Medoff-Cooper et al., 2020; Menahem et al., 2008; Miller et al., 2021; Mussatto et al., 2021; Pinto et al., 2016; [Bibr jsad015-B155][Bibr jsad015-B155]; Re et al., 2018; Roberts et al., 2021; Rona et al., 1998; Rychik et al., 2013; Spijkerboer et al., 2007; Tallon et al, 2015; Uhm et al., 2019; Utens et al., 2000; Uzger et al. 2015; van der Mheen et al., 2019; Jantien Vrijmoet-Wiersma et al., 2009; Werner et al., 2019; Yildiz et al., 2009; Zhang et al., 2021) to assess various aspects of psychological functioning and distress, including symptoms of anxiety, depression, and traumatic stress (Beck et al., 1996; Bryant et al., 2000; Cox et al., 1987; Derogatis & Melisaratos, 1983; Derogatis, 2001; Derogatis et al., 1976; El Missiry et al., 2003; Foa et al., 1997; Fontanesi et al., 1985; Goldberg & Blackwell, 1970; Hornblow & Kidson, 1976; Kroenke & Spitzer, 2002; Lovibond & Lovibond, 1995; Spielberger et al., 1983; Spitzer et al., 2006; Taylor, 1953; Ulusoy et al., 1998; Veit & Ware, 1983; Weathers et al., 1993; Weiss & Charles, 1997; Zigmond & Snaith, 1983; Zung, 1971). One clinician-observed instrument of behavior at stress points was also identified (Campbell et al., 1992). The most frequently used instruments were the State-Trait Anxiety Inventory including Dutch, Chinese, Turkish, and Korean translations across 20 studies, and the Beck Depression Inventory and Brief Symptom Inventory each used across five studies. Additional instruments to assess relevant constructs including general stress (Abell, 1991; Cohen & Williamson, 1988; Elo et al., 2003), burnout (Maslach et al., 1996), worry (DeVet & Ireys, 1998; van Rijsoort et al., 1999), hope (Chan et al., 2012) and hopelessness (Beck et al., 1974), optimism (Scheier & Carver, 1985), mood (Cheng, 2011), and guilt and shame (Harder & Zalma, 1990) were also identified, as well as study-specific instruments assessing emotional impacts of having a child with CHD (Blue et al., 2015; Hunt et al., 2020) and reaction to diagnosis (Cohn, 1996).

Applying COSMIN criteria for good measurement properties across 26 instruments, a positive rating was scored for content validity in 13 instruments and internal consistency in 14 instruments, with two scoring a positive rating for test–retest reliability, and two for responsiveness. For the most frequently used instruments, the State-Trait Anxiety Inventory and Beck Depression Inventory scored a positive rating for content validity and internal consistency, with the Brief Symptom Inventory scoring a positive rating for internal consistency. Reliability data were reported for 12 instruments in 18 CHD studies (see Supplementary Table 6); internal consistency was predominantly between .70 and .95, that is, a positive rating (Prinsen et al., 2016; Terwee et al., 2007), with inter-rater reliability reported (*r* = .85) in the one study using a clinician-observed instrument.

### Parenting Stress and Caregiver Burden (10 Instruments)

Ten instruments used to evaluate stress and strain experienced in the parenting role, including in the context of illness were identified across 30 (28%) distinct samples. Seven instruments of parenting stress (Abidin, 2012; Barsella et al., 2021; Haverman et al., 2013; Östberg et al., 1997; Streisand et al., 2001; Jantien Vrijmoet-Wiersma et al., 2010) were identified across 27 distinct samples (Barsella et al., 2021; Bishop et al., 2019; Brosig et al., 2007a; Carey et al., 2002; Caris et al., 2016; Chaisom et al., 2010; Chang et al., 2020; Choi & Lee, 2021; De Stasio et al., 2019; Ezzat et al., 2016; Goldberg et al., 1991; Golfenshtein et al., 2017; Jackson et al. 2020; Kaugars et al., 2018; Lee et al., 2007; Majnemer et al., 2006; Medoff-Cooper et al., 2020; Mörelius et al., 2002; Poh et al., 2020; Mussatto et al., 2021; Re et al., 2018; Sarajuuri et al., 2012; Torowicz et al., 2010; Uzark & Jones, 2003; van der Mheen et al., 2019; Visconti et al., 2002; Jantien Vrijmoet -Wiersma et al., 2009). Most frequently used were the Parenting Stress Index (Abidin, 2012) and the Parenting Stress-Short Form (Abidin, 2012) across 8 and 12 studies, respectively, including Dutch, Korean, Arabic, Chinese, and Thai translations of the Parenting Stress Index-Short Form. The Pediatric Inventory for Parents (Streisand et al., 2001) including a short-form Dutch version was used to assess illness-related parenting stress in eight studies. The use of both a generic and illness-specific instrument for parenting stress was identified in three studies. Three instruments assessing caregiver burden (Lee & Mok, 2011; Svavarsdottir & McCubbin, 1996) including Chinese and Turkish translations of the Zarit Caregiver Burden Interview (Lu et al., 2009; Özer et al., 2012) were identified across three distinct samples (Bektas et al., 2020; Svavarsdottir & McCubbin, 1996; Zhang et al., 2020). COSMIN criteria were applied to three instruments, with positive ratings for content validity scored for all three, and internal consistency of the Parenting Stress Index and Parenting Stress Index-Short Form. Reliability data were reported for five instruments in nine CHD studies (Bishop et al., 2019; Chang et al., 2020; Choi & Lee, 2021; Ezzat et al., 2016; Jackson et al., 2020; Lee et al., 2007; Svavarsdottir & McCubbin, 1996; Torowicz et al., 2010; Jantien Vrijmoet-Wiersma et al., 2009 (*α* = .73–.96).

### Quality of Life and Health-Related Quality of Life (11 Instruments)

Parental/caregiver quality of life or health-related quality of life, broadly conceptualized as the individual’s perception of their physical and mental wellbeing and function, including in the context of illness, was assessed across 21 distinct samples (22 samples; 21% inclusive of additional constructs) (Alkan et al. 2017; Azhar et al., 2016; Bektas et al., 2020; Bevilacqua et al., 2013; Denniss et al., 2019; Eagleson et al., 2013; Edraki et al., 2014; Goldbeck & Melches, 2006; Hancock et al., 2018; Hussein & Authman, 2013; Kaugars et al., 2018; Landolt et al., 2011; Lee et al., 2020; Levert et al., 2017; Medoff Cooper et al., 2020; Mussatto et al., 2021; Simeone et al., 2018; Stoffel et al., 2017; van der Mheen et al., 2019; Warnakulasooriya & Kasturiaratchi, 2020; Zhang et al., 2021). Eight multidimensional instruments (Azhar et al., 2016; Goldbeck & Storck, 2002; Kodraliu et al., 2001; Patrick et al., 2000; Skevington et al., 2004; Varni et al., 2004; Ware et al., 2000; WHOQOL Group, 1998) and one linear analogue scale (Moons et al., 2006; Williams et al., 1990) were identified. The most frequently used instruments were the 36-Item Short-Form Health Survey (Ware et al., 2000) with five translations across six studies, and the PedsQL Family Impact Module (Varni et al., 2004) assessing both parental health-related quality of life and family functioning across five studies. Additional measures included a study-specific instrument developed for cultural context assessing both parent and parent-proxy reported sibling quality of life (Azhar et al., 2016) was identified, as well as a modified version of the World Health Organization Quality of Life used in a study from Iran concerning caregiver (parent, sibling, aunt/uncle, cousin, grandparent) quality of life (Hussein & Authman, 2013). In addition to the identified quality of life instruments, those assessing family member wellbeing and adjustment (McCubbin & Patterson, 1983) and life satisfaction (Fugl-Meyer et al., 2002) were each identified in one sample (Bratt et al., 2019; Mussatto et al., 2021).

COSMIN criteria were applied to six instruments with a positive rating scored for content validity for five instruments, including the most frequently used 36-Item Short-Form Health Survey and PedsQL Family Impact Module, and internal consistency in two instruments including the 36-Item Short-Form Health Survey. Reliability data were reported for three instruments in four CHD studies (Denniss et al., 2019; Edraki et al., 2014; Hussein & Authman, 2013; Landolt et al., 2011) with internal consistency between .70 and .95.

### Family Impact or Functioning (10 Instruments)

Ten instruments (Epstein et al., 1983; Janus & Goldberg, 1997; McCubbin et al., 1986; 1983; Moos, 1990; Pai et al., 2008; Stein & Jessop, 2003; Stein & Riessman, 1980; Varni et al., 2004; Vinokur & Caplan, 1987) broadly conceptualized as assessing the family system including family functioning or impact of illness on family were identified across 21 (20%) distinct samples (Alkan et al., 2017; Almesned et al., 2013; Brosig et al., 2007a; Cantwell-Bartl & Tibballs, 2017; Davis et al., 1998; Denniss et al., 2019; Doherty et al., 2009; Eagleson et al., 2013; Garcia et al., 2016; Hancock et al., 2018; Hearps et al., 2014; Janus and Goldberg, 1997; Kaugars et al., 2018; Landolt et al., 2011; Lee et al., 2020; Lisanti et al., 2021a; Menahem et al., 2008; Mussatto et al., 2021; Stoffel et al., 2017; Svavarsdottir & McCubbin, 1996; van der Mheen et al., 2019). Two generic multidimensional instruments examining family functioning (Epstein et al., 1983; Moos, 1990), one examining the build-up of stressors (McCubbin et al., 1983) and one examining family hardiness (McCubbin et al., 1986) were identified, as well as illness-focused instruments examining psychosocial risk (Pai et al., 2008), the impact of illness on the family (Stein & Jessop, 2003; Stein & Riessman, 1980; Varni et al., 2004), and a study-specific instrument examining family accommodation of illness (Janus & Goldberg, 1997). Combined, the original and revised versions of the Impact on Family Scale (Stein & Jessop, 2003; Stein & Riessman, 1980) were the most frequently used instruments across seven studies. Use of the original instrument was identified in four studies including one with the sibling subscale (Almesned et al., 2013; Brosig et al., 2007a; Cantwell-Bartl & Tibballs, 2017; Mussatto et al., 2021) and three (Garcia et al., 2016; Landolt et al., 2011; Stoffel et al., 2017) using recommended revised versions with and without additional financial and sibling impact subscales. COSMIN criteria were applied to seven instruments, with five instruments including the most frequently used original and revised versions of the Impact on Family Scale scoring positive ratings for content validity, and the revised version only for internal consistency. In the CHD studies, internal consistency, reported for three instruments in three CHD studies (Denniss et al., 2019; Janus and Goldberg, 1997; Landolt et al., 2011) ranged from .70 to .95.

### Coping (12 Instruments)

Instruments explicitly identifying assessment of coping, including coping self-efficacy and coping appraisal, were categorized in this section. The full, modified version, and subscale-only usage of 12 instruments of coping (Carver, 1997; Carver et al., 1989; Chesney et al., 2003; Endler & Parker, 1994; Folkman & Lazarus, 1985; Frydenberg & Lewis, 2014; Jackson et al., 2017; McCubbin et al., 1981; Murphy et al., 2017; Schreurs, 1993; Sira et al., 2014) were identified across 19 (17%) studies and 18 samples (Ahn et al., 2014; Berant et al., 2001, 2003; Choi & Lee, 2021; Davis 1998; Diffin et al., 2016; Doherty et al., 2009; Hancock et al., 2018; Jackson et al., 2020; Miller et al., 2021; McCusker et al., 2010; Mussatto et al., 2021; Poh et al. 2020; Roberts et al., 2021; Rychik et al., 2013; Sira et al., 2014; Spijkerboer et al. 2007; Svavarsdottir & McCubbin, 1996; Utens et al., 2000). The most frequently used instrument was the Coping Health Inventory for Parents (McCubbin et al., 1981) in five studies across the USA and Singapore, with two studies (Jackson et al., 2020; Sira et al., 2014) using multiple coping instruments. The CHD condition-specific Responses to Stress Questionnaire (Jackson et al., 2017) was identified in one U.S.-based study (Roberts et al., 2021). For this instrument, respondents are required to indicate how stressful CHD-related stressors have been, the degree of control over problems, and their subsequent coping in the past 6 months, with good internal consistency (*α* = .87) and face validity reported in the original study. COSMIN criteria were applied to five instruments including the most frequently used Coping Health Inventory for Parents, with all scoring a positive rating for content validity. Reliability data were reported for five instruments in six CHD studies (Ahn et al., 2014; Berant et al., 2001, 2003; Choi & Lee, 2021; Davis et al., 1998; Sira et al., 2014) with internal consistency between .70 and .95.

### Stress Appraisal (10 Instruments)

Ten instruments assessed stress appraisal, that is, parental/caregiver perception of stressful events across 15 (14%) studies and 14 distinct samples (Berant et al., 2003; Berant et al., 2001; Callahan et al, 2019; Campbell et al., 1986, 1992; Davis et al., 1998; Diffin et al., 2016; Franck et al., 2010; Hoehn et al., 2004; Jackson et al., 2020; Lisanti et al., 2017, 2021a, b; Mussatto et al., 2021; Rahimianfar et al., 2015). Five instruments (Campbell et al., 1986; Miles & Brunssen, 2003; Miles et al., 1993; Rahimianfar et al., 2015; Reid et al., 2007) were used during inpatient admission in 10 studies in total, including the most frequently used Parent Stressor Scale: Infant Hospitalization used in four studies. Five instruments (Folkman & Lazarus, 1985; Holmes & Rahe, 1967; Jackson et al., 2017; Lazarus & Folkman, 1989; Sarason et al., 1978) including the CHD condition-specific Responses to Stress Questionnaire were each used in studies outside of the hospital setting. COSMIN criteria were applied to seven instruments, with a positive rating scored for content validity in five instruments including the Parent Stressor Scale: Infant Hospitalization, internal consistency in one instrument, with two scoring a positive rating for responsiveness. Reliability data were reported for the Parental Stressor Scale: Infant Hospitalization in two CHD studies and the Cognitive Appraisal Scale also in two CHD studies with internal consistency between .70 and .95.

### Couple Relationship Satisfaction or Strain (Two Instruments)

Two instruments assessing the impact on relationship dyads were identified in five (5%) distinct samples (Berant et al., 2003; Bratt et al., 2019; Lisanti et al. 2021a; Riikonen et al., 2019; Rychik et al., 2013). The 32-item Dyadic Adjustment Scale (Spanier, 1976) was used in three studies with no psychometric data reported. Full and shortened translations of the Evaluating and Nurturing Relationship Issues Communication and Happiness Scale (Fowers & Olson, 1989) were used in two studies (Berant et al., 2003; Riikonen et al., 2019) with internal consistency between .70 and .95 in both studies. Family functioning and parenting stress instruments with subscales assessing marriage strain (McCubbin et al., 1983), partner conflict (Abidin, 2012), and the spouse relationship (Abidin, 2012; Östberg et al., 1997) were also identified. COSMIN criteria were applied to the Dyadic Adjustment Scale, with a positive rating scored for content validity and internal consistency.

### Sibling Psychosocial Outcomes (Five Instruments)

Five instruments (Breslau et al., 1981; Lobato & Kao, 2002; Park & Chung, 1996; Varni et al., 1999, 2002) evaluating any sibling psychosocial outcomes and experiences, including health-related quality of life and relationship dynamics were identified across four (4%) distinct samples (Caris et al., 2018; Janus & Goldberg, 1997; Ladak et al., 2019; Moon et al., 2021). The 23-item Sibling Perception Questionnaire (Lobato & Kao, 2002; Sahler & Carpenter, 1989) was used in one study to capture both self and parent-proxy reports of sibling impact (Caris et al., 2018). Use of a Korean translation of the 39-item Sibling Relationship Questionnaire (Park & Chung, 1996) in one study of adolescents, and Urdu translations of the PedsQL Generic Core (23 items) and Cognitive Functioning Scales (6 items) in a comparison study of health-related quality of life in children with CHD (Ladak et al., 2019) also captured self-report data. The four-item Perception of Effect on Sibling Scale (Breslau et al., 1981) used in one study captured parent-reported data only. Three instrument subscales evaluating sibling outcomes (Azhar et al., 2016; Pai et al., 2008; Stein & Riessman, 1980) were also identified across four distinct samples. COSMIN criteria were applied to the Sibling Perception Questionnaire, with a positive rating for content validity and responsiveness achieved. In the CHD studies, internal consistency was reported for four instruments in three studies (Janus & Goldberg, 1997; Ladak et al., 2019; Moon et al., 2021) and was predominantly between .70 and .95.

## Discussion

This systematic review is novel in comprehensively demonstrating the volume and heterogeneity of instruments used to assess psychosocial adaptation and outcomes among parents, caregivers, siblings, and families of children with CHD, which may contribute at least partially to inconsistent results reported across studies (Soulvie et al., 2012; Wei et al., 2015; Woolf-King et al., 2017). Instruments were predominantly self-reported and generic, with just one CHD condition-specific instrument identified. Overall, when applying adapted COSMIN criteria for good measurement properties to accessible original development articles and technical manuals, 67% (36/54) of instruments scored a positive rating for content validity, 39% (21/54) for internal consistency, 9% (5/54) for responsiveness (i.e., longitudinal validity), and 4% (2/54) for test–retest reliability. It should be noted that of the 24 studies reporting test–retest reliability, 22 used Pearson’s Correlation as opposed to the COSMIN-recommended Intra Class Correlation or weighted Kappa, resulting in very few instruments receiving a positive rating. Further, internal consistency data were reported in less than a third of CHD studies and were predominantly between .70 and .95.

Consistent with a review of family instruments used in pediatric psychology (Alderfer et al., 2008), our findings support the identified need for an increased focus on the psychometric properties of instruments used in pediatric populations, where instrument development has often occurred in the general population and reliability and validity data in the specific pediatric sample are lacking. Although several of the identified instruments have been developed for and validated in pediatric illness populations, validation data were identified for the CHD-specific instrument only (Jackson et al., 2017) in the included CHD studies, thereby limiting the availability of potentially useful CHD-specific reference samples. The need for validation of family instruments in pediatric chronic condition samples where normative population scoring may not accurately represent family adaptive processes has previously been identified (Alderfer et al., 2008). Where reported, the development of study-specific instruments in this review was through a review of the literature (Barsella et al., 2021; Janus & Goldberg, 1997), but did not include the target population as recommended in the best practices for establishing content validity. Where instruments were translated from English for the study (*n* = 4) content validity data was reported in one study (Choi & Lee, 2021).

The heterogeneity of instruments identified, coupled with their varying psychometric properties highlight the complex nature of instrument selection in this field. A lack of theoretical framework use has previously been identified (Gregory et al., 2018; Lisanti, 2018), with subsequent use of a wide range of constructs that are not always clearly or consistently defined across studies, amplified by varying study designs. Further, there is a predominance of instruments assessing psychological functioning and distress, with few evaluating positive adaptive constructs such as resilience and post-traumatic growth.

Cultural considerations may further complicate instrument selection. For example, existing validated instruments may be deemed not to align with the demographic and cultural specificities of the study population, with subsequent development of additional study-specific instrument/s as identified in this review (Azhar et al., 2016). Limitations of our review are acknowledged, however, with the restriction of articles to those published in the English language.

The choice of generic, illness-focused, and/or condition-focused instruments is also an important consideration, impacted by research study aim and design. Where illness- and condition-specific instruments enable understanding of the impact of disease, generic instruments allow for the detection of disorders (e.g., depression and post-traumatic stress syndrome), comparison with normative and other pediatric condition cohorts and evaluation, where illness or condition-specific measures are lacking (Shah et al., 2021). Potential limitations of generic instrument use alone are identified in the current review in the context of assessing parenting stress in families of children with CHD, with generic and illness-focused instruments not consistently used together as recommended. The limitation of generic instruments alone to detect associations between parenting stress and illness factors identified by illness-focused instruments identified in an earlier review of parenting stress (Cousino & Hazen, 2013), as well as qualitative interviews, are also recognized in CHD family studies (Carey et al., 2002; Jantien Vrijmoet-Wiersma et al., 2009; Kaugars et al., 2018). Decisions regarding instrument choice may also be determined by whether the primary focus of the study was on child outcomes or the impact on the parent/caregiver/family.

Outdated instrument use is also identified in the current review. Originally developed as a four-factor 24-item instrument (Stein & Riessman, 1980), inclusive of six items to assess the impact on siblings, the Impact on Family Scale was revised in 2003. Now a one-factor, 15-item instrument with excellent psychometric properties, the authors explicitly advocate for the use of the revised version (Stein & Jessop, 2003). Question sets to measure financial and sibling impact are available, however, significantly weaker psychometric properties are described. The use of the original instrument in studies of families of children with CHD has continued, however, with another using the revised version with additional financial and sibling impact question sets.

Methodological strengths of the current systematic review include the comprehensive conceptualization of the diverse constructs informed by theoretical frameworks and the use of the “gold standard” COSMIN criteria to evaluate good measurement properties. However, limitations to the current review are acknowledged when interpreting findings. Restriction to studies with CHD samples only may have resulted in the potential exclusion of some instruments. Additionally, instruments that may be of benefit to assessing families of children with CHD but have not been previously used in this population will not be identified. While the selected inclusion of COSMIN criteria is of importance, we are unable to comment on other measurement properties, including cross-cultural validity, as these were not evaluated. Further to the application of COSMIN criteria (to original instrument development articles/manuals), additional validation papers and reported psychometric properties, including those in pediatric populations, will not have been included. Analysis of associations between the methodological quality of included studies and quality of their instruments was limited by the number of instruments used per study, different scores based on COSMIN criteria, and lack of application of the criteria to some instruments and was therefore not undertaken.

Despite these limitations, this systematic review provides the most comprehensive catalog of instruments used to assess psychosocial adaptation and outcomes among parents, caregivers, siblings, and families of children with CHD to date, including critical evaluation of instrument characteristics and psychometric properties. Based on the synthesis of our findings, the following key recommendations are proposed to facilitate future instrument selection and use in pediatric CHD research and clinical settings.

1. In addition to theoretical frameworks, instrument selection should be informed by robust key psychometrics (including content validity and internal consistency), and those relevant to the CHD population wherever available. The rationale for instrument choice should be reported to enable contextualization of findings across research and clinical practice. This will be particularly valuable for new research focus areas such as positive adaptive processes of individual and family resilience capacities and post-traumatic growth.
2. Validation of instruments in CHD populations should be prioritized, particularly the frequently used and methodologically robust instruments identified in the current review and those recommended by expert collaborative groups, such as the Cardiac Neurodevelopmental Outcome Collaborative (Ilardi et al., 2020; Ware et al., 2020). Furthermore, routine reporting of psychometric properties in all CHD studies is needed, including content validity for study-specific instruments. To improve the content validity of study-specific instruments, the involvement of the target population in the instrument development process is emphasized along with expert panels and information based on literature reviews.
3. Development of a core set of outcome instruments should be prioritized to facilitate consistency of instrument use and subsequent synthesis of results across studies. From a clinical perspective, with the call to embed psychosocial screening and assessment as part of the usual congenital cardiac care, adopting a “toolkit” approach to instrument selection is recommended. This may be informed by the current review as well as existing reviews of instruments used in pediatric populations and psychosocial standards of care and guidelines. It may be further informed by established approaches, including the Patient-Reported Outcomes Measurement System (PROMIS), and may complement the International Consortium for Health Outcomes Measurement (ICHOM) Congenital Heart Disease Working Group’s recently developed standard set of clinical and patient-reported outcomes for use in routine clinical practice (Hummel et al., 2021). A core set or “toolkit” approach should be inclusive of both generic and illness-focused instruments for use in screening and assessment with the individual and broader family system across the lifespan, including diverse ages, developmental stages, disease types, and transitions. Instruments should also be sensitive to both culturally and neurodiverse populations. Finally, core sets/“toolkits” should be regularly reviewed for updated versions of included instruments and to prevent the use of outdated versions.
4. To enhance generalizability, adopting clear guidelines for methodologically robust adaptation or substitution of instruments across different social, economic, and cultural contexts is recommended.
5. With very limited CHD-specific measures identified, the development of a multidimensional instrument adaptable to diverse social, economic, and cultural contexts for use in both CHD research and clinical settings to assess the impact on families is also needed. A multi-informant approach inclusive of siblings to enable a comprehensive assessment of the individual and family system should be considered (Pritchett et al., 2011).

## Conclusion and Future Directions

Characteristics and use of a broad range of available instruments to quantitatively assess the impact and adaptation to CHD in family members (mothers, fathers, and siblings) and the family system may be contributors to current challenges in understanding the unique experiences of this high-risk population. Prioritization of key recommendations for instrument selection and use described in this systematic review will be beneficial to extend current knowledge and improve care to all family members across the continuum.

## Supplementary Material

jsad015_Supplementary_Data

## Data Availability

Datasets generated and/or analyzed for this study will be available on reasonable request from the corresponding author.
