# Supplementary material for: Systematic Review of Instruments Assessing Psychosocial Adaptation and Outcomes Among Families of Children With Congenital Heart Disease
Source: J Pediatr Psychol. 2023 May 23;48(6):537–52. doi: 10.1093/jpepsy/jsad015 (PMC10321401; doi:10.1093/jpepsy/jsad015)
Supplement: jsad015_Supplementary_Data [file jsad015_supplementary_data.zip › jsad015_Supplementary_Data/Eagleson et al. Journal of Pediatric Psychology 2023 Supplementary Data.pdf]

Eagleson et al., Systematic Review of Instruments Assessing Psychosocial Adaptation and Outcomes Among Families of Children With Congenital Heart Disease, *Journal of Pediatric Psychology*, 2023.

Supplementary Materials:

S1: Search terms and database search strings.

Figure S2: Preferred Reporting Items for Systematic Reviews and Meta-Analyses (PRISMA) flow diagram of the study selection process.

Table S3. Study methodological characteristics.

Figure S4: Frequency of instrument usage across studies.

Table S5: Instrument characteristics.

Table S6: Instrument psychometric properties.

S1: Search terms and database search strings.

Population	Exposure	Outcome
Family	Congenital Heart Disease	Stress
Families	Congenital Heart Defects	Psychological Stress
Parent	CHD	Distress
Parents		Coping
Mother		Coping Styles
Mothers		Coping Strategies
Maternal		Coping Patterns
Father		Social Support
Fathers		Impact
Paternal		Psychological Function
Sibling		Psychological Trauma
Siblings		Mental Health
Caregiver		Communication
Caregivers		Parenting
Carer		Relationships
Carers		Finances
		Schooling
		Employment
		Wellbeing
		Quality of Life
		QOL
		Health Related Quality of Life
		HRQOL
		Psychological Adaptation
		Adaptation
		Lived Experience

PubMed/MEDLINE search string:

((((((((((((((((((((((carer[Title/Abstract]) OR carers[Title/Abstract]) OR caregiver[Title/Abstract]) OR caregivers[Title/Abstract]) OR caregivers[MeSH Terms]) OR sibling[Title/Abstract]) OR siblings[Title/Abstract]) OR siblings[MeSH Terms]) OR paternal[Title/Abstract]) OR father[Title/Abstract]) OR fathers[Title/Abstract]) OR fathers[MeSH Terms]) OR maternal[Title/Abstract]) OR mother[Title/Abstract]) OR mothers[Title/Abstract]) OR mothers[MeSH Terms]) OR parent[Title/Abstract]) OR parents[Title/Abstract]) OR parents[MeSH Terms]) OR families[Title/Abstract]) OR family[Title/Abstract]) OR family[MeSH Terms]) AND ((((CHD[Title/Abstract]) OR “congenital heart defects”[Title/Abstract]) OR congenital heart defects[MeSH Terms]) OR “congenital heart disease”[Title/Abstract])) AND (((((((((((((((((((((((((((((((("lived experience"[Title/Abstract]) OR adaptation[Title/Abstract]) OR psychological adaptation[MeSH Terms]) OR HRQOL[Title/Abstract]) OR "health related quality of life"[Title/Abstract]) OR QOL[Title/Abstract]) OR "quality of life"[Title/Abstract]) OR wellbeing[Title/Abstract]) OR employment[Title/Abstract]) OR employment[MeSH Terms]) OR schooling[Title/Abstract]) OR finances[Title/Abstract]) OR relationships[Title/Abstract]) OR parenting[Title/Abstract]) OR parenting[MeSH Terms]) OR communication[Title/Abstract]) OR communication[MeSH Terms]) OR "mental health"[Title/Abstract]) OR mental health[MeSH Terms]) OR psychological trauma[MeSH Terms]) OR "psychological function*"[Title/Abstract]) OR impact[Title/Abstract]) OR "social support"[Title/Abstract]) OR social support[MeSH Terms]) OR "coping patterns"[Title/Abstract]) OR "coping strategies"[Title/Abstract]) OR "coping

styles"[Title/Abstract]) OR coping[Title/Abstract]) OR distress[Title/Abstract]) OR psychological stress[MeSH Terms]) OR stress[Title/Abstract])) AND English[lang]

CINAHL search string:

S64 S61 AND S62 AND S63

S63 S23 OR S28 OR S29 OR S30 OR S31 OR S32 OR S33 OR S34 OR S35 OR S36 OR S37 OR S38 OR S39 OR S40 OR S41 OR S42 OR S43 OR S44 OR S45 OR S46 OR S47 OR S48 OR S49 OR S50 OR S51 OR S52 OR S53 OR S54 OR S55 OR S56 OR S57 OR S58 OR S59 OR S60

S62 S24 OR S25 OR S26 OR S27

S61 S1 OR S2 OR S3 OR S4 OR S5 OR S6 OR S7 OR S8 OR S9 OR S10 OR S11 OR S12 OR S13 OR S14 OR S15 OR S16 OR S17 OR S18 OR S19 OR S20 OR S21 OR S22

S60 TI impact OR AB impact

S59 TI "psychological function*" OR AB "psychological function*"

S58 TI "social support" OR AB "social support"

S57 TI "lived experience" OR AB "lived experience"

S56 TI adaptation OR AB adaptation

S55 MH adaptation, psychological

S54 TI HRQOL OR AB HRQOL

S53 TI "health related quality of life" OR AB "health related quality of life"

S52 TI QOL OR AB QOL

S51 TI "quality of life" OR AB "quality of life"

S50 MH quality of life

S49 TI wellbeing OR AB wellbeing

S48 MH psychological well-being

S47 TI employment OR AB employment

S46 MH employment

S45 TI schooling OR AB schooling

S44 TI finance OR AB finance

S43 TI relationships OR AB relationships

S42 TI parenting OR AB parenting

S41 MH parenting

S40 TI communication OR AB communication

S39 MH communication

S38 TI "mental health" OR AB "mental health"

S37 MH mental health

S36 MH psychological trauma

S35	TI "coping strategies" OR AB "coping strategies"
S34	TI "coping styles" OR AB "coping styles"
S33	TI coping OR AB coping
S32	MH coping
S31	MH stress, psychological
S30	TI stress OR AB stress
S29	MH stress
S28	TI distress OR AB distress
S27	TI CHD OR AB CHD
S26	TI "congenital heart defects" OR AB "congenital heart defects"
S25	MH heart defects, congenital
S24	TI "congenital heart disease" OR AB "congenital heart disease"
S23	TI "coping patterns" OR AB "coping patterns"
S22	TI carers OR AB carers
S21	TI carer OR AB carer
S20	TI caregiver OR AB caregiver
S19	TI caregivers OR AB caregivers
S18	MH caregivers
S17	TI sibling OR AB sibling
S16	TI siblings OR AB siblings
S15	MH siblings
S14	TI paternal OR AB paternal
S13	TI father OR AB father
S12	TI fathers OR AB fathers
S11	MH fathers
S10	TI maternal OR AB maternal
S9	TI mother OR AB mother
S8	TI mothers OR AB mothers
S7	MH mothers
S6	TI parent OR AB parent
S5	TI parents OR AB parents
S4	MH parents
S3	TI families OR AB families
S2	TI family OR AB family
S1	MH family

PsycINFO search string:

(((((title: (carer))) *OR* ((abstract: (carer)))) *AND* (PublicationTypeFilt: ("Peer Reviewed Journal"))) *OR* ((((title: (carers))) *OR* ((abstract: (carers)))) *AND* (PublicationTypeFilt: ("Peer Reviewed Journal"))) *OR* ((((title: (caregiver))) *OR* ((abstract: (caregiver)))) *AND* (PublicationTypeFilt: ("Peer Reviewed Journal"))) *OR* ((((title: (caregivers))) *OR* ((abstract: (caregivers)))) *AND* (PublicationTypeFilt: ("Peer Reviewed Journal"))) *OR* ((((Index Terms: (caregivers)))) *AND* (PublicationTypeFilt: ("Peer Reviewed Journal"))) *OR* ((((title: (sibling))) *OR* ((abstract: (sibling)))) *AND* (PublicationTypeFilt: ("Peer Reviewed Journal"))) *OR* ((((title: (siblings))) *OR* ((abstract: (siblings)))) *AND* (PublicationTypeFilt: ("Peer Reviewed Journal"))) *OR* ((((Index Terms: (siblings)))) *AND* (PublicationTypeFilt: ("Peer Reviewed Journal"))) *OR* ((((title: (paternal))) *OR* ((abstract: (paternal)))) *AND* (PublicationTypeFilt: ("Peer Reviewed Journal"))) *OR* ((((title: (families))) *OR* ((abstract: (families)))) *AND* (PublicationTypeFilt: ("Peer Reviewed Journal"))) *OR* ((((title: (father))) *OR* ((abstract: (father)))) *AND* (PublicationTypeFilt: ("Peer Reviewed Journal"))) *OR* ((((title: (mother))) *OR* ((abstract: (mother)))) *AND* (PublicationTypeFilt: ("Peer Reviewed Journal"))) *OR* ((((title: (mothers))) *OR* ((abstract: (mothers)))) *AND* (PublicationTypeFilt: ("Peer Reviewed Journal"))) *OR* ((((Index Terms: (mothers)))) *AND* (PublicationTypeFilt: ("Peer Reviewed Journal"))) *OR* ((((title: (family))) *OR* ((abstract: (family)))) *AND* (PublicationTypeFilt: ("Peer Reviewed Journal"))) *OR* ((((Index Terms: (family)))) *AND* (PublicationTypeFilt: ("Peer Reviewed Journal"))) *OR* ((((title: (maternal))) *OR* ((abstract: (maternal)))) *AND* (PublicationTypeFilt: ("Peer Reviewed Journal"))) *OR* ((((Index Terms: (fathers)))) *AND* (PublicationTypeFilt: ("Peer Reviewed Journal"))) *OR* ((((title: (fathers))) *OR* ((abstract: (fathers)))) *AND* (PublicationTypeFilt: ("Peer Reviewed Journal")))) *AND* (((((title: ("congenital heart disease"))) *OR* ((abstract: ("congenital heart disease")))) *AND* (PublicationTypeFilt: ("Peer Reviewed Journal"))) *OR* ((((title: ("congenital heart defects"))) *OR* ((abstract: ("congenital heart defects")))) *AND* (PublicationTypeFilt: ("Peer Reviewed Journal"))) *OR* ((((title: (CHD))) *OR* ((abstract: (CHD)))) *AND* (PublicationTypeFilt: ("Peer Reviewed Journal")))) *AND* (((((title: ("lived experience"))) *OR* ((abstract: ("lived experience")))) *AND* (PublicationTypeFilt: ("Peer Reviewed Journal"))) *OR* ((((title: (adaptation))) *OR* ((abstract: (adaptation)))) *AND* (PublicationTypeFilt: ("Peer Reviewed Journal"))) *OR* ((((title: ("psychological adaptation"))) *OR* ((abstract: ("psychological adaptation")))) *AND* (PublicationTypeFilt: ("Peer Reviewed Journal"))) *OR* ((((title: (HRQOL))) *OR* ((abstract: (HRQOL)))) *AND* (PublicationTypeFilt: ("Peer Reviewed Journal"))) *OR* ((((title: ("health related quality of life"))) *OR* ((abstract: ("health related quality of life")))) *AND* (PublicationTypeFilt: ("Peer Reviewed Journal"))) *OR* ((((title: (QOL))) *OR* ((abstract: (QOL)))) *AND* (PublicationTypeFilt: ("Peer Reviewed Journal"))) *OR* ((((title: ("quality of life"))) *OR* ((abstract: ("quality of life")))) *AND* (PublicationTypeFilt: ("Peer Reviewed Journal"))) *OR* ((((Index Terms: ("quality of life")))) *AND* (PublicationTypeFilt: ("Peer Reviewed Journal"))) *OR* ((((title: (wellbeing))) *OR* ((abstract: (wellbeing)))) *AND* (PublicationTypeFilt: ("Peer Reviewed Journal"))) *OR* ((((title: (employment))) *OR* ((abstract: (employment)))) *AND* (PublicationTypeFilt: ("Peer Reviewed Journal"))) *OR* ((((Index Terms: (employment)))) *AND* (PublicationTypeFilt: ("Peer Reviewed Journal"))) *OR* ((((title: (schooling))) *OR* ((abstract: (schooling)))) *AND* (PublicationTypeFilt: ("Peer Reviewed Journal"))) *OR* ((((title: (finance))) *OR* ((abstract: (finance)))) *AND* (PublicationTypeFilt: ("Peer Reviewed Journal"))) *OR* ((((title: (relationships))) *OR* ((abstract: (relationships)))) *AND* (PublicationTypeFilt: ("Peer Reviewed Journal"))) *OR* ((((title: (parenting))) *OR* ((abstract: (parenting)))) *AND* (PublicationTypeFilt: ("Peer Reviewed Journal"))) *OR* ((((Index Terms: (parenting)))) *AND* (PublicationTypeFilt: ("Peer Reviewed Journal"))) *OR* ((((title: (communication))) *OR* ((abstract: (communication)))) *AND* (PublicationTypeFilt: ("Peer Reviewed Journal"))) *OR* ((((Index Terms: (communication)))) *AND* (PublicationTypeFilt: ("Peer Reviewed Journal"))) *OR* ((((title: ("mental health"))) *OR* ((abstract: ("mental health")))) *AND* (PublicationTypeFilt: ("Peer Reviewed Journal"))) *OR* ((((Index Terms: (mental health)))) *AND* (PublicationTypeFilt: ("Peer Reviewed Journal"))) *OR* ((((title: ("psychological trauma"))) *OR* ((abstract: ("psychological trauma")))) *AND* (PublicationTypeFilt: ("Peer Reviewed Journal"))) *OR* ((((title: ("psychological function*"))) *OR* ((abstract: ("psychological function*")))) *AND* (PublicationTypeFilt: ("Peer Reviewed Journal"))) *OR* ((((title: (impact))) *OR* ((abstract: (impact)))) *AND* (PublicationTypeFilt: ("Peer Reviewed Journal"))) *OR* ((((title: ("social support"))) *OR* ((abstract: ("social support")))) *AND* (PublicationTypeFilt: ("Peer Reviewed Journal"))) *OR* ((((Index Terms: ("social support")))) *AND* (PublicationTypeFilt: ("Peer Reviewed Journal"))) *OR* ((((title: ("coping patterns"))) *OR* ((abstract: ("coping patterns")))) *AND* (PublicationTypeFilt: ("Peer Reviewed

Journal"))) *OR* ((((title: ("coping strategies"))) *OR* ((abstract: ("coping strategies")))) *AND* (PublicationTypeFilt: ("Peer Reviewed Journal"))) *OR* ((((title: ("coping styles"))) *OR* ((abstract: ("coping styles")))) *AND* (PublicationTypeFilt: ("Peer Reviewed Journal"))) *OR* ((((title: (coping))) *OR* ((abstract: (coping)))) *AND* (PublicationTypeFilt: ("Peer Reviewed Journal"))) *OR* ((((Index Terms: (coping)))) *AND* (PublicationTypeFilt: ("Peer Reviewed Journal"))) *OR* ((((title: (distress))) *OR* ((abstract: (distress)))) *AND* (PublicationTypeFilt: ("Peer Reviewed Journal"))) *OR* ((((Index Terms: (distress)))) *AND* (PublicationTypeFilt: ("Peer Reviewed Journal"))) *OR* ((((Index Terms: (psychological stress)))) *AND* (PublicationTypeFilt: ("Peer Reviewed Journal"))) *OR* ((((title: (stress))) *OR* ((abstract: (stress)))) *AND* (PublicationTypeFilt: ("Peer Reviewed Journal"))) *OR* ((((Index Terms: (stress)))) *AND* (PublicationTypeFilt: ("Peer Reviewed Journal"))))

Embase search string:

('family'/exp OR 'family':ab,ti OR 'families':ab,ti OR 'parent'/exp OR 'parent':ab,ti OR 'parents':ab,ti OR 'mother'/exp OR 'mother':ab,ti OR 'mothers':ab,ti OR 'maternal':ab,ti OR 'father'/exp OR 'father':ab,ti OR 'fathers':ab,ti OR 'paternal':ab,ti OR 'sibling'/exp OR 'sibling':ab,ti OR 'siblings':ab,ti OR 'caregiver'/exp OR 'caregiver':ab,ti OR 'caregivers':ab,ti OR 'carer':ab,ti OR 'carers':ab,ti) AND ('congenital heart malformation'/exp OR 'congenital heart malformation':ab,ti OR 'chd':ab,ti) AND ('stress'/exp OR 'stress':ab,ti OR 'mental stress'/exp OR 'distress':ab,ti OR 'coping behavior'/exp OR 'coping':ab,ti OR 'coping styles':ab,ti OR 'coping strategies':ab,ti OR 'coping patterns':ab,ti OR 'impact':ab,ti OR 'psychological function*':ab,ti OR 'mental health'/exp OR 'mental health':ab,ti OR 'wellbeing'/exp OR 'wellbeing':ab,ti OR 'quality of life'/exp OR 'quality of life':ab,ti OR 'qol':ab,ti OR 'health related quality of life':ab,ti OR 'hrqol':ab,ti OR 'psychological adaptation':ab,ti OR 'adaptation':ab,ti OR 'psychotrauma'/exp OR 'lived experience':ab,ti OR 'communication':ab,ti OR 'parenting':ab,ti OR 'relationships':ab,ti OR 'finance':ab,ti OR 'schooling':ab,ti OR 'education'/exp OR 'education':ab,ti) AND [english]/lim

SCOPUS search string:

((TITLE-ABS-KEY (family)) OR (TITLE-ABS-KEY (parent)) OR (TITLE-ABS-KEY (mother)) OR (TITLE-ABS-KEY (maternal)) OR (TITLE-ABS-KEY (father)) OR (TITLE-ABS-KEY (paternal)) OR (TITLE-ABS-KEY (sibling)) OR (TITLE-ABS-KEY (caregiver)) OR (TITLE-ABS-KEY (carer))) AND ((TITLE-ABS-KEY ("congenital heart disease")) OR (TITLE-ABS-KEY ("congenital heart defects")) OR (TITLE-ABS-KEY (chd))) AND ((TITLE-ABS-KEY (distress)) OR (TITLE-ABS-KEY (coping)) OR (TITLE-ABS-KEY ("coping styles")) OR (TITLE-ABS-KEY ("coping strategies")) OR (TITLE-ABS-KEY ("coping patterns")) OR (TITLE-ABS-KEY (impact)) OR (TITLE-ABS-KEY ("psychological function*")) OR (TITLE-ABS-KEY ("psychological trauma")) OR (TITLE-ABS-KEY ("mental health")) OR (TITLE-ABS-KEY (communication)) OR (TITLE-ABS-KEY ("social support")) OR (TITLE-ABS-KEY (parenting)) OR (TITLE-ABS-KEY (relationships)) OR (TITLE-ABS-KEY (finance)) OR (TITLE-ABS-KEY (schooling)) OR (TITLE-ABS-KEY (employment)) OR (TITLE-ABS-KEY (wellbeing)) OR (TITLE-ABS-KEY ("quality of life")) OR (TITLE-ABS-KEY ("health related quality of life")) OR (TITLE-ABS-KEY ("psychological adaptation")) OR (TITLE-ABS-KEY (adaptation)) OR (TITLE-ABS-KEY ("lived experience"))) AND (LIMIT-TO (LANGUAGE , "English"))

Figure S2: Preferred Reporting Items for Systematic Reviews and Meta-Analyses (PRISMA) flow diagram of the study selection process.

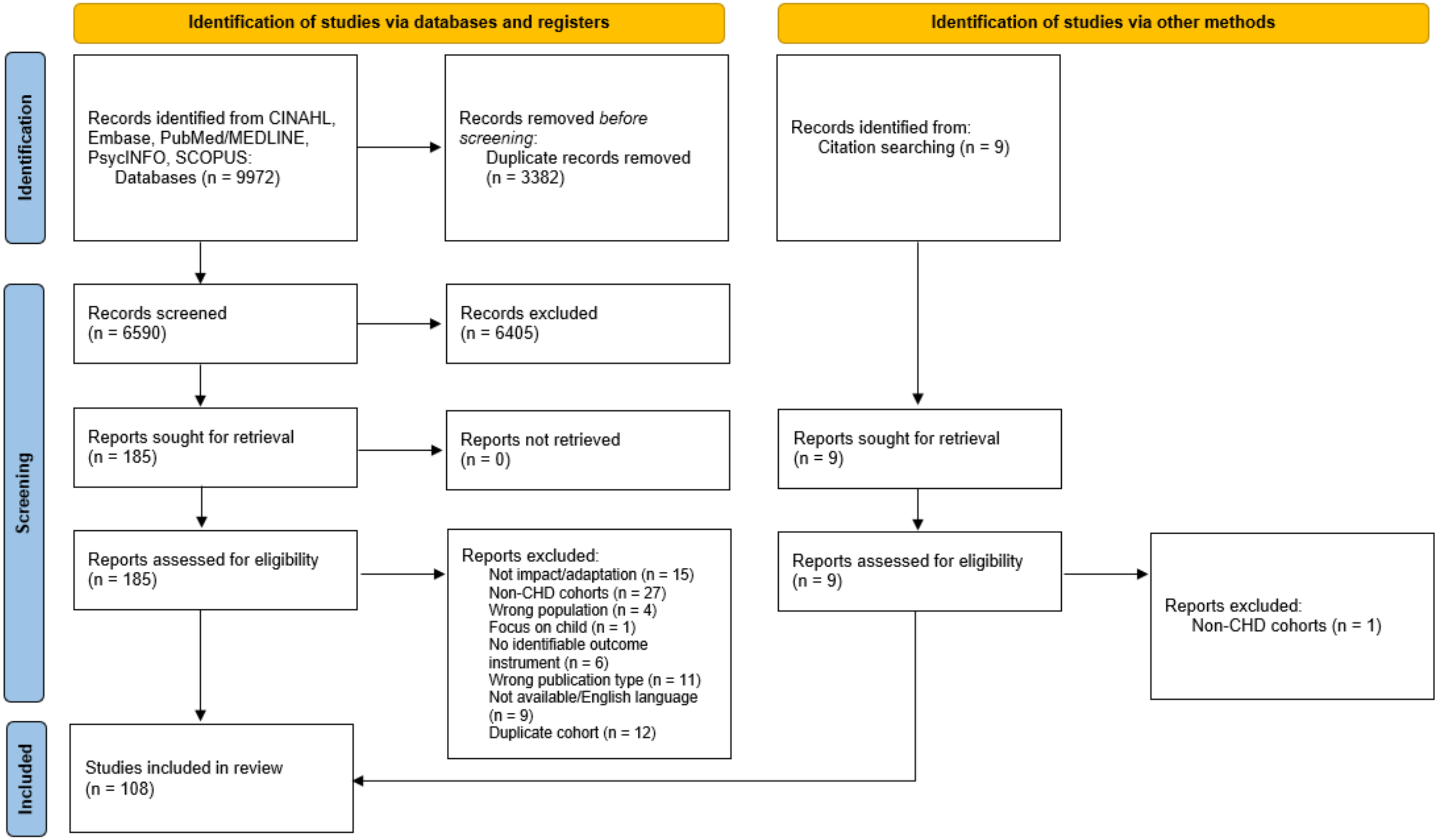

Table S3: Study methodological characteristics.

Study				Child with CHD		Parent/Family				Conceptual Framework Identified	Quality Appraisal
(Reference) Study Location	Relevant Construct/s	Relevant Instrument/s	Response Rate	Diagnosis	Age, Mean ± SD (Range)	Respondents	Age, Mean ± SD (Range), Years	CHD Sample Size	% Fathers /Male Caregiver		
(Ahn et al., 2014) Korea	Coping	Coping Inventory for Stressful Situations (CISS; Korean translation)	N/R	CHD	(12–21 yrs)	Parents	(39–57)	40 Parents	17.5% Male	No	Fair
(Alkan et al., 2017) Turkey	Quality of Life	36-Item Short Form Health Survey (SF-36; Turkish version)	N/R	Cyanotic and Acyanotic CHD	Cyanotic: 10.2 ± 2.7; Acyanotic: 9.6 ± 3.1 yrs	Mothers	Cyanotic: 37.4 ± 6.4; Acyanotic 36.2 ± 6.9	80 Mothers (40 Cyanotic 40 Acyanotic)	N/A	No	Fair
	Anxiety; Depression	Hospital Anxiety and Depression Scale (HADS; Turkish version)									
	Family Functioning	Family Assessment Device (FAD; Turkish version)									
(Almesned et al., 2013) Saudi Arabia	Family Impact	Impact on Family Scale (IOFS; Original version)	100% Recruitment	Complex and Simple CHD >6months since diagnosis	N/R	Parents	N/R	41 Parents (21 Complex 20 Simple)	N/R	No	Good
(Awaad & Darahim, 2015) Egypt	Depression	Beck Depression Inventory (BDI; Arabic version)	92% Participation (115/125)	CHD	5.5 ± 5.1 yrs	Caregivers	N/R	115 Caregivers	N/R	No	Good
	Anxiety	Taylor Manifest Anxiety Scale (TMAS)									

Study				Child with CHD		Parent/Family				Conceptual Framework Identified	Quality Appraisal
**(Reference) Study Location**	**Relevant Construct/s**	**Relevant Instrument/s**	**Response Rate**	**Diagnosis**	**Age, Mean ± SD (Range)**	**Respondents**	**Age, Mean ± SD (Range), Years**	**CHD Sample Size**	**% Fathers /Male Caregiver**		
	Psychiatric Diagnosis	Structured Clinical Interview for DSM-IV (Arabic version)									
(Azhar et al., 2016) Saudi Arabia	Quality of Life	Study-Specific Instrument including parental and sibling Quality of Life and families' needs and expectations	N/R	CHD	5.7 ± 4.8 yrs	Families	N/R	180 Families	N/R	No	Good
(Barsella et al., 2021) USA	Parental Stress	Study-Specific Instrument	N/R	CHD post cardiac surgery	21 months–8 yrs	Parents	(26–51+)	3 Parents	NR	No	Poor
(Bektas et al., 2020) Turkey	Caregiver Burden	Zarit Caregiver Burden Interview (ZBI; Turkish version)	N/R	CHD	6.8 ± 5.4 yrs	Parents	34.8 ± 7.9	124 Parents	8.1% (10/124) Fathers	No	Good
	Quality of Life	World Health Organization Quality of Life-BREF (WHOQOL-BREF)									
(Berant et al., 2001) Israel	Cognitive Appraisal of Stress and Coping (tailored to motherhood)	Cognitive Appraisal Scale	N/R	Severe and Mild CHD	3 months average at diagnosis	Mothers	30.1 (Mean CHD group)	101 Mothers	N/A	Yes	Good
	Coping	Ways of Coping Checklist (modified to focus on									

Study				Child with CHD		Parent/Family				Conceptual Framework Identified	Quality Appraisal
(Reference) Study Location	Relevant Construct/s	Relevant Instrument/s	Response Rate	Diagnosis	Age, Mean ± SD (Range)	Respondents	Age, Mean ± SD (Range), Years	CHD Sample Size	% Fathers /Male Caregiver		
		motherhood tasks; Hebrew version)									
	Psychological Distress	Mental Health Inventory (MHI; Hebrew version)									
(Berant et al., 2003) Israel	Cognitive Appraisal of Stress and Coping (tailored to motherhood)	Cognitive Appraisal Scale	82% at Time 1; 85% Retention at Time 2	CHD	3 months average at diagnosis + 1 yrs	Mothers	30.3 ± 5.4 at Time 2	85 Mothers	N/A	Yes	Good
	Coping	Ways of Coping Checklist (modified; Hebrew version)									
	Marital Satisfaction	Evaluating and Nurturing Relationship Issues Communication and Happiness Scale (ENRICH; shortened Hebrew version)									
(Bevilacqua et al., 2013) Italy	Psychological Distress	General Health Questionnaire-30 (GHQ-30; Italian version)	78% Participation (38/49)	Severe CHD	Infants	Parent Couples	Mothers: 33.3 ± 5.5; Fathers 36.4 ± 6.7	38 Parent Couples	95% (36/38) Fathers	No	Good
	Depression	Beck Depression Inventory-Second Edition (BDI-2; Italian version);									

Study				Child with CHD		Parent/Family				Conceptual Framework Identified	Quality Appraisal
(Reference) Study Location	Relevant Construct/s	Relevant Instrument/s	Response Rate	Diagnosis	Age, Mean ± SD (Range)	Respondents	Age, Mean ± SD (Range), Years	CHD Sample Size	% Fathers /Male Caregiver		
	Health-Related Quality of Life	36-Item Short Form Health Survey (SF-36; language unspecified)									
(Bishop et al., 2019) USA	Psychological Adjustment	Brief Symptom Inventory 18 (BSI-18)	N/R	CHD	18.4 ± 14.03 months (15 days–3 yrs)	Parents	33.2 ± 6.7	69 Parents	8.7% (6/69) Fathers	Yes	Fair
	Parenting Stress	Pediatric Inventory for Parents (PIP)									
(Blue et al., 2015) Australia	Depression, Anxiety, Stress	Depression, Anxiety and Stress Scale-21 Items (DASS-21)	91.5% Response; 60.6% Participation	CHD requiring elective surgery	N/R	Parents	34.1 ± 5.8	55 Parents	21.9% (12/55) Fathers	No	Fair
	Guilt and Shame	Personal Feelings Questionnaire (PFQ-2)									
	Emotional Feelings	Emotional Aspects of Having a Child with CHD									
(Bratt et al., 2019) Sweden	Anxiety; Depression	Hospital Anxiety and Depression Scale (HADS; Swedish version)	82% Prenatal; 100% Postnatal group	Prenatal and Postnatal CHD Diagnosis	(2–6 months) at Time 2	Parents	Time 1: 31.5 ± 4.1 (Prenatal); Time 2: 32.8 ± 3.3 (Prenatal), 32.8 ± 5.2 (Postnatal)	Time 1: 28 Parents (Prenatal); Time 2: 15 Parents (Prenatal), 30 Parents (Postnatal)	Time 1: 50% (14/28) Fathers (Prenatal); Time 2: 53.3% (8/15) (Prenatal), 50% (15/30) (Postnatal)	No	Good
	Life Satisfaction	Life Satisfaction Questionnaire (LiSat-11)									
	Relationship Satisfaction	Dyadic Adjustment Scale (DAS)									

Study				Child with CHD		Parent/Family				Conceptual Framework Identified	Quality Appraisal
(Reference) Study Location	Relevant Construct/s	Relevant Instrument/s	Response Rate	Diagnosis	Age, Mean ± SD (Range)	Respondents	Age, Mean ± SD (Range), Years	CHD Sample Size	% Fathers /Male Caregiver		
(Brosig, et al., 2007a) USA	Family Impact	Impact on Family Scale (IOFS; Original version)	53%	TGA and HLHS	4.7 yrs ± 10 months	Parents	N/R	26 Parents (13 TGA; 13 HLHS)	N/R	No	Fair
	Parenting Stress	Parenting Stress Index (PSI)									
(Brosig, et al., 2007b) USA	Psychological Distress	Brief Symptom Inventory (BSI)	91% Prenatal; 44% Postnatal	CHD	6 months post birth at Time 2	Parent Couples	N/R	17 Couples (10 Prenatal; 7 Postnatal)	100% (17/17)	No	Fair
(Callahan et al., 2019) USA	Psychological Distress/Stress Appraisal	Neonatal Unit Parental Stressor Scale (NUPS)	89% (81/91) Participation	CHD	Neonates	Parents	(<20–>40)	53 Families including 30 Couples (48 Mothers; 29 Fathers)	35% (29/77)	No	Fair
	Depression, Anxiety, Stress	Depression, Anxiety and Stress Scale-21 Items (DASS-21)									
(Campbell et al., 1986) USA	Tension/Stress	Feeling of Tension Questionnaire	N/R	CHD undergoing cardiac catheter	6–17 yrs	Parents	N/R	N/R	N/R	No	Poor
(Campbell et al., 1992) USA	Anxious Behavior	Caregivers' Behavior Rating Scale (CBRS)	N/R	CHD undergoing cardiac catheter	1–4 yrs	Mothers	N/R	50 Mothers	N/A	No	Poor
	Anxiety	State-Trait Anxiety Inventory (STAI)									
	Tension/Stress	Feeling of Tension Questionnaire									
(Campbell et al., 1995) USA	Anxious Behavior	Caregivers' Behavior Rating Scale (CBRS)	92% (48/52) Participation	CHD undergoing cardiac surgery	7 ± 3 yrs	Primary Caregivers	N/R	48 Primary Caregivers (46 Mothers; 2 Fathers)	4% (2/48)	Yes	Fair
	Anxiety	State-Trait Anxiety Inventory (STAI)									

Study				Child with CHD		Parent/Family				Conceptual Framework Identified	Quality Appraisal
(Reference) Study Location	Relevant Construct/s	Relevant Instrument/s	Response Rate	Diagnosis	Age, Mean ± SD (Range)	Respondents	Age, Mean ± SD (Range), Years	CHD Sample Size	% Fathers /Male Caregiver		
(Cantwell-Bartl & Tibballs, 2017) USA	Psychological Burnout	Maslach Burnout Inventory (MBI)	Mothers 73% (16/22); Fathers 59% (13/22)	HLHS	(1–19 yrs)	Parent/ Couples	4 Parents <25 years reported only	16 Couples; 3 Mothers	81.2% (13/16)	No	Fair
	Family Impact	Impact on Family Scale (IOFS; Original version)									
	Psychological Trauma and Post Traumatic Stress Disorder	PTSD Checklist-Civilian Version (PCL-C)									
(Carey et al., 2002) USA	Parenting Stress	Parenting Stress Index-Short Form (PSI-SF)	77% (30/39)	CHD	3.4 ± 0.9 yrs	Mothers	33.5 ± 4.5	39 Mothers	N/A	Yes	Fair
(Caris et al., 2016) USA	Parenting Stress	Parenting Stress Index-Short Form (PSI-SF)	N/R	HLHS	4.9 ± 5.2 yrs	Caregivers	N/R	459 Caregivers	13.1% (60/457) Male	No	Fair
	Parenting Stress in Caring for a Chronically Ill Child	Pediatric Inventory for Parents (PIP)									
(Caris et al., 2018) USA	Sibling Adjustment to Illness	Sibling Perception Questionnaire (SPQ)	N/R	HLHS	8.3 ± 7.7 yrs	Siblings and Caregivers	Siblings: 12.5 ± 6.3; Caregiver: 38.7 ± 6.8	32 Siblings; 35 Caregivers	25.7% (9/35) Male	No	Fair
(Chaisom et al., 2010) Thailand	Parenting Stress	Parenting Stress Index-Short Form (PSI-SF; Thai version)	N/R	VSD, ASD, PDA	(1–3 yrs)	Mothers	30.5 (18-45)	95 Mothers	N/A	Yes	Fair

Study				Child with CHD		Parent/Family				Conceptual Framework Identified	Quality Appraisal
(Reference) Study Location	Relevant Construct/s	Relevant Instrument/s	Response Rate	Diagnosis	Age, Mean ± SD (Range)	Respondents	Age, Mean ± SD (Range), Years	CHD Sample Size	% Fathers /Male Caregiver		
(Chang et al., 2020) Taiwan	Parenting Stress	Parenting Stress Index-Short Form (PSI-SF; Chinese version)	63% (716/1133) Participation	CHD with no surgery previous 6 months	6.6 ± 3.8 yrs	Parents	>20	699 Parents	N/R	Yes	Good
(Chien et al., 2021) Taiwan	Anxiety	State-Trait Anxiety Inventory (STAI; Chinese version)	N/R (Convenience Sample)	CHD with previous corrective procedure	23.3 ± 10 months	Parents	Mothers: 34.7 ± 5.4; Fathers: 37.4 ± 5.3	48 Families	N/R	No	Good
(Choi & Lee, 2021) Korea	Coping Self-Efficacy	Coping Self-Efficacy Scale (CSES; revised and translated to Korean for study)	N/R	CHD	1.7 ± 2.9 yrs	Mothers	N/R	100 Mothers	N/A	No	Good
	Parenting Stress	Pediatric Inventory for Parents (PIP; revised and translated to Korean for the study)									
(Cohn, 1996) USA	Reaction to Diagnosis	Study-Specific Instrument	N/R	CHD	N/R	Parents	N/R	N/R	N/R	No	Poor
(Coşkuntürk & Gözen, 2018) Turkey	Anxiety	Beck Anxiety Inventory (Turkish version)	N/R	CHD undergoing cardiac surgery	6–12 yrs	Mothers	Intervention: 36.3 ± 8.5; Control: 37.5 ± 9.2	43 Mothers	N/A	No	Poor
(Davis et al., 1998) USA	Stress Appraisal	Hassles and Uplifts Scale (HSUP)	N/R	CHD	1.7 yrs ± 23 months (9 days–13.6 yrs)	Mothers	28.5 ± 7.9 (16–50)	52 Mothers	N/A	Yes	Good
	Coping	Ways of Coping Questionnaire (WCQ)									

Study				Child with CHD		Parent/Family				Conceptual Framework Identified	Quality Appraisal
**(Reference) Study Location**	**Relevant Construct/s**	**Relevant Instrument/s**	**Response Rate**	**Diagnosis**	**Age, Mean ± SD (Range)**	**Respondents**	**Age, Mean ± SD (Range), Years**	**CHD Sample Size**	**% Fathers /Male Caregiver**		
	Family Functioning	Family Environment Scale (FES)									
	Psychological Adjustment	Brief Symptom Inventory (BSI)									
(Denniss et al., 2019) Australia	Health-Related Quality of Life; Family Functioning	Pediatric Quality of Life (PedsQL) Family Impact Module	49% Participation	CHD	2.7 ± 1.2 yrs	Mothers and Children	34.0 ± 5.8	87 Mothers	N/A	Yes	Good
	Psychological Distress	Depression, Anxiety and Stress Scale-21 Items (DASS-21)									
(De Stasio et al., 2019) Italy	Parenting Stress	Parenting Stress Index-Short Form (PSI-SF)	N/R	CHD	22.6 ± 7.7 months (11–36 months)	Parents	Mothers: 35.4 ± 4.4 (29–50); Fathers: 39.1 ± 5.8 (34–56)	20 Families (40 Parents)	50% (20/40)	No	Poor
(Diffin et al., 2016) Australia	Anxiety; Depression	Hospital Anxiety and Depression Scale (HADS)	67% (37/55 Families; Time 1) 69% (25/37 Families; Time 2); 49/71 parents; Time 3)	CHD requiring neonatal surgery	N/R	Parents	Mothers: 30 ± 5; Fathers: 36 ± 10	71 Parents/ 37 families (Time 1); 51 Parents/26 Families (Time 2); 49 Parents/25 Families (Time 3)	47% (34/71), Time 1	Yes	Fair
	Stress Appraisal	Parent Stressor Scale: Neonatal Intensive Care Unit (PSS:NICU)									
	Coping	Coping Inventory for Stressful Situations (CISS)									
(Doherty et al., 2009)	Psychological Functioning	Brief Symptom Inventory (BSI)	96%	CHD	2.8 ± 1.6 months	Parents	Mothers: 31.5 ± 5.5;		78.6% (55/70)	No	Good

Study				Child with CHD		Parent/Family				Conceptual Framework Identified	Quality Appraisal
(Reference) Study Location	Relevant Construct/s	Relevant Instrument/s	Response Rate	Diagnosis	Age, Mean ± SD (Range)	Respondents	Age, Mean ± SD (Range), Years	CHD Sample Size	% Fathers /Male Caregiver		
Ireland	Coping<br>Worry<br>Family Functioning	COPE Inventory<br>Maternal Worry Scale<br>Family Environment Scale (FES)					Fathers: 33.8 ± 5.4	70 Families (70 Mothers ; 55 Fathers)			
(Dulfer et al., 2015) The Netherlands	Mental Health	General Health Questionnaire-28 (GHQ-28; Dutch version)	17% (56/325)	TOF and Fontan Circulation	Intervention : 13.3 yrs [12.0–15.0], Median [IQR]; Control: 13.2, [11.9–15.3]	Parents	Interventio n: 46.0 [42.0–49.0], Median [IQR] (fathers); 43.5 [40.3–47.0] (mothers); Control: 46.0 [41.8–51.5], (fathers); 43.5 [41.3–46.8] (mothers)	56 Families (baseline) [Interventio n: 34; Control 22] 54 Families (follow up) [Interventio n 34; Control 22]	11 Fathers	No	Fair
(Eagleson et al., 2013) Australia	Health-Related Quality of Life; Family Functioning	Pediatric Quality of Life (PedsQL) Family Impact Module	91% HLV; 85% TOF	HLV; TOF	HLV 5.6 yrs (median) (2.0–16.3); TOF 5.4 yrs (2.6–15.5)	Parents and Children	N/R	60 Parents	6.6% (4/60)	No	Good
(Edraki et al., 2014) Iran	Quality of Life	36-Item Short Form Health Survey (SF-36; Persian version)	87.5% Participation (56/64)	CHD	6 ± 3.3 months	Mothers	28.3 ± 5.6 (CHD group)	28 Parents	N/A	No	Fair

Study				Child with CHD		Parent/Family				Conceptual Framework Identified	Quality Appraisal
(Reference) Study Location	Relevant Construct/s	Relevant Instrument/s	Response Rate	Diagnosis	Age, Mean ± SD (Range)	Respondents	Age, Mean ± SD (Range), Years	CHD Sample Size	% Fathers /Male Caregiver		
(Ezzat et al., 2016) Egypt	Parenting Stress	Parenting Stress Index-Short Form (PSI-SF; Arabic version)	N/R	CHD Conotruncal	15.7 ± 18.1 months	Mothers	28.8 ± 6 (CHD group)	99 Mothers	N/A	No	Fair
(Fischer et al., 2012) USA	Anxiety	State-Trait Anxiety Inventory (STAI)	87% (59/68)	CHD	25.1 ± 22.3 days at discharge	Caregivers	28.2 ± 5.7	59 Caregivers	40.6% (24/59)	No	Good
(Franck et al., 2010) UK	Parent Stress	Parent Stressor Scale: Infant Hospitalization (PSS-IH) (slightly modified)	77% (211/274) Participation	CHD	(<1 months–>10 yrs)	Parents	N/R	Parents of 211 Children (326 Parents Preoperative)	38.0%	No	Good
(Franich-Ray et al., 2013) Australia	Acute Stress Disorder	Acute Stress Disorder Scale (ASDS)	84.3% (97/155), Mothers; 68.6% (79/115), Fathers Participation	CHD post cardiac surgery	<3 months	Parents	32.9 ± 4.9 (19.9–42), Mothers; 35.5 ± 5.5 (23.2–48.9), Fathers	77 Families (77 Mothers; 55 Fathers)	78.6% (55/77)	No	Good
(Garcia et al., 2016) USA	Family Impact	Impact on Family Scale (IOFS; Original version)	98% (100/102)	CHD post cardiac surgery ≥12 months	32 months (median)	Parents	N/R	100 Parents	N/R	No	Good
(Gaskin et al., 2021)	Anxiety	Generalized Anxiety Disorder-7 (GAD-7)	26% (12/47)	Complex CHD post	Infants	Parents	20–40	12 Families (Baseline)		Yes	Fair

Study				Child with CHD		Parent/Family				Conceptual Framework Identified	Quality Appraisal
(Reference) Study Location	Relevant Construct/s	Relevant Instrument/s	Response Rate	Diagnosis	Age, Mean ± SD (Range)	Respondents	Age, Mean ± SD (Range), Years	CHD Sample Size	% Fathers /Male Caregiver		
UK	Depression	Patient Health Questionnaire (PHQ-9)		first stage surgery				(12 Mothers; 4 Fathers); 9 Mothers, 4 Fathers (Time 1); 7 Mothers, 3 Fathers 3 (Time 2); 9 Mothers, 2 Fathers (Time 3)	25% (4/16) at Baseline		
(Goldbeck & Melches, 2006) Germany	Quality of Life	Ulm Quality of Life Inventory for Parents (ULQIE)	88.8% (143/161)	CHD	8.6 ± 6 yrs	Parents and Children	N/R	132 Parents	13.0%	No	Good
(Goldberg et al., 1991) Canada	Parenting Stress	Parenting Stress Index (PSI)	70%	VSD, TGA, TOF, Co-arctation	13.7 ± 1.3 months at follow-up	Parents	Mothers: 27.1 ± 4.6; Fathers: 30.7 ± 6.2	82 Parents	50% (42/82)	No	Fair
(Golfenshtein et al., 2017) USA	Parenting Stress	Parenting Stress Index (PSI)	33% Recruitment (includes controls)	Complex CHD	<6 weeks; Time 1	Parents	N/R	66 Parents	N/R	Yes	Fair
(Guan et al., 2013) China	Psychological Distress	General Health Questionnaire-28 (GHQ-28; Chinese version)	N/R	VSD post surgery and trans-catheter closure	Surgery: 9.1 ± 1.9 (6–13) yrs; Trans-catheter 8.8 ± 1.8 (6–11.5) yrs	Parents	N/R	64 Families and Parents	41 (includes controls)	No	Fair

Study				Child with CHD		Parent/Family				Conceptual Framework Identified	Quality Appraisal
(Reference) Study Location	Relevant Construct/s	Relevant Instrument/s	Response Rate	Diagnosis	Age, Mean ± SD (Range)	Respondents	Age, Mean ± SD (Range), Years	CHD Sample Size	% Fathers /Male Caregiver		
(Hancock et al., 2018) USA	Depression	Beck Depression Inventory-Second Edition (BDI-2)	71% Recruitment	SV undergoing first stage surgery	Prenatal + at time of hospital discharge or 30 days post surgery	Mothers	27.9 ± 5	38 Mothers	N/A	No	Poor
	Anxiety	State-Trait Anxiety Inventory (STAI)									
	Coping	Brief COPE									
	Health-Related Quality of Life; Family Functioning	Pediatric Quality of Life (PedsQL) Family Impact Module									
(Hearps et al., 2014) Australia	Psychosocial Risk	Psychosocial Assessment Tool (PAT 2.0) (modified and extended)	64% (29/48)	CHD and cardiac surgery in <4 weeks of age	(<28–112) days	Caregivers	(20–40+)	39 Caregivers	37.9% (11/29)	Yes	Good
(Helfricht et al., 2008) Switzerland	Post-Traumatic Stress Disorder	Posttraumatic Diagnostic Scale (PDS; German version)	60.9% (139/228), Time 1; 56.1% (128/228) Time 2 Participation	CHD post cardiac bypass surgery	3.2 ± 4.8 yrs age at surgery for Time 1	Parents	N/R	139 Parents (Time 1); 128 Parents (Time 2)	70.5% (98/139), Time 1; 71.0% (92/128), Time 2	No	Good
(Hoehn et al., 2004) USA	Anxiety	State-Trait Anxiety Inventory (STAI)	83% Participation (52/56)	CHD	(~1–10 months) at Phase 1	Parents	N/R	31 Families (30 Mothers; 22 Fathers)	71% (22/31)	No	Fair
	Stress Appraisal	Life Experiences Survey (LES)									
	Optimism	Life Orientation Test (LOT)									

Study				Child with CHD		Parent/Family				Conceptual Framework Identified	Quality Appraisal
(Reference) Study Location	Relevant Construct/s	Relevant Instrument/s	Response Rate	Diagnosis	Age, Mean ± SD (Range)	Respondents	Age, Mean ± SD (Range), Years	CHD Sample Size	% Fathers /Male Caregiver		
(Hunt et al., 2020) Australia	Psychosocial Functioning	Emotional Aspects of Having a Child with CHD Scale	74% (52/71)	CHD pre cardiac surgery	(3 months–10 yrs)	Parents	(20–54)	52 Parents	25% (13/52)	No	Good
(Hussein & Authman, 2013) Iran	Quality of Life	World Health Organization Quality of Life (WHOQOL-100)	N/R	CHD	(1–18 yrs)	Caregivers (Parent/ Sibling/Aunt/ Uncle/Cousin /Grandparent	(16–66)	200	26.5% Male	No	Fair
(Jackson et al., 2020) Australia	Coping Self Efficacy	Coping Self-Efficacy Scale	N/R	CHD	48.6 ± 49.7 months (1.5 months–14 yrs)	Parents	Mothers: 37.7 ± 5.6; Fathers: 36.1 ± 5.2	23 Parents (Baseline) 13 Families including 8 (7 different sex, 1 same sex) Couples	40% (9/23)	Yes	Poor
	Parental Stress	Pediatric Inventory for Parents (PIP)									
	Coping	Coping Scale for Adults, Second Edition-Short Form									
	General Stress	General Stress Item									
(Janus & Goldberg, 1997) Canada	Family Accommodation of Illness	Study-Specific Family Accommodation of Illness Scale	54% (108/200), Initial Response; 49% (53/108) Eligible; 58% (29/53) Participation	CHD	6.6 ± 2.9 yrs	Mothers and Fathers	Mothers: 37.2 ± 4.4; Fathers: 38.7 ± 4.3; Sibling: 7.4 ± 3.3	29 Families (29 Mothers ; 23 Fathers)	43.4% (23/53)	No	Poor
	Sibling Impact	Perception of Effect on Sibling Scale									
(Jordan et al., 2014) Australia	Depression	Edinburgh Postnatal Depression Scale (EPDS)	84.34% (97/115), Recruitment ; 80.41% (78/97) Participation	CHD and surgery <3 months	102.2 ± 42.9 days	Mothers	32.9 ± 4.9	78 Mothers	N/A	No	Fair

Study				Child with CHD		Parent/Family				Conceptual Framework Identified	Quality Appraisal
**(Reference) Study Location**	**Relevant Construct/s**	**Relevant Instrument/s**	**Response Rate**	**Diagnosis**	**Age, Mean ± SD (Range)**	**Respondents**	**Age, Mean ± SD (Range), Years**	**CHD Sample Size**	**% Fathers /Male Caregiver**		
(Kaugars et al., 2018) USA	Parenting Stress	Parenting Stress Index-Short Form (PSI-SF)	N/R	CHD	7.5 ± 2.4 yrs	Parents of Children Referred for Psychological Services	N/R	54 Parents (32 SV; 22 BV)	N/R	No	Good
	Parenting Stress in Caring for a Chronically Ill Child	Pediatric Inventory for Parents (PIP)									
	Parental Health-Related Quality of Life; Family Functioning	Pediatric Quality of Life (PedsQL) Family Impact Module									
(Kiliçarskan-Törüner et al., 2012) Turkey	Anxiety	State-Trait Anxiety Inventory (STAI; Turkish version)	N/R	CHD post cardiac surgery	Intervention: 22.0 ± 19.8 months; Control: 21.3 ± 20.3 months	Parents	Intervention: 29.6 ± 6.0 (Mothers), 33.6 ± 6.0 (Fathers); Control: 27.8 ± 6.1 (Mothers), 31.6 ± 5.7 (Fathers)	93 Families (Intervention: 47; Control: 46)	12.8%, Intervention; 15.2%, Control	No	Poor
(Kumar et al., 2019) India	Anxiety	State-Trait Anxiety Inventory (STAI)	N/R	CHD undergoing cardiac surgery	Intervention: 8.9 ± 2.6 yrs; Control: 8.4 ± 2.5 yrs	Parents	N/R	56 Parents (Intervention: 28; Control: 27)	N/R	No	Poor
	Stress	Index of Clinical Stress									
	Mood	Ottawa Mood Scale									

Study				Child with CHD		Parent/Family				Conceptual Framework Identified	Quality Appraisal
(Reference) Study Location	Relevant Construct/s	Relevant Instrument/s	Response Rate	Diagnosis	Age, Mean ± SD (Range)	Respondents	Age, Mean ± SD (Range), Years	CHD Sample Size	% Fathers /Male Caregiver		
(Ladak et al., 2019) Pakistan	Health-Related Quality of Life	Pediatric Quality of Life (PedsQL) Generic Core Scales (Urdu translation for study)<br>Pediatric Quality of Life (PedsQL) Cognitive Functioning (Urdu translation for study)	42% (252/600)	CHD post cardiac surgery	8.8 ± 3.9 yrs	Siblings	N/R	129 Siblings	N/R Male	Yes	Good
(Landolt et al., 2011) Switzerland	Parental Health-Related Quality of Life<br>Post-Traumatic Stress Symptoms<br>Family Impact	36-Item Short Form Health Survey (SF-36; German version)<br>Posttraumatic Distress Scale (PDS; German version)<br>Impact on Family Scale (IOFS; Revised version)	57.7% (138/239) Enrolment, Time 1; 50% (120/239) Retention, Time 2	CHD post open-heart Surgery	3.1 ± 4.6 yrs (0.1–15.4)	Parents	Mothers: 34.6 ± 5.4; Fathers: 37.6 ± 6.5	138 Families (135 Mothers, 97 Fathers, Time 1); (113 Mothers, 88 Fathers, Time 2)	41.8%, Time 1; 43.78%, Time 2 Fathers	No	Good
(Lawoko & Soares, 2006) Sweden	Distress (Anxiety, Depression, Somatization)<br>Hopelessness	Symptom Checklist-90-Revised (SCL-90-R) (anxiety, depression, and somatization domains only)<br>Beck Hopelessness Scale (BHS)	58% (632/1092)	CHD	8 ± 0.3 (SE) yrs	Parents	41 ± 0.3 (SE)	632 Parents	38.30%	No	Fair

Study				Child with CHD		Parent/Family				Conceptual Framework Identified	Quality Appraisal
(Reference) Study Location	Relevant Construct/s	Relevant Instrument/s	Response Rate	Diagnosis	Age, Mean ± SD (Range)	Respondents	Age, Mean ± SD (Range), Years	CHD Sample Size	% Fathers /Male Caregiver		
(Lee et al., 2020) Canada	Quality of Life	Pediatric Quality of Life (PedsQL) Family Impact Module	N/R	CHD	(1–4 yrs)	Parents	N/R	112 Parents (140 total participants; 28 innocent murmurs)	N/R	No	Good
(Lee et al., 2007) Korea	Parenting Stress	Parenting Stress Index-Short Form (PSI-SF; Korean version)	N/R	CHD	(Newborn–9 yrs)	Mothers	(20–>40)	51 Mothers	N/A	No	Fair
(Levert et al., 2017) The Netherlands	Quality of Life	Linear Analogue Scale	57% (161/282)	CHD prior to invasive cardiac procedure	(0–18 yrs)	Parents	N/R	161 Parents	N/R	No	Good
(Li et al., 2018) China	Parental Distress	Brief Symptom Inventory 18 (BSI-18; Chinese version)	57.6%	CHD	N/R	Parents	Treatment: 32.2 ± 6.2; Control 30.0 ± 6.2	18 Treatment ; 22 Control	38.9%, Treatment ; 36.4%, Control	No	Good
	Hope	Herth Hope Index (HHI; Chinese version)									
(Lisanti, et al., 2021a) USA	Stress Appraisal	Parent Stressor Scale: Infant Hospitalization (PSS:IH)	81% (30/36, Time 1); (23/24, Time 2)	CHD post cardiac surgery	2 ± 1.4 days (Time 1)	Mothers	29.5	30 Mothers	N/A	Yes	Good
	Depression	Center for Epidemiologic Studies Depression Scale (CES-D)									
	Anxiety	State-Trait Anxiety Inventory (STAI)									

Study				Child with CHD		Parent/Family				Conceptual Framework Identified	Quality Appraisal
(Reference) Study Location	Relevant Construct/s	Relevant Instrument/s	Response Rate	Diagnosis	Age, Mean ± SD (Range)	Respondents	Age, Mean ± SD (Range), Years	CHD Sample Size	% Fathers /Male Caregiver		
		Visual Analogue Scale (VAS)									
(Lisanti et al., 2021b) USA	Stress Appraisal	Parent Stressor Scale: Infant Hospitalization (PSS:IH)	47% (28/60)	CHD in PICU post surgery	10 days	Mother-Father Dyads	33.6 ± 4.9	28 Mother-Father Dyads	50% (28/56)	Yes	Fair
	Quality of Partner Relationship	Dyadic Adjustment Scale (DAS)									
	Financial Strain	Financial Strain/ Economic Hardship Instrument									
	Anxiety	State-Trait Anxiety Inventory (STAI)									
	Depression	Centre for Epidemiologic Studies Depression (CES-D)									
(Lisanti et al., 2017) USA	Parental Stress	Parental Stressor Scale: Infant Hospitalization (PSS: IH)	N/R	CHD in PCICU	16.8 ± 9.4 days (4–44)	Mothers	N/R	62 Mothers	N/A	Yes	Good
	Anxiety	State-Trait Anxiety Inventory (STAI)									
(Lopez et al., 2016) Chile	Psychiatric Symptoms	General Health Questionnaire-12 (GHQ-12; Spanish version)	47% (44/94, Time 1); 90% (40/44, Time 2)	CHD	<5 years	Parents	32.2 ± 7.6	40 Parents	65% (26/40)	No	Poor

Study				Child with CHD		Parent/Family				Conceptual Framework Identified	Quality Appraisal
(Reference) Study Location	Relevant Construct/s	Relevant Instrument/s	Response Rate	Diagnosis	Age, Mean ± SD (Range)	Respondents	Age, Mean ± SD (Range), Years	CHD Sample Size	% Fathers /Male Caregiver		
	Hopelessness	Beck Hopelessness Scale (BHS; Spanish version)									
(Majnemer et al., 2006) Canada	Parenting Stress as Indicator of Family Wellbeing	Parenting Stress Index (PSI)	52% (49/94)	CHD	64.2 ± 11.3 months	Parents	N/R	49 Parents	18.2% (9/49)	No	Good
(McCusker et al., 2010) UK	Anxiety	State-Trait Anxiety Inventory (STAI)	95.8% (70/73, Time 1) Recruitment ; 77.14% (54/70, Time 2) Retention	CHD	2.9 ± 1.6 months	Mothers	N/R	54 Mothers	N/A	Yes	Poor
	Maternal Worry in the Context of a Child's Chronic Illness	Maternal Worry Scale									
	Coping	COPE Inventory (4 Subscales of Situational version)									
(McCusker et al., 2012) UK	Maternal Mental Health	Brief Symptom Inventory (BSI)	60.4% (90/149, Time 1) Recruitment ; 75.6% (68/90, Time 2) Retention	CHD	Intervention : 5.5 ± 0.3; Control: 5.4 ± 0.3 yrs	Parents	N/R	90 Families (90, Time; 68, Time 2)	N/R	Yes	Fair
	Maternal Worry in the Context of a Child's Chronic Illness	Maternal Worry Scale									
	Impact of Child's Chronic Illness on the Family	Impact on Family Scale (IOFS; Original version)									

Study				Child with CHD		Parent/Family				Conceptual Framework Identified	Quality Appraisal
(Reference) Study Location	Relevant Construct/s	Relevant Instrument/s	Response Rate	Diagnosis	Age, Mean ± SD (Range)	Respondents	Age, Mean ± SD (Range), Years	CHD Sample Size	% Fathers /Male Caregiver		
(McKechnie et al., 2016) USA	Anxiety<br>Traumatic Stress<br>Depression	State-Trait Anxiety Inventory (STAI)<br>Impact of Event Scale-Revised (IES-R)<br>Center for Epidemiologic Studies Depression Scale (CES-D)	Purposive sampling	CHD	Prenatal (Time 1); Post birth during hospitalization (Time 2)	Parent Couples	Mothers: (23–34); Fathers: (24–35)	6 Couples (12 Parents)	50% (6/12) Fathers	Yes	Poor
(Medoff-Cooper et al., 2020) USA	Parenting Stress<br>Post-Traumatic Stress<br>Quality of Life	Parenting Stress Index (PSI)<br>Posttraumatic Diagnostic Scale (PDS)<br>Ulm Quality of Life Inventory for Parents (ULQIE)	52% Participation (219/343)	CHD post cardiac surgery	Infants	Parents	Intervention: 30 ± 0.5; Control: 30 ± 0.6	219 Parents	3% (6/219)	Yes	Fair
(Menahem et al., 2008) Australia	Anxiety<br>Mental Health<br>Family Functioning	State-Trait Anxiety Inventory (STAI)<br>General Health Questionnaire (GHQ)<br>Family Assessment Device (FAD)	67% (69/103) Recruitment; 56.5% (39/69) Participation	CHD undergoing cardiac surgery	(2.5–12 yrs)	Parents	N/R	39 Families (29 Mothers; 20 Fathers)	25.6% (10/39) Fathers	No	Fair
(Miller et al., 2021) USA	Coping<br>Perceived Stress	Coping Health Inventory for Parents (CHIP)<br>Perceived Stress Scale	40% 21/53	CHD post cardiac surgery	N/R	Parents	Intervention: 28.5 ± 4.7; Control: 30 ± 6.4	21 Parents (11 Intervention; 10 Control)	14% (3/21)	Yes	Poor

Study				Child with CHD		Parent/Family				Conceptual Framework Identified	Quality Appraisal
(Reference) Study Location	Relevant Construct/s	Relevant Instrument/s	Response Rate	Diagnosis	Age, Mean ± SD (Range)	Respondents	Age, Mean ± SD (Range), Years	CHD Sample Size	% Fathers /Male Caregiver		
(Moon et al., 2021) Korea	Sibling Relationship	Sibling Relationship Questionnaire (SRQ)	91% (109/120)	CHD	(13–21 yrs) (72.7% <18 yrs)	Child with CHD	(≤15–≥19)	109 Siblings	56.4% (62/109) Male	No	Good
(Mörelius et al., 2002) Sweden	Parental Stress	Swedish Parenthood Stress Questionnaire (SPSQ)	Complex 81% (26/32); Minor 80% (32/40)	CHD Complex and Minor	(0–9 yrs)	Parents	N/R	101 Parents (45 Complex; 56 Minor)	44.5% (45/101)	No	Fair
(Mussatto et al., 2021) USA/Canada	Family Member Wellbeing/ Adjustment	Family Member Well-Being Index	N/R	HLHS	1.7 ± 3 (Time 1); 6.1 ± 4 months (Time 2); 15.6 ± 5 months (Time 3)	Parents	Mothers: 29 ± 5, Fathers: 32 ± 6 (Time 1); Mothers: 29 ± 6, Fathers: 32 ± 6 (Time 2); Mothers: 31 ± 5, Fathers: 34 ± 7 (Time 3)	154 Parents (Time 1); 117 Parents (Time 2); 89 Parents (Time 3)	32% (50/154, Time 1); 33% (39/117, Time 2); 30% (27/89, Time 3)	Yes	Good
	Family Functioning	Family Assessment Device (FAD: General Functioning Scale)									
	Quality of Life	Perceived Quality of Life Scale (PQOL)									
	Anxiety	State-Trait Anxiety Inventory (STAI)									
	Parenting Stress (Stress Appraisal)	Pediatric Inventory for Parents (PIP)									
	Family Impact	Impact on Family Scale (IOFS; Original version)									
	Family Hardiness	Family Hardiness Index (FHI)									

Study				Child with CHD		Parent/Family				Conceptual Framework Identified	Quality Appraisal
(Reference) Study Location	Relevant Construct/s	Relevant Instrument/s	Response Rate	Diagnosis	Age, Mean ± SD (Range)	Respondents	Age, Mean ± SD (Range), Years	CHD Sample Size	% Fathers /Male Caregiver		
	Coping	Coping Health Inventory for Parents (CHIP)									
	Post-Traumatic Stress	Impact of Event Scale-Revised (IES-R)									
(Pinto et al., 2016) USA	Stress	Brief Symptom Inventory (BSI)	N/R	CHD	Prenatal diagnosis; Postnatal: (birth>4–9 months)	Parents	Mothers: 28.2 (Prenatal), 27.6 (Postnatal); Fathers: 29.9 (Prenatal), 29.2 Postnatal [Mean]	105 Families (60 Prenatal; 45 Postnatal)	48% (98/202)	No	Good
(Poh et al., 2020) Singapore	Parenting Stress	Pediatric Inventory for Parents (PIP)	91% (100/109)	CHD undergoing cardiac surgery	3.7 ± 4.6 yrs	Mothers	35.8 ± 7 (23–56)	100 Mothers	N/A	No	Good
	Coping	Coping Health Inventory for Parents (CHIP)									
(Rahimianfar et al., 2015) Iran	Anxiety	State-Trait Anxiety Inventory (STAI; version unspecified)	N/R	CHD post cardiac surgery	3.8 ± 4.4 yrs (0.8–16 yrs)	Mothers	30.5 ± 8.6 (17–49)	69 Mothers	N/A	No	Good
	Stress Awareness	Study-Specific Instrument									

Study				Child with CHD		Parent/Family				Conceptual Framework Identified	Quality Appraisal
(Reference) Study Location	Relevant Construct/s	Relevant Instrument/s	Response Rate	Diagnosis	Age, Mean ± SD (Range)	Respondents	Age, Mean ± SD (Range), Years	CHD Sample Size	% Fathers /Male Caregiver		
(Re et al., 2018) Australia	Maternal Depression	Edinburgh Postnatal Depression Scale (EPDS)	73.0% (27/37) Recruitment ; 81.5% (22/27) Participation	Serious CHD and surgery in first weeks of life	2.3 months [Median] (2–5 months)	Mothers	31.3 (25–35+)	22 Mothers	N/A	No	Fair
	Anxiety	State-Trait Anxiety Inventory (STAI)									
	Parenting Stress	Parenting Stress Index-Short Form (PSI-SF)									
(Riikonen et al., 2019) Finland	Marital Satisfaction	Evaluating and Nurturing Relationship Issues Communication and Happiness Scale (ENRICH)	N/R	CHD	0–22yrs (>12–<22yrs =7.7%)	Parents	22–53	104 Parents	17.3% (18/104)	No	Fair
(Roberts et al., 2021) Canada	Stress/Coping	Response to Stress Questionnaire (RSQ-CHD)	N/R	CHD	1 yrs 8 months ± 1 yrs	Parents	33.8 ± 4.10	44 Parents	11% (5/44)	No	Good
	Mental Health	Depression, Anxiety and Stress Scale (DASS; depression and anxiety scales only)									
(Rona et al., 1998) UK	Anxiety; Depression	Hospital Anxiety and Depression Scale (HADS)	67.5% (108/160)	CHD (prenatal; postnatal diagnosis)	N/A and N/R	Mothers	≤25–≥36 (confirmed prenatal diagnosis only)	68 Mothers (28 Group A; 40 Group C)	N/A	No	Good
(Rychik et al., 2013)	Post-Traumatic Stress	Impact of Event Scale-Revised (IES-R)	69% (61/88) Recruitment	CHD	Prenatal	Mothers	30 ± 7	59 Mothers	N/A	No	Good

Study				Child with CHD		Parent/Family				Conceptual Framework Identified	Quality Appraisal
(Reference) Study Location	Relevant Construct/s	Relevant Instrument/s	Response Rate	Diagnosis	Age, Mean ± SD (Range)	Respondents	Age, Mean ± SD (Range), Years	CHD Sample Size	% Fathers /Male Caregiver		
USA	Anxiety	State-Trait Anxiety Inventory (STAI)	; 96.72% (59/61) Participation								
	Coping	COPE Inventory									
	Depression	Beck Depression Inventory-Second Edition (BDI-2)									
	Couples/ Partner Adjustment	Dyadic Adjustment Scale (DAS)									
(Sarajuuri et al., 2012) Finland	Parenting Stress	Parenting Stress Index (PSI)	97% HLHS 65% Uni-ventricular	HLHS; Univentricular	18.3 months [Median] (17.2–21.1)	Parents	N/R	23 HLHS 14 Uni-ventricular	65% (24/37) CHD only	No	Good
(Simeone et al., 2018) Italy	Feelings When Carrying Out Activities of Daily Living	12-Item Short-Form Health Survey (SF-12; Italian version)	Purposive Sampling	CHD post cardiac surgery	N/R	Parents	37.8 ± 8.97 (18–49)	24 Parents (18 Mothers; 6 Fathers)	25% (6/24)	No	Poor
(Sira et al., 2014) USA	Coping	Coping Health Inventory for Parents (CHIP; Spiritual Insight and Behavioral Scale – modified)	N/R	CHD	(0–18+)	Mothers	(21–60)	175	N/A	Yes	Poor
(Spijkerboer et al., 2007) The Netherlands	Psychological Distress	General Health Questionnaire-28 (GHQ-28; Dutch version)	69% (109/159)	CHD >7 1/2 yrs post treatment	(7–15 yrs)	Parents	Mothers: 40.5± 4.7; Fathers: 44.5 ± 5.7	161 Parents (109 Families)	37.9% (61/161)	No	Good
	Coping	Utrecht Coping List (UCL)									

Study				Child with CHD		Parent/Family				Conceptual Framework Identified	Quality Appraisal
(Reference) Study Location	Relevant Construct/s	Relevant Instrument/s	Response Rate	Diagnosis	Age, Mean ± SD (Range)	Respondents	Age, Mean ± SD (Range), Years	CHD Sample Size	% Fathers /Male Caregiver		
(Stoffel et al., 2017) Switzerland	Health-Related Quality of Life<br><br>Psychosocial Impact on Family Living	36-Item Short Form Health Survey (SF-36; German version)<br><br>Impact on Family Scale (IOFS; German version)	N/R	HLHS/ Uni-ventricular	Infants	Parents	Mothers: 29 [Median] (20–39); Fathers: 32.5 (22–48)	19 Parents (10 Families)	45% (9/19)	No	Good
(Svavarsdottir & McCubbin, 1996) USA	Family Demands<br><br>Coping<br><br>Caregiver Burden	Family Inventory of Life Events and Changes (FILE)<br><br>Coping Health Inventory for Parents (CHIP)<br><br>The Care of My Child – CHD modification of Caregiving Burden Scale	N/R	CHD	4 months 3 weeks ± 3 months (1 month–1 yrs)	Parents	Mothers: 28.7 [Mean]	142 Parents (71 Families)	50% (71/142)	No	Good
(Tallon et al., 2015) Australia	Depression, Anxiety, Stress	Depression, Anxiety and Stress Scale (DASS)	61% (91/148)	CHD at time of Cardiac Surgery	32 months (<1–>3yrs)	Mothers	19–54	91 Mothers	N/A	Yes	Good
(Torowicz et al., 2010) USA	Parental Stress	Parenting Stress Index (PSI)	N/R	Complex CHD requiring intervention within first 6 weeks of life	3 months	Mothers	N/R	129 Mothers	N/A	No	Poor

Study (Reference) Study Location	Relevant Construct/s	Relevant Instrument/s	Response Rate	Child with CHD: Diagnosis	Child with CHD: Age, Mean ± SD (Range)	Parent/Family: Respondents	Parent/Family: Age, Mean ± SD (Range), Years	Parent/Family: CHD Sample Size	Parent/Family: % Fathers /Male Caregiver	Conceptual Framework Identified	Quality Appraisal
(Uhm & Kim, 2019) Korea	Anxiety	State-Trait Anxiety Inventory (STAI; Korean version)	N/R	CHD and cardiac surgery in first 3 months	Intervention: 19.9 ± 22; Control: 29.2 ± 25.1 days	Mothers	Intervention: 33 ± 3.8; Control: 32.9 ± 4.7	73 Mothers	N/A	Yes	Fair
(Utens et al., 2000) The Netherlands	Psychological Distress	General Health Questionnaire-28 (GHQ-28; Dutch version)	73% (Surgery); 74% (Intervention)	CHD prior to cardiac surgery or intervention	(3 months–6 yrs)	Parents	Mothers: 31.5 ± 5.5 (Surgery), 32.2 ± 4.1 (Intervention); Fathers: 34.3 ± 4.9 (Surgery), 34.7 ± 4.4 (Intervention)	186 Parents (Mothers: 75 [Surgery], 19 [Intervention]; Fathers: 75 [Surgery], 17 [Intervention]	49.4% (92/186)	No	Good
	Coping	Utrecht Coping List (UCL)									
(Uzark & Jones, 2003) USA	Parenting Stress	Parenting Stress Index-Short Form (PSI-SF)	N/R	CHD	6.3 ± 3.1 (2.1–12.6) yrs	Parents	N/R	80 Parents	12.5% (10/80) Fathers	Yes	Good
(Üzger et al., 2015) Turkey	Anxiety	Beck Depression Inventory (Turkish version)	N/R	CHD undergoing angio-graphy	61.9 ± 50.7 (5–216) months	Parents	Mothers: 31.8 ± 7.8 (20–54); Fathers: 36.1 ± 8.5 (22–59)	73 Families (73 Mothers; 73 Fathers)	100% (73/73)	No	Fair
	Depression	Beck Anxiety Inventory (Turkish version)									
(van der Mheen et al., 2019) The Netherlands	Mental Health	Symptom Checklist-90-Revised (SCL-90-R; Dutch version)	92% (45/49, Intervention); 100%	CHD	Intervention: 5.4 ± 1.3; Control: 5.2	Parents	N/R	87 Families (47 Intervention	44% (44/7)	Yes	Fair

Study				Child with CHD		Parent/Family				Conceptual Framework Identified	Quality Appraisal
(Reference) Study Location	Relevant Construct/s	Relevant Instrument/s	Response Rate	Diagnosis	Age, Mean ± SD (Range)	Respondents	Age, Mean ± SD (Range), Years	CHD Sample Size	% Fathers /Male Caregiver		
	Parental Worry	Penn State Worry Questionnaire (PSWQ; Dutch version)	(40/40, Control) Retention		± 1.7 yrs (at baseline)			; 40 Control at baseline)			
	Parenting Stress	Distress Thermometer (Dutch version)									
	Health-Related Quality of Life	36-Item Short Form Health Survey (SF-36; Dutch version)									
	Family Functioning	Family Assessment Device (FAD)									
(Visconti et al., 2002) USA	Parental Stress	Parenting Stress Index (PSI)	97% (158/163) Retention	TGA	1 yrs (Time 1); 4 yrs (Time 2) [Mean]	Parents	N/R	143 (Time 1); 153 (Time 2) Families	N/R	No	Good
(Jantien Vrijmoet-Wiersma et al., 2009) The Netherlands	Psychological Distress	General Health Questionnaire-12 (GHQ-12; Dutch version)	81.05% (231/285) Recruitment ; 68.7% (196/285) Participation	CHD	45.3 ± 20.6 (10–97) months	Parents	35.9 ± 4.5 (25–52)	196 Parents (131 Families)	41.8% (82/196) Fathers	No	Good
	Anxiety	State-Trait Anxiety Inventory (STAI; version unspecified)									
	Parenting Stress	Parenting Stress Index-Short Form (PSI-SF; Dutch version)									
	Illness-Related Parental Stress	Pediatric Inventory for Parents-Short Form (PIP-Short									

Study				Child with CHD		Parent/Family				Conceptual Framework Identified	Quality Appraisal
(Reference) Study Location	Relevant Construct/s	Relevant Instrument/s	Response Rate	Diagnosis	Age, Mean ± SD (Range)	Respondents	Age, Mean ± SD (Range), Years	CHD Sample Size	% Fathers /Male Caregiver		
		Form; Developed for study by authors; Dutch version)									
(Warnakulasooriya & Kasturiaratchi, 2020) Sri Lanka	Quality of Life	World Health Organization Quality of Life-BREF (WHOQOL-BREF; Sri Lankan version)	99% (422/426)	CHD	N/R	Caregivers	(<20–59)	422 Parents	9.5% (40/422)	No	Good
(Werner et al., 2019) France	Anxiety	Visual Analogue Scale (VAS)	36% (73/205) Participation	CHD before invasive procedure	11 months [Median] (7 days–13 years)	Parents	N/R	120 Parents (73 families)	40.8% (49/120) Fathers	No	Good
(Yildiz et al., 2009) Turkey	Psychological Distress	Symptom Checklist-90-Revised (SCL-90-R; Depression, Anxiety and Somatization subscales)	N/R	CHD	(3 months–7 yrs)	Parents	(20–40+)	262 Parents (132 Mothers; 130 Fathers; 142 Families)	49.6% (130/262) Fathers	No	Good
(Zhang et al., 2021) China	Quality of Life	World Health Organization Quality of Life-BREF (WHOQOL-BREF)	84% (70/83) Recruitment ; 93% (65/70) Retention	VSD	Intervention : 18 ± 8.4; Control: 16.2 ± 7.3 days	Parents	(<25–>40)	65 Parents (35 Intervention; 30 Control)	N/R	No	Fair
	Anxiety	Self-Rating Anxiety Scale (SAS)									
(Zhang et al., 2020) China	Caregiver Burden	(Family) Caregiver Task Inventory (Chinese version); Zarit Caregiver Burden Interview (ZBI)	90% (72/80) Retention	ASD	Intervention : 22.6 ± 16.5; Control: 16.2 ± 7.3 days	Parents	(<25–>40)	80 Parents (Baseline)	N/R	No	Fair

Note: N/A – Not Applicable; N/R – Not Reported.

Figure S4: Frequency of instrument usage across studies.

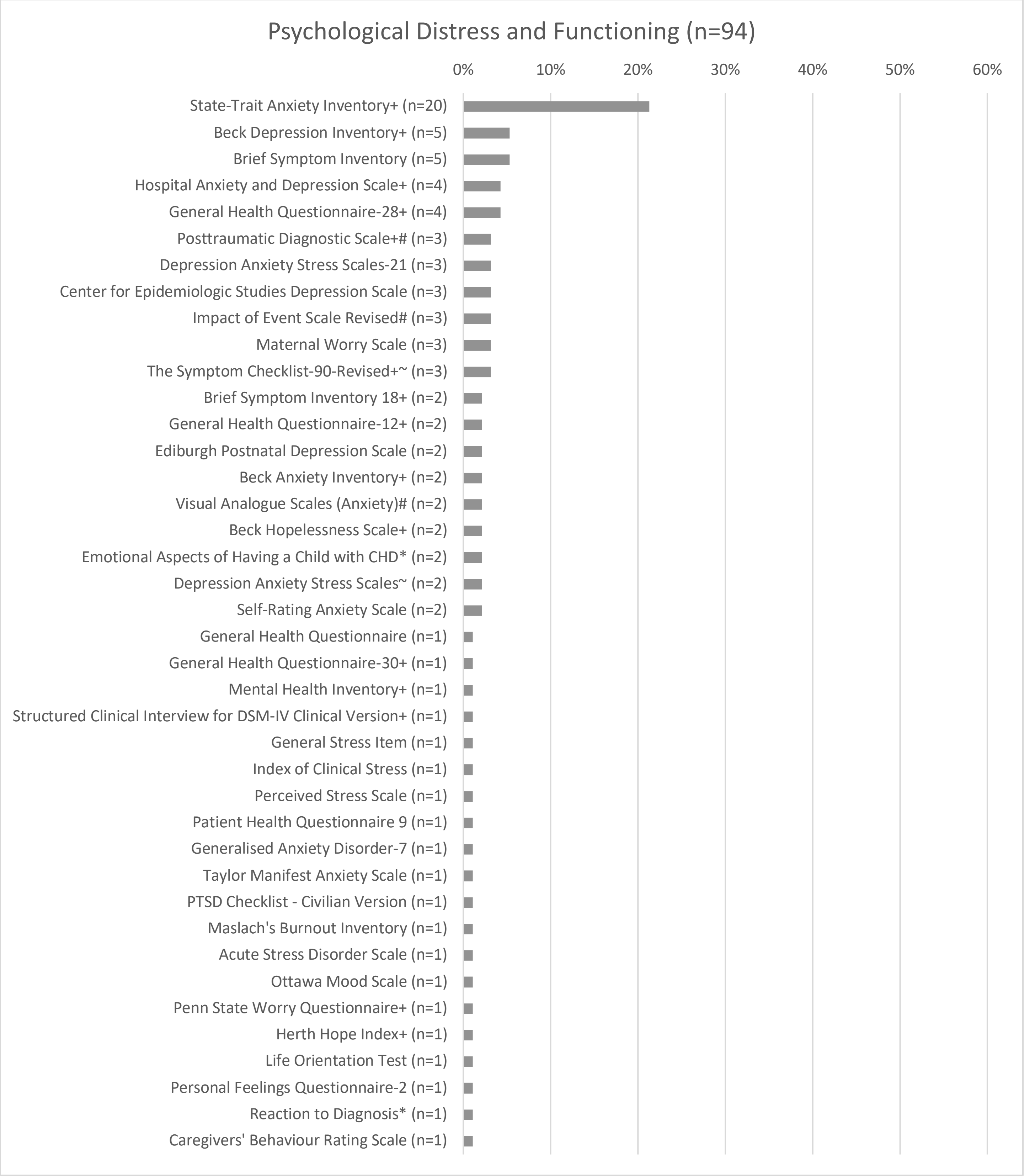

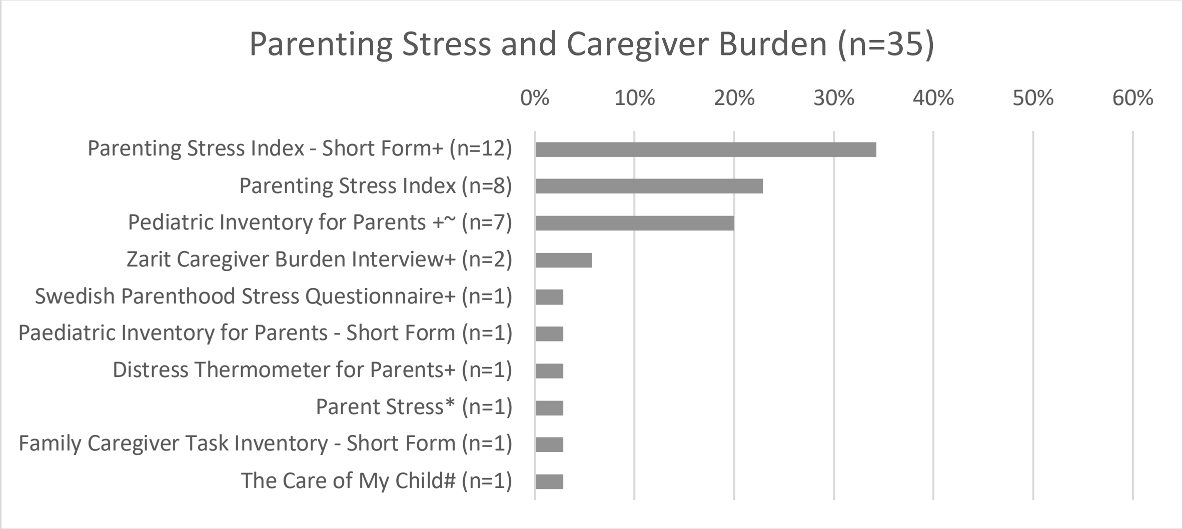
Parenting Stress and Caregiver Burden (n=35)
0%
10%
20%
30%
40%
50%
60%
Parenting Stress Index - Short Form+ (n=12)
Parenting Stress Index (n=8)
Pediatric Inventory for Parents +~ (n=7)
Zarit Caregiver Burden Interview+ (n=2)
Swedish Parenthood Stress Questionnaire+ (n=1)
Paediatric Inventory for Parents - Short Form (n=1)
Distress Thermometer for Parents+ (n=1)
Parent Stress* (n=1)
Family Caregiver Task Inventory - Short Form (n=1)
The Care of My Child# (n=1)

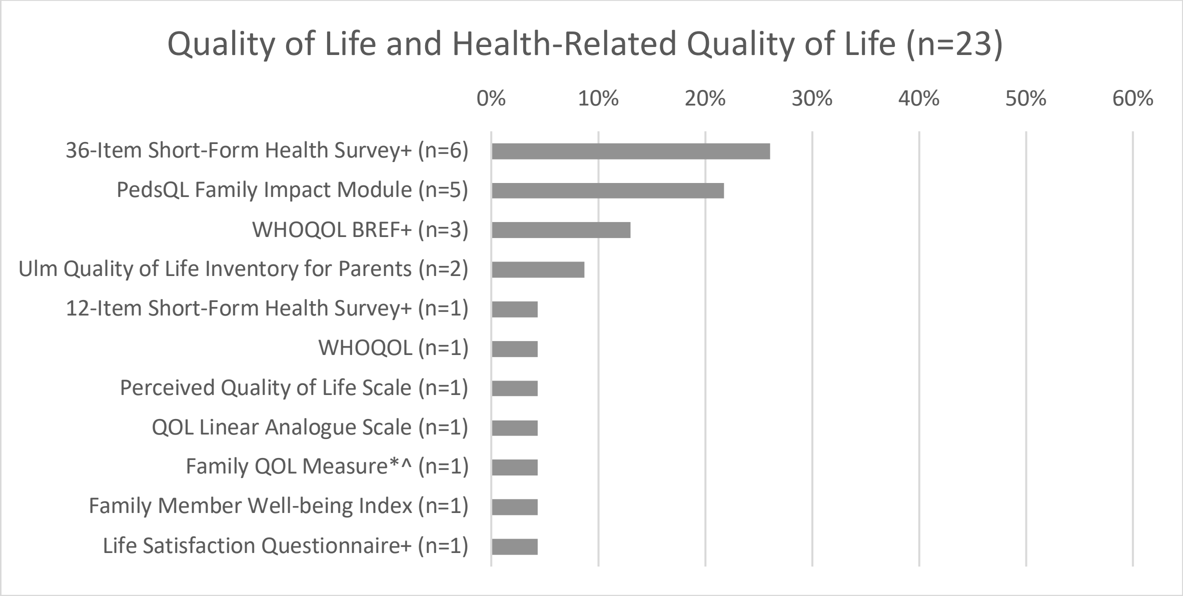
Quality of Life and Health-Related Quality of Life (n=23)
0%
10%
20%
30%
40%
50%
60%
36-Item Short-Form Health Survey+ (n=6)
PedsQL Family Impact Module (n=5)
WHOQOL BREF+ (n=3)
Ulm Quality of Life Inventory for Parents (n=2)
12-Item Short-Form Health Survey+ (n=1)
WHOQOL (n=1)
Perceived Quality of Life Scale (n=1)
QOL Linear Analogue Scale (n=1)
Family QOL Measure*^ (n=1)
Family Member Well-being Index (n=1)
Life Satisfaction Questionnaire+ (n=1)

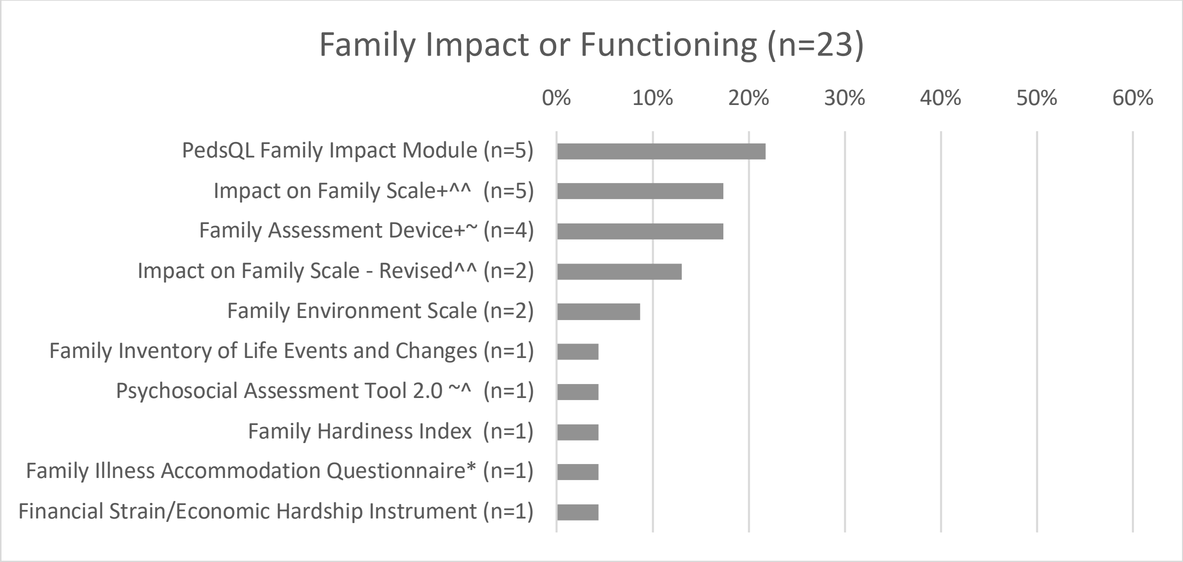
Family Impact or Functioning (n=23)
0%
10%
20%
30%
40%
50%
60%
PedsQL Family Impact Module (n=5)
Impact on Family Scale+^^ (n=5)
Family Assessment Device+~ (n=4)
Impact on Family Scale - Revised^^ (n=2)
Family Environment Scale (n=2)
Family Inventory of Life Events and Changes (n=1)
Psychosocial Assessment Tool 2.0 ~^ (n=1)
Family Hardiness Index (n=1)
Family Illness Accommodation Questionnaire* (n=1)
Financial Strain/Economic Hardship Instrument (n=1)

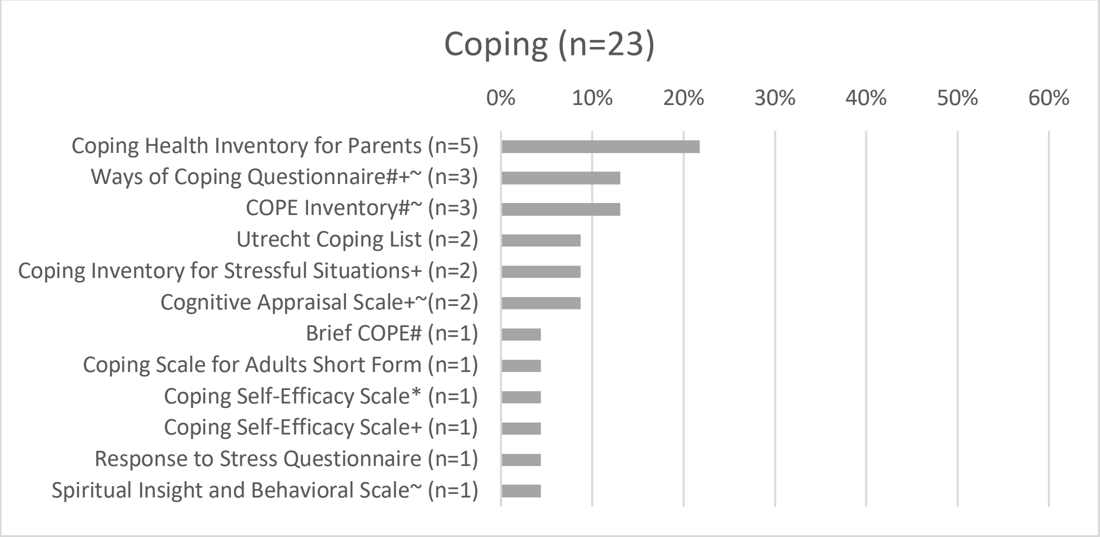

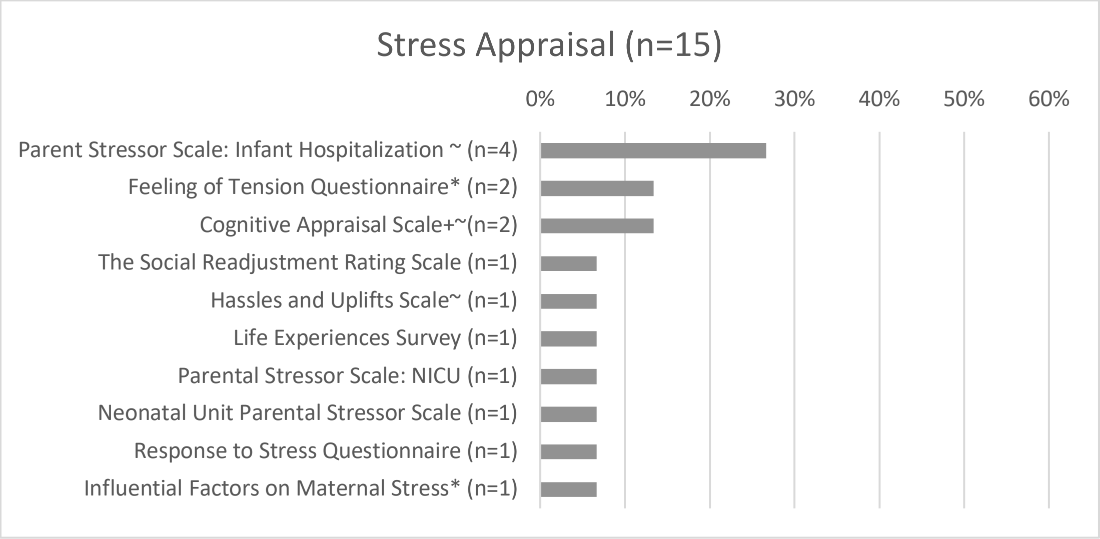

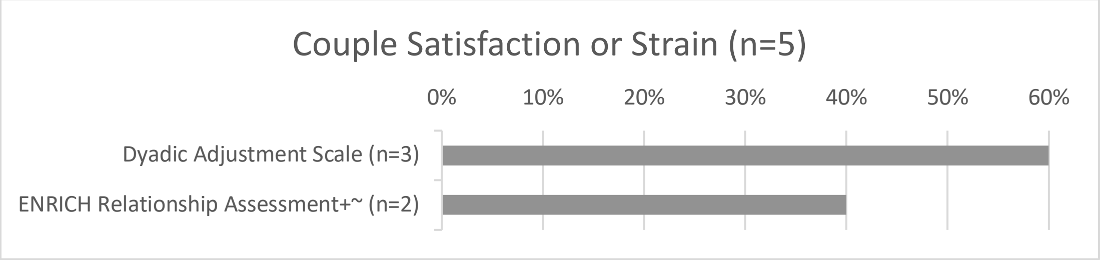

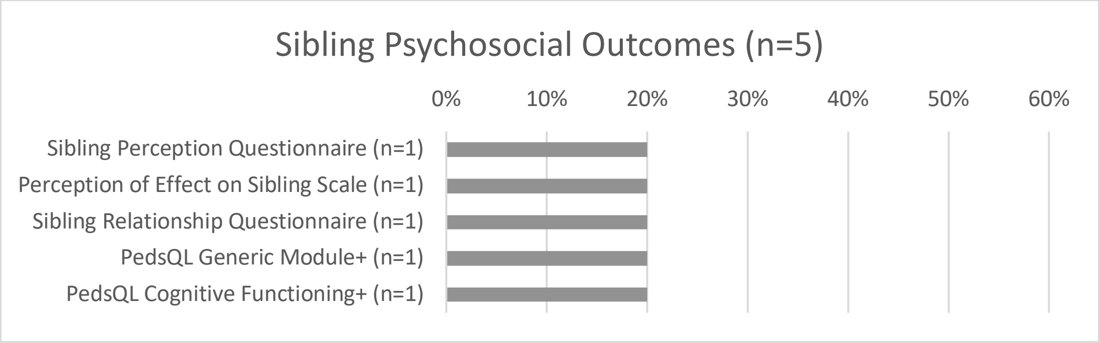

Legend:

+ Includes translated version/s

*Study-specific measure

#Designed to be adapted to context

~ Includes subscale/s use only/modified for study

^Optional sibling subscale

^^Includes sibling subscale

Table S5: Instrument characteristics.

Instrument	Type	Construct	Domains or Scales or Indices	Number of Items / Response Scale	Timeframe of Response	Administration Method**	Time to Complete, Minutes	Scoring	Licensing or Cost	Minimum Available Languages
**Psychological Functioning and Distress**										
**Symptom Checklist-90-Revised (SCL-90-R)** (Derogatis et al., 1976; Derogatis & Savitz, 1999) *English, Turkish, Dutch versions	Generic	Psychological Problems; Psychopathology Symptoms	Symptom Scales: Somatization; Obsessive-Compulsive; Interpersonal Sensitivity; Depression; Anxiety; Hostility; Phobic Anxiety; Paranoid Ideation; Psychoticism<br><br>Global Indices: General Severity Index; Positive Symptom Distress Index; Positive Symptom Total	90 / 5-Point Scale	Past Week	Self-Report<br>Paper and Pencil; Computer; Online	12–15	Manual; Software; Web based; Mail in	Yes	English + >20 Languages
**Brief Symptom Inventory (BSI)** (Derogatis & Melisaratos, 1983)	Generic	Psychological Problems; Psychopathology Symptoms	Symptom Scales: Somatization; Obsessive-Compulsive; Interpersonal Sensitivity; Depression; Anxiety; Hostility; Phobic Anxiety; Paranoid Ideation; Psychoticism<br><br>Global Indices: General Severity Index; Positive Symptom Distress Index; Positive Symptom Total	53 / 5-Point Scale	Past 7 Days	Self-Report<br>Paper and Pencil; Computer	8–10	Manual; Software; Web based; Mail in	Yes	English, French for Canada, Spanish

Instrument	Type	Construct	Domains or Scales or Indices	Number of Items / Response Scale	Timeframe of Response	Administration Method**	Time to Complete, Minutes	Scoring	Licensing or Cost	Minimum Available Languages
**Brief Symptom Inventory 18 (BSI-18)** (Derogatis, 2001) *English, Chinese versions	Generic	Psychological Problems; Psychopathology Symptoms	Symptom Scales: Somatization; Depression; Anxiety<br><br>Global Severity Index	18 / 5-Point Scale	Past 7 Days	Self-Report<br>Paper and Pencil; Computer; Online	4	Manual; Software; Web based	Yes	English and Spanish
**General Health Questionnaire (GHQ)** (Goldberg & Blackwell, 1970)	Generic	Minor Psychiatric Disorders	Total Score	60 / 4-Point Scale	Present State	Self-Report<br>Paper and Pencil	6-8	Manual	Yes	English + 3 Languages
**GHQ-30** (Fontanesi et al., 1985) Italian version	Generic	Minor Psychiatric Disorders	Total Score	30 / 4-Point Scale	Present State	Self-Report<br>Paper and Pencil	3-4	Manual	Yes	English + >5 Languages
**GHQ-28** *Dutch, Chinese versions	Generic	Minor Psychiatric Disorders	Somatic Symptoms; Anxiety/Insomnia; Social Dysfunction; Severe Depression	28 / 4-Point Scale	Present State	Self-Report<br>Paper and Pencil	~7	Manual	Yes	English + >20 Languages
**GHQ-12** Dutch, Spanish versions	Generic	Minor Psychiatric Disorders	Total Score	12 / 4-Point Scale	Present State	Self-Report<br>Paper and Pencil; e-version	~2	Manual	Yes	English + >40 Languages
**Mental Health Inventory (MHI)** (Veit & Ware, 1983) Hebrew version	Generic	Psychological Distress; Psychological Wellbeing	Psychological Distress; Psychological Wellbeing; General Positive Affect; Emotional Ties; Life Satisfaction; Anxiety; Depression; Loss of Behavioral/Emotional Control; global Mental Health Index score	38 / 6-Point Scale	Past Month	Self-Report<br>Paper and Pencil	~10	Manual	Yes	English, Hebrew +

Instrument	Type	Construct	Domains or Scales or Indices	Number of Items / Response Scale	Timeframe of Response	Administration Method**	Time to Complete, Minutes	Scoring	Licensing or Cost	Minimum Available Languages
**Structured Clinical Interview for DSM-IV Clinical version (SCID 1)** (El Missiry et al., 2003) Arabic version	Generic	Psychiatric Disorders	N/I	N/I	N/I	N/I	N/I	N/I	N/I	English, Arabic +
**Perceived Stress Scale (PSS-10)** (Cohen & Williamson, 1988)	Generic	General Life Stress	Total Score	10 / 5-Point Scale	Past Month	Self-Report<br>Paper and Pencil	5–10	Manual	No	English + >10 Languages
**General Stress Item** (Elo et al., 2003)	Generic	General Stress	Total Score	1 / 5-Point Scale	Present	Self-Report<br>Paper and Pencil	~1	Manual	No	English +
**Index of Clinical Stress** (Abell, 1991)	Generic	Stress	Total Score	25 / 5-Point Scale	Present	Self-Report<br>Paper and Pencil; Online	~7	Manual; Online	Yes	English
**Depression Anxiety and Stress Scale (DASS)** (Lovibond & Lovibond, 1995)	Generic	Depression; Anxiety; Stress	Depression; Anxiety; Stress	42 / 4-Point Scale	Past Week	Self-Report<br>Paper and Pencil	~11	Manual	No	English + 50 Languages
**Depression Anxiety and Stress Scale-21 Items (DASS-21)** (Lovibond & Lovibond, 1995)	Generic	Depression; Anxiety; Stress	Depression; Anxiety; Stress	21 / 4-Point Scale	Past Week	Self-Report<br>Paper and Pencil	~6	Manual	No	English + 50 Languages

Instrument	Type	Construct	Domains or Scales or Indices	Number of Items / Response Scale	Timeframe of Response	Administration Method**	Time to Complete, Minutes	Scoring	Licensing or Cost	Minimum Available Languages
**Hospital Anxiety and Depression Scale (HADS)** (Zigmond & Snaith, 1983) *English, Turkish, Swedish versions	Illness-focused	Anxiety; Depression	Anxiety; Depression	14 / 5-Point Scale	Past Week	Self-Report<br>Paper and Pencil	2–5	Manual	Yes	English + >100 Languages
**Beck Depression Inventory-Second Edition (BDI-2)** (Beck, 1996) *English, Italian, Arabic, Turkish versions	Generic	Depression	Total Score	21 / 4-Point Scale	2 Weeks	Self-Report<br>Paper and Pencil	5	Manual; Online	Yes	English+
**Center for Epidemiologic Studies Depression Scale (CES-D)** (Radloff, 1977)	Generic	Depression	Total Score	20 / 4-Point Scale	Past Week	Self-Report<br>Paper and Pencil	~5	Manual	No	English
**Patient Health Questionnaire (PHQ-9)** (Kroenke & Spitzer, 2002; Kroenke et al., 2001)	Generic	Depression	Total Score	9 / 4-Point Scale	Past 2 Weeks	Self-Report<br>Paper and Pencil	~3	Manual	No	English + >30 languages

Instrument	Type	Construct	Domains or Scales or Indices	Number of Items / Response Scale	Timeframe of Response	Administration Method**	Time to Complete, Minutes	Scoring	Licensing or Cost	Minimum Available Languages
**Edinburgh Postnatal Depression Scale (EPDS)** (Cox et al., 1987)	Generic	Postnatal Depression	Nil	10 / 4-Point Scale	Past Week	Self-Report<br>Paper and Pencil; Online	~3	Manual; Online	No	English + >30 Languages
**State-Trait Anxiety Inventory (STAI)** (Spielberger et al., 1983) *English, Dutch, Chinese, Turkish, Korean versions	Generic	Anxiety	State Anxiety; Trait Anxiety	40 / 4-Point Scale	Present	Self-Report<br>Paper and Pencil; Online	10	Manual; Online	Yes	English, Dutch + >50 Languages
**Beck Anxiety Inventory (BAI)** (Ulusoy et al., 1998) *Turkish version	Generic	Anxiety	Total Score	21 / 4-Point Scale	Past Week	Self-Report<br>Paper and Pencil; Computer; Online	5–10	Manual; Online	No	English, Spanish, Turkish +
**Generalized Anxiety Disorder-7 (GAD-7)** (Spitzer et al., 2006)	Generic	Anxiety	Total Score	7 / 4-Point Scale	Past 2 Weeks	Self-Report<br>Paper and Pencil	~2	Manual	N/I	English + >80 Languages
**Self-Rating Anxiety Scale (SAS)** (Zung, 1971)	Generic	Anxiety	Index Score	20 / 4-Point Scale	Past Several Days	Self-Report<br>Paper and Pencil	~5	Manual	N/I	English

Instrument	Type	Construct	Domains or Scales or Indices	Number of Items / Response Scale	Timeframe of Response	Administration Method**	Time to Complete, Minutes	Scoring	Licensing or Cost	Minimum Available Languages
**Visual Analogue Scales (VAS)** (Hornblow & Kidson, 1976)	Adaptable to Specific Context	Anxiety (can be adapted to other contexts)	Total Score	1 / 10 and 1/100mm Line Scale	Present	Self-Report<br>Paper and Pencil	1	Manual	No	English +
**Taylor Manifest Anxiety Scale (TMAS)** (Taylor, 1951, 1953)	Generic	Anxiety	Total Score	50 / True/False Response	Present	Self-Report<br>Paper and Pencil	~10	Manual	N/I	English
**Impact of Event Scale Revised (IES-R)** (Weiss, 2007; Weiss & Charles, 1997)	Adaptable to Specific Context	Symptomatic Response to Specific Traumatic Stressor	Intrusion; Avoidance; Hyperarousal; Total Score	22 / 5-point Scale	Past 7 Days	Self-Report<br>Paper and Pencil	~6	Manual	No	English + > 5 Languages
**PTSD Checklist-Civilian Version (PCL-C)** (Weathers et al., 1993)	Generic	Post-Traumatic Stress	Symptom Scales: Intrusive Thoughts; Avoidance of Stimuli Related to Traumatic Event; Hypervigilance; Total Score	17 / 5-Point Scale	Past Month	Self-Report<br>Paper and Pencil	~5	Manual	N/I	English, Greek, Spanish
**Posttraumatic Diagnostic Scale (PDS)** (Foa et al., 1997) *English, German versions	Adaptable to Specific Context	Post-Traumatic Stress	Reexperiencing; Avoidance; Arousal; Total Symptom Severity	49 / 4-Point Scale	Past Month	Self-Report<br>Paper and Pencil	10–15	Manual	Yes	English + 5 Languages
**Acute Stress Disorder Scale (ASDS)** (Bryant et al., 2000)	Generic	Acute Post-Traumatic Stress	Dissociation; Reexperiencing; Avoidance; Arousal; Total Score	19 / 5-point scale	Present	Self-Report<br>Paper and Pencil	~5	Manual	N/I	English

Instrument	Type	Construct	Domains or Scales or Indices	Number of Items / Response Scale	Timeframe of Response	Administration Method**	Time to Complete, Minutes	Scoring	Licensing or Cost	Minimum Available Languages
**Maslach's Burnout Inventory (MBI)** (Maslach et al., 1996)	Generic	Burnout	Emotional Exhaustion; Depersonalization; Personal Accomplishment	22 / 7-Point Scale	Frequency Likert Scale	Self-Report<br>Paper and Pencil; Online	10–15	Manual; Online	Yes	English + >40 Languages
**Ottawa Mood Scale** (Cheng, 2011)	Generic	Mood	Arousal/Self-regulation; Mood; Anger; Worry; Stress	5 / 3, 6, 11-Point Scale	Present	Self-Report<br>Paper and Pencil	~2	Manual	No	English
**Penn State Worry Questionnaire (PSWQ)** (van Rijsoort et al., 1999) Dutch version	Generic	Worry	Total Score	16 / 5-Point Scale	Present	Self-Report<br>Paper and Pencil	~4	Manual	N/I	English + Multiple Languages
**Maternal Worry Scale (MWS)** (DeVet & Ireys, 1998)	Generic	Worry	Total Score	11 / 4-Point Scale	Present	Self-Report<br>Paper and Pencil	~3	Manual	N/I	English
**Beck Hopelessness Scale (BHS)** (Beck et al., 1975) *English, Spanish versions	Generic	Hopelessness	Total Score	20 / True/False Response	Present	Self-Report<br>Paper and Pencil	10	Manual	Yes	English Spanish +
**Herth Hope Index (HHI)** (Chan et al., 2012) Chinese version	Generic	Hope	Inner Sense of Temporality and Future; Inner Positive Readiness and Expectancy; Inter-Connectedness with self and Others	12 / 4-Point Scale	Present	Self-Report<br>Paper and Pencil	~3	Manual	N/I	English, Chinese +

Instrument	Type	Construct	Domains or Scales or Indices	Number of Items / Response Scale	Timeframe of Response	Administration Method**	Time to Complete, Minutes	Scoring	Licensing or Cost	Minimum Available Languages
**Life Orientation Test (LOT)** (Scheier & Carver, 1985)	Generic	Optimism	Total Score	12 / 5-Point scale	Present	Paper and pencil	~ 3	Manual	N/I	English
**Emotional Aspects of Having a Child with CHD** (Blue et al., 2015)	Study-Specific	Emotional Aspects of Having a Child with CHD	Total Score	7 / Yes/No/Maybe Responses	Present	Self-Report<br>Paper and Pencil	~2	Manual	N/A	English
**Personal Feelings Questionnaire-2 (PFQ-2)** (Harder & Zalrna, 1990)	Generic	Shame; Guilt	Shame; Guilt	16 / 5-Point Scale	Frequency Likert Scale	Self-Report<br>Paper and Pencil	~4	Manual	N/I	English
**Reaction to Diagnosis, Study-Specific Instrument** (Cohn, 1996)	Study-Specific	Reaction to Diagnosis	Feelings Items	5 / 5-Point Scale	Past 6 Weeks	Self-Report<br>Paper and Pencil	~2	Manual	N/I	English
**Caregivers' Behavior Rating Scale (CBRS)** (Campbell et al., 1992)	Illness-Focused	Behavior at Stress Point	Behavior/Distress	1 / 5-Point Scale	Present	Clinician Observed/ Report<br>Paper and Pencil	~1	Manual	N/I	English
**Parenting Stress and Caregiver Burden**										
**Parenting Stress Index 3rd Edition** (Abidin, 2012) English (4th Ed)	Generic	Parenting Stress	Child Domain; Parent Domain; Total Stress	101 / 5-Point Scale + optional 19 item Life Stress Scale	Present	Self-Report<br>Paper and Pencil; Online	20	Manual; Software	Yes	English + >25 languages

Instrument	Type	Construct	Domains or Scales or Indices	Number of Items / Response Scale	Timeframe of Response	Administration Method**	Time to Complete, Minutes	Scoring	Licensing or Cost	Minimum Available Languages
**Parenting Stress Index-Short Form (PSI-SF/*PSI-4-SF*)** (Abidin, 2012) *English, Dutch, Thai, Arabic, Chinese versions; Korean translation	Generic	Parenting Stress	Parental Distress; Parent-Child Dysfunctional Interaction; Difficult Child; Total Stress	36 / 5-Point Scale	Present	Self-Report<br>Paper and Pencil; Online	10	Manual; Software	Yes	English + >10 Languages
**Swedish Parenthood Stress Questionnaire (SPSQ)** (Östberg et al., 1997)	Generic	Parenting Stress	Incompetence; Role Restriction; Social Isolation; Spouse Relationship Problems; Health Problems; Total Score	34 / 5-Point Scale	Present	Self-Report<br>Paper and Pencil	~11	Manual	N/I	Swedish
**Pediatric Inventory for Parents (PIP)** (Streisand et al., 2001)	Illness-Focused	Parenting Stress	Communication; Medical Care; Role Function; Emotional Function; Frequency Total; Difficulty Total; PIP Total	42 Answered Twice for Frequency and Difficulty / 5-Point Scale	Past Week	Self-Report<br>Paper and Pencil	~11	Manual	No	English, Dutch, Swedish
**Pediatric Inventory for Parents (PIP) - Short Form** (Jantien Vrijmoet-Wiersma et al., 2010) Dutch version	Illness-Focused		Communication; Medical Care; Role Function; Emotional Function; Total Score	14 Answered Twice for Frequency and Difficulty / 5-Point Scale	Past Week	Self-Report<br>Paper and Pencil	~4	Manual	N/I	Dutch

Instrument	Type	Construct	Domains or Scales or Indices	Number of Items / Response Scale	Timeframe of Response	Administration Method**	Time to Complete, Minutes	Scoring	Licensing or Cost	Minimum Available Languages
**Distress Thermometer for Parents (DT-P)** (Haverman et al., 2013)	Illness-Focused	Distress	Thermometer Score (Overall distress) Practical Problems; Social Problems; Emotional; Physical; Cognitive; Parenting Problems <2years; ≥ 2 years; Total Score (5 or 6 domains); Additional questions - Enough support + Need for referral	43 (<2years); 41 (≥ 2 Years) / 10-Point Scale + Yes/No Response	Past Week	Self-Report<br>Paper and Pencil	~11	Manual	N/I	Dutch
**Parent Stress, Study-Specific Instrument** (Barsella et al., 2021)	Study-Specific	Parental Stress in Caregiving Role	Parent stress items	14 (4 Stress Items) / 5-Point Scale	Present	Self-Report<br>Paper and Pencil	~1 (stress items only)	Manual	N/I	English
**Caregiver Task Inventory** (Lee & Mok, 2011) Short Form Chinese version	Illness-Focused	Caregiver Burden	Learning to Cope with New Role; Providing Care According to Care-Receiver's Needs; Managing Own Emotional Needs; Appraising Supportive Resources; Balancing Caregiving Needs and Own Needs	25 / 3-Point Scale	Present	Self-Report<br>Paper and Pencil	~7	Manual	N/I	English, Chinese +
**Zarit Caregiver Burden Interview (ZBI)** (Lu et al., 2009; Özer et al., 2012) Turkish, Chinese versions	Generic	Caregiver Burden	Total Score	29 (22 Revised) / 5-Point Scale	Present	Self-Report<br>Paper and Pencil; Online	~6	Manual; Online	Yes	English + Multiple Languages

Instrument	Type	Construct	Domains or Scales or Indices	Number of Items / Response Scale	Timeframe of Response	Administration Method**	Time to Complete, Minutes	Scoring	Licensing or Cost	Minimum Available Languages
**The Care of My Child – CHD modification of Caregiving Burden Scale** (Svavarsdottir & McCubbin, 1996)	Adaptable to Specific Context	Caregiving Demands	N/I	17 / 5-Point Scale	N/I	Self-Report<br>Paper and Pencil	~5	Manual	N/I	English
**Quality of Life and Health-Related Quality of Life**										
**36-Item Short-Form Health Survey (SF-36)** (Ware et al., 2000) *English, Turkish, Persian, Dutch versions; Amharic translation	Generic	Functional Health and Wellbeing	Physical Functioning; Role (Physical; Bodily Pain; General Health; Vitality); Social Functioning Role (Emotional; Mental Health; Reported Health; Transition; Physical Component Summary Score; Mental Component Summary Score	36 / 5-Point Scale	Past 4 Weeks	Self-Report<br>Paper and Pencil; Online; Personal Digital Assistant	5–10	Manual; Software	Yes	English + >100 Languages
**12-Item Short-Form Health Survey (SF-12)** (Kodraliu et al., 2001) *Italian version	Generic	Functional Health and Wellbeing	Nil	12 / 5-Point Scale	Past 4 Weeks	Self-Report<br>Paper and Pencil; Computer; Tablet; Smartphone	2–3	Manual; Software	Yes	English + >100 Languages
**Ulm Quality of Life Inventory for Parents (ULQIE)** (Goldbeck & Storck., 2002; Goldbeck & Melches., 2006)^	Generic	Quality of Life	Physical and Daily Functioning; Satisfaction with Support from the Family; Emotional Stability; Self-Development; Wellbeing; Total Score	29 / 5-Point Scale	Past 7 Days	Self-Report<br>Paper and Pencil	~8	Manual	N/I	German

Instrument	Type	Construct	Domains or Scales or Indices	Number of Items / Response Scale	Timeframe of Response	Administration Method**	Time to Complete, Minutes	Scoring	Licensing or Cost	Minimum Available Languages
**Perceived Quality of Life Scale (PQOL)** (Patrick et al., 2000) Extended version	Generic	Quality of Life	Total Score	20 / 10-Point Scale	Present	Self-Report<br>Paper and Pencil	~5	Manual	Y	English + 4+ Languages
**World Health Organization Quality of Life (WHOQOL-100)** (Kuyken et al., 1995; Power et al., 1998) #Modified	Generic	Quality of Life	Physical Health; Psychological Functions; Level of Independence; Social Relationships; Environment; Spirituality/Religion/Personal beliefs	100 / 5-Point Scale	Two Weeks (Can Adapt)	Self-Report<br>Paper and Pencil	30	Manual	No	English + >30 Languages
**World Health Organization Quality of Life Scale BREF (WHOQOL-BREF)** (Skevington et al., 2004; Whoqol., 1998) *English, Turkish, Sri Lankan versions	Generic	Quality of Life	Physical Health; Psychological; Social Relationships; Environment	26 (Turkish 27) / 5-Point Scale	Two Weeks (Can Adapt)	Self-Report<br>Paper and Pencil	~7	Manual	No	English + 10+ Languages
**Measure of Parental, Sibling and Child Quality of Life** (Azhar et al., 2016)	Study-Specific	Quality of Life	5-part questionnaire Part 3 - Parent Quality of Life; Part 4 - Sibling Quality of Life	N/I / Yes/No Response	N/I	Self-Report and Parent-Proxy Report<br>Paper and Pencil	N/I	Manual	N/I	Arabic

Instrument	Type	Construct	Domains or Scales or Indices	Number of Items / Response Scale	Timeframe of Response	Administration Method**	Time to Complete, Minutes	Scoring	Licensing or Cost	Minimum Available Languages
**Linear Analogue Scale Quality of Life** (Williams et al., 1990; Moons et al., 2006)	Illness-Focused	Quality of Life	N/A	1 / 0-100 Scale	Current	Self-Report<br>Paper and Pencil; Electronic	1	Manual	Yes	>200 Languages
**Pediatric Quality of Life (PedsQL) Family Impact Module** (Varni et al., 2004)	Illness-Focused	Parent Health-Related Quality of Life; Family Functioning	Parent Health-Related Quality of Life: Physical Functioning; Emotional Functioning; Social Functioning; Cognitive Functioning; Communication; Worry Family Functioning: Daily Activities; Family Relationships	36 / 5-Point Scale	4 Weeks	Self-Report	~9	Manual	Yes	English + >40 Languages
**Family Member Well-being Index (FMWB)** (McCubbin & Patterson, 1983)	Generic	Family Member Wellbeing/Adjustment	Total Score	8 / 10-Point Scale	Past Month	Self-Report<br>Paper and Pencil	~2	Manual	N/I	English
**Life Satisfaction Questionnaire (LiSat-11)** (Fugl-Meyer et al., 2002)	Generic	Life Satisfaction	Total (Mean) Score	11 / 6-Point Scale	Present	Self-Report<br>Paper and Pencil	~3	Manual	N	Swedish, English
**Family Impact or Functioning**										
**Psychosocial Assessment Tool (PAT 2.0)** (Pai et al., 2008) #Modified	Illness-Focused	Psychosocial Risk	Family Structure and Resources; Family Social Support; Family Problem; Parent Stress Reactions; Family Beliefs; Child Problems; Sibling Problems	15 Item Sets	Present	Self-Report<br>Paper and Pencil; Online	10	Manual; Online; Software	Yes	English + >10 Languages

Instrument	Type	Construct	Domains or Scales or Indices	Number of Items / Response Scale	Timeframe of Response	Administration Method**	Time to Complete, Minutes	Scoring	Licensing or Cost	Minimum Available Languages
**Family Assessment Device (FAD)** (Epstein, N.B., et al., 1983) *English, Turkish versions	Generic	Family Functioning	Problem Solving; Communication; Roles; Affective Responsiveness; Affective Involvement; Behavior Control; General Functioning	60 / 4-Point Scale	Not Specified	Self-Report<br>Paper and Pencil	15–20	Manual	Yes	English + 27 Languages
**Family Environment Scale (FES)** (Moos, 1990)	Generic	Family Environment (Real, Expected, and Ideal)	3 dimensions; 10 subscales<br>Family Relationship Index: (Cohesion; Expressiveness; Conflict)<br>Personal Growth: (Independence; Achievement Orientation; Intellectual-Cultural Orientation; Active-Recreational Orientation; Moral Religious Emphasis)<br>System Maintenance and Change: (Organization; Control)	90 / True/False Response	Not Specified	Self-Report<br>Paper and Pencil; Online	15–20	Manual	Yes	English + >20 Languages
**Pediatric Quality of Life (PedsQL) Family Impact Module**<br>As above										
**Impact on Family Scale (IOFS)** (Stein & Riessman, 1980; Stein, 1985)	Illness-Focused	Impact of Child Illness on the Family	Financial; Familial/Social; Personal Strain; Mastery; Total Score (Sibling scale removed in development)	24 / 4-Point Scale	Present	Self-Report<br>Paper and Pencil	~8	Manual	N/I	English, French, German, Spanish, Turkish

Instrument	Type	Construct	Domains or Scales or Indices	Number of Items / Response Scale	Timeframe of Response	Administration Method**	Time to Complete, Minutes	Scoring	Licensing or Cost	Minimum Available Languages
**Impact on Family Scale Revised (IOFS-R)** (Stein & Jessop, 2003) *English, German versions	Illness-Focused	Impact of Child Illness on the Family	Total Score + Optional Scales	15 / 4-Point Scale	Present	Self-Report<br>Paper and Pencil	~4	Manual	N/I	English, German
**Family Inventory of Life Events and Changes (FILE)** (McCubbin, Patterson et al., (1983)	Generic	Family Stress/Pile Up of Events	Intrafamilial Strains; Marital Strains; Pregnancy and Childbearing Strains; Finance and Business Strains; Work-Family Transitions and Strains; Illness and Family "Care" Strains; Losses; Transitions "In and Out"; Family Legal Violations	71 / Yes/No Response	12 Months	Self-Report<br>Paper and pencil	~18	Manual	No	English, Hebrew, Spanish (Puerto Rico, Spain)
**Family Hardiness Index (FHI)** (McCubbin, et al., 1986)	Generic	Family Hardiness (i.e., "internal strengths and durability of the family unit")	Commitment, Challenge, Control Subscales; Total Score	20 / 4-Point scale	Present	Paper and pencil	~5 mins	Manual	No	Afrikaans, Chinese, English, Slovenian, Spanish, Thai, Xhosa
**Family Illness Accommodation Questionnaire** (Janus & Goldberg, 1997)	Study-Specific	Illness Accommodation in Family Life	Nil	9 / 4-Point Scale	Frequency Likert Scale	Self-Report<br>Paper and Pencil	~3	Manual	N/I	English

Instrument	Type	Construct	Domains or Scales or Indices	Number of Items / Response Scale	Timeframe of Response	Administration Method**	Time to Complete, Minutes	Scoring	Licensing or Cost	Minimum Available Languages
**Financial Strain/ Economic Hardship Instrument** (Vinokur & Caplan, 1987)	Generic	Economic Hardship	N/R	3 / 5-Point Scale	Present And Next 2 Months	Self-Report<br>Paper and Pencil	~ 1	Manual	N/I	English
**Coping**										
**Coping Inventory for Stressful Situations (CISS)** (Endler & Parker, 1994) *English and Korean versions	Generic	Coping	Task-Oriented; Emotion-Oriented; Avoidance-Oriented	48 / 5-Point Scale	Not Limited	Self-Report<br>Paper and Pencil	~12	Manual	Yes	English, Korean +
**Coping Health Inventory for Parents (CHIP)** (McCubbin et al., 1981; McCubbin, McCubbin, et al., 1983) #Modified	Illness-Focused	Parental Coping	Pattern 1 – Maintaining Family Integration, Cooperation and an Optimistic Definition of the Situation; Pattern 2 – Maintaining Social Support, Self-Esteem and Psychological Stability; Pattern 3 – Understanding the Medical Situation through Communication with Other Parents and Consultation with the Medical Staff	45 (Full) / 4-Point Scale	Not Limited	Self-Report<br>Paper and Pencil	30	Manual	N/I	English, German, Hindi, Japanese, Kannadan, Korean, Malay, Spanish

Instrument	Type	Construct	Domains or Scales or Indices	Number of Items / Response Scale	Timeframe of Response	Administration Method**	Time to Complete, Minutes	Scoring	Licensing or Cost	Minimum Available Languages
**COPE Inventory** (Carver et al., 1989)	Adaptable to specific context	Coping	Active Coping; Planning; Suppression of Competing Activities; Restraint Coping; Seeking Social Support for Instrumental; Seeking Social Support for Emotional Reasons; Positive Reinterpretation & Growth; Acceptance; Turning to Religion; Focus on Venting Emotions; Denial; Behavioral Disengagement; Mental Disengagement; Alcohol-Drug Disengagement	60 / 4-Point Scale	Not Limited	Self-Report<br>Paper and Pencil	~15	Manual	N/I	Chinese, French, Spanish +
**Brief COPE** (Carver, 1997)	Adaptable to specific context	Coping	Active Coping; Planning; Positive Reframing; Acceptance; Humor; Religion; Using Emotional Support; Using Instrumental Support; Self-Distraction; Denial; Venting; Substance Use; Behavioral Disengagement; Self-Blame	28 Items / 4-Point Scale	Not Limited	Self-Report<br>Paper and Pencil	~7	Manual	No	English, Chinese, French, German, Greek, Korean, Spanish
**Utrecht Coping List (UCL)** (Schreurs et al., 1993)	Generic	Coping	Active Tackling; Seeking Social Support; Palliative Reacting; Avoiding; Passive Reacting; Reassuring Thoughts; Expression of Emotions	44 / 4-Point Scale	N/I	Self-Report<br>Paper and Pencil	~11	Manual	N/I	Dutch, English

Instrument	Type	Construct	Domains or Scales or Indices	Number of Items / Response Scale	Timeframe of Response	Administration Method**	Time to Complete, Minutes	Scoring	Licensing or Cost	Minimum Available Languages
**Ways of Coping Questionnaire (WCQ)** (Folkman & Lazarus, 1985) *English and Modified Hebrew versions	Adaptable to Specific Context	Coping	Confrontive Coping; Distancing; Self-Controlling; Seeking Social Support; Accepting Responsibility; Escape-Avoidance; Planful Problem Solving; Positive Reappraisal	66 And 44 (Hebrew version) / 4-Point Scale	Past Week	Self-Report<br>Paper and Pencil; Online	10	Manual; Online	Yes	English + >10 Languages
**Coping Scale for Adults, Second Edition-Short Form** (Frydenberg & Lewis, 2014)	Illness-Focused	Coping	Total Score	18 / 5-Point Scale	Present	Self-Report<br>Online	10–15	Online	Yes	English
**Coping Self-Efficacy Scale** (Jackson et al., 2020; Murphy et al., 2017)	Study-Specific	Parental Coping Self-Efficacy	Total (Mean) score	10 / 5-Point Scale	Present	Self-Report<br>Paper and Pencil	~3	Manual	N/I	English
**Coping Self-Efficacy Scale (CSES)** (Chesney et al., 2003) *Revised and translated to Korean for study	Generic	Coping Self-Efficacy	Problem-Focused Coping; Stop Unpleasant Emotions and Thoughts; Get Support from Friends and Family	26 / 11-Point Scale	Present	Self-Report<br>Paper and Pencil	~7	Manual	N/I	English
**Response to Stress Questionnaire (RSQ-CHD)** (Jackson et al., 2017)	CHD-Specific	Stress; Coping	Total Score	14; 57 / 4-Point Scale	Past 6 Months	Self-Report<br>Paper and Pencil	~18	Manual	N/I	English

Instrument	Type	Construct	Domains or Scales or Indices	Number of Items / Response Scale	Timeframe of Response	Administration Method**	Time to Complete, Minutes	Scoring	Licensing or Cost	Minimum Available Languages
**Cognitive Appraisal Scale** Hebrew version (Folkman & Lazarus, 1985; Berant et al., 2003; Berant et al., 2001)	Generic	Cognitive Appraisal of Stress and Coping (Tailored to Motherhood)	N/I	17/21<br>5 pt scale	N/I	Self-Report<br>Paper and Pencil	~5	Manual	N/I	English, Hebrew
**Spiritual Insight and Behavioral Scale** (Sira et al., 2014) Modified	Generic	Use of Spirituality in Coping	N/I	14: Full Scale; 8: Modified / 4-Point Scale	Not/I	Self-Report<br>Online	~4	Manual	N/A	English
**Stress Appraisal**										
**Hassles and Uplifts Scale - Combined (HSUP)** (Lazarus & Folkman, 1989)	Generic	Stress Appraisal	Hassles; Uplifts	53 (X 2) / 4-Point Scale	Specify Past Month/Week/Yesterday/Today/Other	Self-Report<br>Paper and Pencil; Online	5–10	Manual; Online	Yes	English, Creole, French, Spanish
**Life Experiences Survey (LES)** (Sarason et al., 1978)	Generic	Appraisal of Life Stressors	Life Change Scores - Positive; Negative; Total; Balance	57; Section 1: All Respondents (47 Items); Section 2: Students Only (10 Items) / 7-Point Scale	0–6 Months Or<br>7 Months–1 Year	Self-Report<br>Paper and Pencil	~12	Manual	N/I	English, Spanish
**Social Readjustment Rating Scale (SRRS)** (Holmes & Rahe, 1967)	Generic	Appraisal of Life Stressors	N/A	43	Past Year/ Near Future	Self-Report<br>Paper and Pencil	~11	Manual	No	English, French, Spanish

Instrument	Type	Construct	Domains or Scales or Indices	Number of Items / Response Scale	Timeframe of Response	Administration Method**	Time to Complete, Minutes	Scoring	Licensing or Cost	Minimum Available Languages
**Parent Stressor Scale: Infant Hospitalization (PSS-IH)** (Miles & Brunssen, 2003) #Full and Modified versions	Illness-Focused	Parental Perception of Stressors Associated with Hospitalization	Parental Role Alteration; Infant Appearance/ Behavior; Sights and Sounds; Total Score	28	Present	Self-Report<br>Paper and Pencil	~7	Manual	N/I	English +
**Parental Stressor Scale: Neonatal Intensive Care Unit (PSS:NICU)** (Miles et al., 1993)	Illness-Focused	Parental Perception of Stressors Associated with Hospitalization	Infant Behavior and Appearance; Parental Role Alteration; Sights and Sounds	46 / 5-Point Scale	Not Specified	Self-Report<br>Paper and Pencil	~12	Manual	N/I	English +
**Neonatal Unit Parental Stressor Scale (NUPS)** (Reid et al., 2007)	Illness-Focused	Stress Appraisal/ Psychological Distress in First Two Weeks of NICU Stay	Social/Practical Stress; Illness and Treatments Stress; Role and Relationship with Infant Stress; Total Score	47 / 6-Point Scale	N/R	Self-Report<br>Paper and Pencil	~12	Manual	N/I	English +
**Feeling of Tension Questionnaire** (Campbell et al., 1986; Campbell et al., 1992)	Illness-Focused	Tension at Stress Points	N/R	14 / 5-Point Scale	Present	Self-Report<br>Paper and Pencil	~1	Manual	N/I	English
**Stress Awareness, Study-Specific Instrument** (Rahimianfar et al., 2015)	Study-Specific	Stress Awareness	N/I	N/I	N/I	Self-Report<br>Paper and Pencil	N/I	Manual	N/I	N/I

Instrument	Type	Construct	Domains or Scales or Indices	Number of Items / Response Scale	Timeframe of Response	Administration Method**	Time to Complete, Minutes	Scoring	Licensing or Cost	Minimum Available Languages
**Response to Stress Questionnaire (RSQ-CHD)** As above										
**Cognitive Appraisal Scale** Hebrew version As above										
**Couple Relationship Satisfaction or Strain**										
**Dyadic Adjustment Scale (DAS)** (Spanier, 1976)	Generic	Quality of Adjustment in Marriage/Dyads	Dyadic Satisfaction; Dyadic Cohesion; Dyadic Consensus; Affectional Expression	32 / 6-Point Scale	Not Specified	Self-Report<br>Paper and Pencil	5–10	Manual	Yes	English +
**Evaluating and Nurturing Relationship Issues Communication and Happiness Scale (ENRICH)** (Fowers & Olson, 1989) *#Shortened Hebrew version; expanded Finnish translation	Generic	Marriage/ Relationship Problems and Strengths	Idealistic Distortion; Marital Satisfaction; Personality Issues; Communication; Conflict Resolution; Financial Management; Leisure Activities; Sexual Relationship; Children and Parenting; Family and Friends; Equalitarian Roles; Religious Orientation; Marital Cohesion; Marital Change	125 / N/R	N/I	Self-Report<br>Online	~32	Online	N/I	English + >10 Languages

Subscales of:

1. Family Inventory of Life Events and Changes (FILE)
2. Parenting Stress Index (PSI)
3. Parenting Stress Index-Short Form (PSI-4-SF)
4. Swedish Parenthood Stress Questionnaire (SPSQ)

Instrument	Type	Construct	Domains or Scales or Indices	Number of Items / Response Scale	Timeframe of Response	Administration Method**	Time to Complete, Minutes	Scoring	Licensing or Cost	Minimum Available Languages
**Sibling Psychosocial Outcomes**										
**Sibling Perception Questionnaire (SPQ)** (Lobato & Kao, 2002; Sahler & Carpenter, 1989) Adapted	Illness-Focused	Sibling Adjustment	Interpersonal Relationships; Responses; Communication; Fear of Disease	23 / 4-Point Scale; 7-Point Scale (Finnish version)	Not Specified	Self-Report<br>Paper and Pencil	~6	Manual	N/I	English, German
**Perception of Effect on Sibling Scale** (Breslau et al., 1981)	Illness-Focused	Effect of Caregiving on Well Sibling	Total Score	4 / 5-Point Scale	Present	Parent-Report<br>Paper and Pencil	~1	Manual	N/I	English
**Sibling Relationship Questionnaire (SRQ)** (Furman & Buhrmester, 1985) *Korean Version	Generic	Perceived Sibling Relationship	Warmth/Closeness Relative Status/Power; Conflict; Rivalry	39 / 5-Point Scale	Present	Self-Report<br>Paper and Pencil	~10	Manual	No	English
**Pediatric Quality of Life (PedsQL) Generic Core Scales** (Varni et al., 1999) *Urdu translation for study	Generic	Quality of Life	Physical Health; Psychosocial Health Emotional Health Social Functioning; School Functioning; Total Score; Physical Health Summary Score; Psychosocial Health Summary Score	23 / 5-Point Scale	1 Month	Self-Report and Parent-Proxy Report<br>Paper and Pencil, Electronic	~6	Manual; Electronic	Yes	English, Spanish +

Instrument	Type	Construct	Domains or Scales or Indices	Number of Items / Response Scale	Timeframe of Response	Administration Method**	Time to Complete, Minutes	Scoring	Licensing or Cost	Minimum Available Languages
**Pediatric Quality of Life (PedsQL) Cognitive Functioning Scale** (Varni et al., 2002) *Urdu translation for study	Generic	Cognitive Functioning	Total Score	6 / 5-Point Scale	1 Month	Self-Report and Parent-Proxy Report<br>Paper and Pencil	~2	Manual	Yes	English +

Subscales of:

1. Measure of Parental, Sibling, and Child Quality of Life
2. Impact on Family Scale (IOFS)/Impact on Family Scale-Revised (IOFS-R)
3. Psychosocial Assessment Tool (PAT 2.0)

Note: N/A – Not Applicable; N/I – Not Identified/Available; **Presumed paper and pencil based unless otherwise specified; *English version data presented; #Full version data presented; ~Measure length not reported, length estimate based on completion of 4 items/minute; ^Secondary Source.

Table S6: Instrument Psychometric Properties.

Instrument	Domains or Scales or Indices	Original Instrument Development Properties					CHD Study Properties	
		Sample	Reliability		Validity		Reference (Country)	Reliability
			Internal Consistency	Test-Retest	Construct	Criterion		
**Psychological Functioning and Distress**								
**Symptom Checklist-90-Revised (SCL-90-R)** (Derogatis & Cleary, 1977; Derogatis et al., 1976)	Symptom Scales: Somatization; Obsessive-Compulsive; Interpersonal Sensitivity; Depression; Anxiety; Hostility; Phobic Anxiety; Paranoid Ideation; Psychoticism<br><br>Global Indices: General Severity Index; Positive Symptom Distress Index; Positive Symptom Total	Psychiatric Outpatients (USA)	α=.86 Somatization; .86 Obsessive-Compulsive; .86 Interpersonal Sensitivity; .90 Depression; .85 Anxiety; .84 Hostility; .82 Phobic Anxiety; .80 Paranoid Ideation; .77 Psychoticism	N/R	Convergent: r=.66/.62 with somatization and body/organic symptoms; .68–.75 depression and depression cluster; .57 anxiety and anxiety cluster scales of Minnesota Multiphasic Personality Inventory	N/R	Lawoko & Soares, 2006 (Sweden) Depression, Anxiety, Somatization scales only	α=.84–.90 (Time 1 and Time 2)
**SCL-90-R Turkish version** (Yildiz et al., 2009)^		N/I	α=0.9	r=.65–.87	N/I	N/I	Yildiz et al., 2009 (Turkey) Depression, Anxiety, Somatization scales only	α=.90 Depression; .81 Anxiety; .84 Somatization
**SCL-90-R Dutch version** (Arrindell & Ettema, 2003)		N/I	N/I	N/I	N/I	N/I	van der Mheen et al., 2019 (The Netherlands)	N/R
**Brief Symptom Inventory (BSI)** (Derogatis & Melisaratos, 1983)	Symptom Scales: Somatization; Obsessive-Compulsive; Interpersonal Sensitivity; Depression; Anxiety; Hostility; Phobic Anxiety;	Psychiatric Out- and In-Patients; non-Patients (USA)	α=.80 Somatization; .83 Obsessive-Compulsive; .74 Interpersonal Sensitivity; .85	r=.68 Somatization; .85 Obsessive-Compulsive; .85	Convergent: r=.38/.36 somatization and body/organic symptoms); .67–.72 depression and depression cluster; .57	N/R	Brosig, et al., 2007b (USA)<br><br>Davis et al., 1998 (USA)	N/R<br><br>α=.96 (sum score)

Instrument	Domains or Scales or Indices	Original Instrument Development Properties					CHD Study Properties	
		Sample	Reliability		Validity		Reference (Country)	Reliability
			Internal Consistency	Test-Retest	Construct	Criterion		
	Paranoid Ideation; Psychoticism<br><br>Global Indices: General Severity Index; Positive Symptom; Distress Index; Positive Symptom Total		Depression; .81 Anxiety; .78 Hostility; .77 Phobic Anxiety; .77 Paranoid Ideation; .71 Psychoticism	Interpersonal Sensitivity; .84 Depression; .79 Anxiety; .81 Hostility; .91 Phobic Anxiety; .79 Paranoid Ideation; .78 Psychoticism (2 weeks; 60 non-Patients)	anxiety and anxiety cluster scales of Minnesota Multiphasic Personality Inventory		Doherty et al., 2009 (Global Severity Index only) (UK)	N/R
							McCusker et al., 2012 (UK)	N/R
							Pinto et al., 2016 (USA)	N/R
**Brief Symptom Inventory 18 (BSI-18)** (Derogatis, 2001)	Symptom Scales: Somatization; Depression; Anxiety<br><br>Global Severity Index	N/I	N/I	N/I	N/I	N/I	Bishop et al., 2019 (USA)	α=.90
**BSI-18 Chinese version** (Wang et al., 2013)^		Drug Users (China)	Kuder Richardson Coefficient (KR-20) .76 Somatization; .77 Depression; .83 Anxiety; .91 General Psychological Distress	N/R	Confirmatory Factor Analysis: original 3 factor model supported	N/R	Li et al., 2018 (China)	α=.94
**General Health Questionnaire (GHQ)** (Goldberg & Blackwell, 1970)	Total Score	General Practice Attendees (UK)	r=.92 Inter-Rater reliability between psychiatrists	N/R	N/R	95.8% Sensitivity; 87.8% Specificity with Clinical Interview Schedule	Menahem et al., 2008 (Australia)	N/R

Instrument	Domains or Scales or Indices	Original Instrument Development Properties					CHD Study Properties	
		Sample	Reliability		Validity		Reference (Country)	Reliability
			Internal Consistency	Test-Retest	Construct	Criterion		
**GHQ-30 Italian version** (Fontanesi et al., 1985)	Total Score	General Practice Attendees (Italy)	N/R	N/R	N/R	75.4% Sensitivity; 50.8% Specificity with Clinical Interview Schedule	Bevilacqua et al., 2013 (Italy)	N/R
**GHQ-28 Dutch version** (Spijkerboer et al., 2007)^	Somatic Symptoms; Anxiety; Insomnia; Social Dysfunction; Severe Depression	N/I	α=.94	N/I	N/I	N/I	Spijkerboer et al., 2007 (The Netherlands)	N/R
							Utens et al., 2000 (The Netherlands)	N/R
							Dulfer et al., 2015 (The Netherlands)	N/R
**GHQ-28 Chinese version** (Chen et al., 2010)		N/I	N/I	N/I	N/I	N/I	Guan et al., 2013 (China)	N/R
**GHQ-12 Dutch version** (Koeter & Ormel, 1991)	Total Score	N/I	NI	N/I	N/I	N/I	Jantien Vrijmoet-Wiersma et al., 2009 (The Netherlands)	α=.92 Total Score
**GHQ-12 Spanish version** (Humphreys et al., 1991)		N/I	N/I	N/I	N/I	N/I	Lopez et al., 2016 (Chile)	N/R
**Mental Health Inventory (MHI)** Hebrew version	Psychological Wellbeing; Psychological Distress	N/I	α=.96 Total Score	N/I	N/I	N/I	Berant et al., 2001 (Israel)	α=.93 Total Score (Time 1); .95 (Time 2) (healthy and CHD)

Instrument	Domains or Scales or Indices	Original Instrument Development Properties					CHD Study Properties	
		Sample	Reliability		Validity		Reference (Country)	Reliability
			Internal Consistency	Test-Retest	Construct	Criterion		
(Shenaar-Golan, 2015)^								
**Structured Clinical Interview for DSM-IV Clinical version (SCID 1)** (El Missiry et al., 2003) Arabic version	Mood; Psychotic Disorder; Substance Abuse; Anxiety; Somatoform Disorders; Eating Disorders; Adjustment Disorders	N/I	N/I	N/I	N/I	N/I	Awaad & Darahim, 2015 (Egypt)	N/R
**Perceived Stress Scale (PSS-10)** (Cohen & Williamson, 1988)	Total Score	Residents (USA)	α=.78	N/R	r=.39/.26 (past/ present experiences of stress; .32,-.09, -.27 (Life Events Scale subscales); -31 (hours worked); .34/.05 (Job Responsibilities Scales); .06 (Workload demand); .15-.22 (self-reported health); .32/.28/.34 (psychosomatic index factors); .22 (Health Services Utilization Scale); -.09-.26 (Health behaviors measures)	N/R	Miller et al., 2021 (USA)	N/R
**General Stress Item** (Elo et al., 2003)	Total Score	Mixed Working Populations (Finland and mixed Nordic)	N/R	N/R	r=-.31 (Perceived Health); -.14 (Diagnosed Health); -.19 (Work Ability Index); -.07 (Job Control); 0.30 (Quantitative	Concurrent: r=.51 (Maslach's Burnout Inventory Emotional Exhaustion);	Jackson et al., 2020 (Australia)	N/R

Instrument	Domains or Scales or Indices	Original Instrument Development Properties					CHD Study Properties	
		Sample	Reliability		Validity		Reference (Country)	Reliability
			Internal Consistency	Test-Retest	Construct	Criterion		
					Overload); -.15 (Supervisory Support); -.17 Social Climate	-.55/.53 (General Health Questionnaire Mental Health /Sleep); -.63/-.58 (SF-36 Mental Health/ Vitality); -.24 (LOT Optimism)		
**Index of Clinical Stress** (Abell, 1991)	Total Score	Patients and Family Members from a Family Practice Residency Program (USA)	α=.96 Overall Reliability	N/R	Convergent: r=.45 (Index of Family Relations); .75 (Generalized Contentment Scale); .24 (Family Inventory of Life Events and Changes)<br><br>Discriminant: r=-.07 (age); .08 (gender); -.08 (race)	N/R	Kumar et al., 2019 (India)	N/R
**Depression Anxiety and Stress Scale (DASS)** (Lovibond & Lovibond, 1995)	Depression; Anxiety; Stress	Behavioral Program for Depression Volunteers, University Students, & Psychiatric Outpatients	α=.91 Depression; .84 Anxiety; .90 Stress	N/R	Convergent: r=.81 for Anxiety scale with Beck Anxiety Inventory; .74 for Depression with Beck Depression Inventory<br><br>Discriminant: r=.58 for Anxiety scale with Beck Depression	N/I	Tallon et al, 2015 (Australia)<br><br>Roberts et al., 2021 (Canada) - Depression and Anxiety scales only	N/R<br><br>N/R

Instrument	Domains or Scales or Indices	Original Instrument Development Properties					CHD Study Properties	
		Sample	Reliability		Validity		Reference (Country)	Reliability
			Internal Consistency	Test-Retest	Construct	Criterion		
					Inventory; .54 for Depression scale with Beck Anxiety Inventory<br><br>Confirmatory Factor Analysis: 3 factor model significantly better than two (Depression and Anxiety/Stress combined)			
**Depression Anxiety Stress Scale-21 Items (DASS-21)** (Antony et al., 1998; Lovibond & Lovibond, 1995)^	Depression; Anxiety; Stress	Normative Sample (Australia)	α=.81 Depression; .73 Anxiety; .81 Stress	N/I	Convergent: r= .69, .70, .68 Stress; .79, .51, .70 Depression; .62 Anxiety scales, .85, .55 Scale with Beck Depression Inventory, Beck Anxiety Inventory, and State-Trait Anxiety Inventory in clinical patients	Concurrent: Scores on Depression and Stress scales highest for those with Major Depressive Disorder; highest Anxiety scores for those with panic disorder<br><br>Discriminant: Discriminate between clinical and non-clinical groups	Blue et al., 2015 (Australia)<br>Denniss et al., 2019 (Australia)<br>Callahan et al., 2019 (USA)	α=.87 Depression; .87 Anxiety; .85 Stress (at baseline)<br>α=.85 Depression; .78 Anxiety; .86 Stress<br>N/R

Instrument	Domains or Scales or Indices	Original Instrument Development Properties					CHD Study Properties	
		Sample	Reliability		Validity		Reference (Country)	Reliability
			Internal Consistency	Test-Retest	Construct	Criterion		
**Hospital Anxiety and Depression Scale (HADS)** (Zigmond & Snaith, 1983)	Depression; Anxiety	General Medical Outpatient Attendees (UK)	Spearman r=.76–.41 Anxiety; .60–.30 Depression	N/R	N/R	Concurrent: r=.54 Anxiety; .79 Depression between self-rated and interviewer assessments of same mood disorder patients	Diffin et al., 2016 (Australia)<br>Rona et al., 1998 (UK)	N/R<br>N/R
**HADS Turkish version** (Aydemir O, 1997)^	Depression; Anxiety	Internal Medicine Clinic Attendees	α=.85 Anxiety; .78 Depression	N/I	Convergent: r=.75 State-Trait Anxiety Inventory; .72 Beck Depression Inventory	N/R	Alkan et al., 2017 (Turkey)	N/R
**HADS Swedish version** (Not identified)	Depression; Anxiety	N/I	N/I	N/I	N/I	N/I	Bratt et al., 2019 (Sweden)	N/R
**Beck Depression Inventory-Second Edition (BDI-2)** (Beck, 1996)	Total Score	Psychiatric Outpatients (USA)	α=.92 Total Score	r=.93 (1 week)	Convergent: r=.93 BDI-IA and BDI-2; .68 and .37 Beck Hopelessness Scale and Scale for Suicidal Ideation; .71 Hamilton Psychiatric Rating Scale for Depression<br>Discriminant: r=.47 Hamilton Rating Scale for Anxiety	N/R	Rychik et al., 2013 (USA)<br>Hancock et al., 2018 (USA)	N/R<br>N/R
**BDI-2 Italian version** (Sica, 2007)	Total Score	Under-graduate Students	α=.80 Total Score	r=.76 (1 month)	Convergent: r=.77 with Depression questionnaire<br>Discriminant: r=.66 with STAI	N/R	Bevilacqua et al., 2013 (Italy)	N/R

Instrument	Domains or Scales or Indices	Original Instrument Development Properties					CHD Study Properties	
		Sample	Reliability		Validity		Reference (Country)	Reliability
			Internal Consistency	Test-Retest	Construct	Criterion		
**BDI-2 Arabic version** (Ghareeb A, 2000)	Total Score	N/I	N/I	N/I	N/I	N/I	Awaad & Darahim, 2015 (Egypt)	N/R
**BDI-2 Turkish version** (Hisli, 1989)	Total Score	N/I	N/I	N/I	N/I	N/I	Uzger et al. 2015 (Turkey)	N/R
**Center for Epidemiologic Studies Depression Scale (CES-D)** (Radloff, 1977)	Total Score	Community and Psychiatric Patients (USA)	α.84–.90	r=.45–.70 across time intervals;.54 where no negative life events reported at either time (2, 4, 6, 8 weeks)	Convergent: r=.44–.54 ; .69–.75 with Hamilton Clinician's Rating scale and Raskin Rating scale at admission and post treatment<br><br>Discriminant: r=-.21–-.55 with Bradburn Positive Affect scale	Concurrent: r= .46–.53 with interviewer depression ratings; distinguishes between general and psychiatric inpatients	McKechnie et al., 2016 (USA)<br>Lisanti et al., 2021a (USA)<br>Lisanti et al., 2021b (USA)	N/R<br>α=.86<br>N/R
**Patient Health Questionnaire (PHQ-9)** (Kroenke & Spitzer, 2002; Kroenke et al., 2001)	Total Score	Primary care & Obstetrics -Gynecology (USA)	α=.89/.86	r=.84 (48 hours)	r=.33–.73 across SF-20 subscales; .39 Disability Days; .24 Physician Visits; .55 Symptom-Related Difficulty	Sensitivity 95–68%; Specificity 84–95%; Likelihood Ratio 6.0–13.6 across scores	Gaskin et al, 2021 (USA)	N/R
**Edinburgh Postnatal Depression Scale (EPDS)** (Cox et al., 1987)	Total Score	New Mothers (UK)	α=.87	N/R	N/R	Sensitivity 85% ; Specificity 87%; Positive Predictive Value 83%	Re et al., 2018 (Australia)<br>Jordan et al., 2014 (Australia)	N/R<br>N/R

Instrument	Domains or Scales or Indices	Original Instrument Development Properties					CHD Study Properties	
		Sample	Reliability		Validity		Reference (Country)	Reliability
			Internal Consistency	Test-Retest	Construct	Criterion		
**State-Trait Anxiety Inventory (STAI)** (Spielberger et al., 1983)	State Anxiety; Trait Anxiety	Working Adults (USA)	α=.93 State Anxiety; .91 Trait Anxiety	r=.62/.34–.51/.36 State Anxiety; .71/.75–.68/.65 Trait Anxiety (High-School students 30 days–60 days)	Convergent/Divergent: r=-.64–79 vs, -.46–48 with Minnesota Multiphasic Personality Inventory in more acutely disturbed neuropsychiatric sample vs less; .70 with Cornell Medical Index; -.07–.07 with academic aptitude and achievement	Concurrent: Form X -T scale r=.75–.77 with IPAT Anxiety scale and .79–.83 with IPAT Manifest Anxiety Scale	Fischer et al., 2012 (USA)	α=.93 State; .92 Trait
							Hoehn et al., 2004 (USA)	N/R
							Lisanti et al., 2017 (USA)	α=.95 State; .91 Trait
							McCusker et al., 2010 (UK)	N/R
							McKechnie et al., 2016 (USA)	N/R
							Menahem et al., 2008 (Australia)	N/R
							Re et al., 2018 (Australia)	N/R
							Rychik et al., 2013 (USA)	N/R
							Rahimianfar at el., 2015 (Iran)	N/R
							Campbell et al., 1995 (USA)	N/R
							Campbell et al., 1992 (USA)	N/R
							Hancock et al., 2018 (USA)	N/R
							Kumar et al., 2019 (India)	N/R

Instrument	Domains or Scales or Indices	Original Instrument Development Properties					CHD Study Properties	
		Sample	Reliability		Validity		Reference (Country)	Reliability
			Internal Consistency	Test-Retest	Construct	Criterion		
							Lisanti et al., 2021a (USA)	α=.97 State; .91 Trait
							Lisanti at al., 2021b (USA)	N/R
							Mussatto et al., 2021 (USA)	N/R
**STAI Dutch version** (de Vries & van Heck, 2013)^		N/I	α=.84–.95 Trait Anxiety	N/I	N/I	N/I	Jantien Vrijmoet-Wiersma et al., 2009 (The Netherlands)	α=.80 State; .77 Trait
**STAI Chinese version** (Chien et al., 2021)^		N/I	α=.86 Trait; .90 State	N/I	N/I	N/I	Chien et al., 2021 (Taiwan)	α=.84 State; .88 Trait
**STAI Turkish version** (Kiliçarskan-Törüner et al., 2012)^		N/I	α=.83–.87 Trait; .94–.96 State Anxiety	r=.71–.86 Trait; .26–.68 State Anxiety (Time frame N/I)	N/I	N/I	Kiliçarskan-Törüner et al., 2012 (Turkey)	N/R
**STAI Korean version** (Kim & Shin, 1978)		N/I	N/I	N/I	N/I	N/I	Uhm & Kim, 2019 (Korea)	α=.95
**Beck Anxiety Inventory Turkish version**	Total Score	Psychiatric Outpatients	α=.93	N/R	Discriminant: r=.69 (anxious/depressed/ mixed diagnostic groups)	Concurrent: r=.45/.53 (STAI - Trait/State); .46 (Beck	Uzger et al., 2015 (Turkey)	N/R

Instrument	Domains or Scales or Indices	Original Instrument Development Properties					CHD Study Properties	
		Sample	Reliability		Validity		Reference (Country)	Reliability
			Internal Consistency	Test-Retest	Construct	Criterion		
(Ulusoy et al., 1998)						Depression Inventory); .34 (Beck Hopelessness Scale); .41 (Automatic Thoughts Questionnaire)	Coşkuntürk et al., 2018 (Turkey)	N/R
**Generalized Anxiety Disorder-7 (GAD-7)** (Spitzer et al., 2006)	Total Score	Primary Care Sites (USA)	α=.92	r=.83 (1 week)	Convergent: r=.72 (Beck Anxiety Inventory); .74 (Anxiety subscale Symptom Checklist-90); .75 (Patient Health Questionnaire Depression measure)	Elevated mean scores consistent with patients with Generalized Anxiety Disorder diagnosed with Mental Health Professional Interview (SCID-IV GAD section)	Gaskin et al., 2021 (USA)	N/R
**Self-Rating Anxiety Scale (SAS)** (Zung, 1971)	Index Score	Psychiatric Inpatients, Outpatient, and non-Patients (USA)	N/R	N/R	Convergent: r=.66/.74 (Anxiety Status Inventory all patients /anxiety disorder diagnosis); .30 (Taylor Manifest Anxiety Scale)	N/R	Zhang et al., 2021 (China)	N/R
**Visual Analogue Scale (Anxiety)** (Hornblow & Kidson, 1976)	Total Score	Medical Students and Psychiatric Patients	N/R	r=.32 (6 months)	N/R	Concurrent: r=.84 (students) and .65 (patients) with State-Trait Anxiety Scale - State version	Werner et al., 2019 (France)<br>Lisanti et al., 2021a (USA)	N/R<br>N/R

Instrument	Domains or Scales or Indices	Original Instrument Development Properties					CHD Study Properties	
		Sample	Reliability		Validity		Reference (Country)	Reliability
			Internal Consistency	Test-Retest	Construct	Criterion		
**Taylor Manifest Anxiety Scale (TMAS)** (Taylor, 1951, 1953)	Total Score	Introductory Psychology Students (USA)	N/R	r=.89 (3 weeks); .82 (5 months); .81 (9–17 months)	N/R	N/R	Awaad & Darahim, 2015 (Egypt)	N/R
**Impact of Event Scale-Revised (IES-R)** (Weiss et al., 2007; Weiss & Charles, 1997)^	Intrusion; Avoidance; Hyperarousal; Total Score	Emergency Personnel; Earthquake Survivors (USA)	α=.87–.91 Intrusion; .84–.86 Avoidance; .79–.90Hyperarousal	r=.57; .94 Intrusion; .51; .89 Avoidance; .59; .92 Hyperarousal (6 Months)	N/R	Concurrent: r=.47–.86 with Post Traumatic Stress Disorder Impact of Events (2); .47–.66 with Clinician-Administered PTSD Scale in a motor vehicle accident sample; .45–.64 with State-Trait Anxiety Inventory; .58–.70 with Beck Anxiety Inventory; .53–.72 with BDI-2<br><br>Distinguishes between individuals with and without Post Traumatic Stress Disorder with sensitivity	Rychik et al., 2013 (USA) - Prenatal Diagnosis<br><br>McKechnie et al., 2016 (USA) - Diagnosis and Treatment<br><br>Mussatto et al., 2021 (USA)	N/R<br><br>N/R<br><br>N/R

Instrument	Domains or Scales or Indices	Original Instrument Development Properties					CHD Study Properties	
		Sample	Reliability		Validity		Reference (Country)	Reliability
			Internal Consistency	Test-Retest	Construct	Criterion		
						74.5% and specificity 63.1.%		
**PTSD Checklist-Civilian Version (PCL-C)** (Weathers et al., 1993)	Symptom Subcategories: Intrusive Thoughts; Avoidance of Stimuli Related to the Traumatic Event; Hypervigilance; Total Score Only	Vietnam Veterans Trauma Victims (USA)	α=.89–.93 (symptom clusters); . .96/.97 All symptoms	r=.96 (Interval not specified)	Convergent: r=.93 with Mississippi Post Traumatic Stress Disorder Scale; .77 with Minnesota Multiphasic Personality Inventory - 2; .90 with Impact of Event Scale; .46 with Combat Exposure Scale; Significant correlations across all scale items with all Clinician-Administered PTSD (CAPS) scale items except psychogenic amnesia and hypervigilant	Sensitivity 82% and Specificity of 83% at recommended cut-off of 50	Cantwell-Bartl & Tibballs, 2017 (Australia)	N/R

Instrument	Domains or Scales or Indices	Original Instrument Development Properties					CHD Study Properties	
		Sample	Reliability		Validity		Reference (Country)	Reliability
			Internal Consistency	Test-Retest	Construct	Criterion		
**Posttraumatic Diagnostic Scale (PDS)** (Foa et al., 1997)	Reexperiencing; Avoidance; Arousal; Total Symptom Severity	Trauma Victims (USA)	α=.78 Reexperiencing; .84 Avoidance; .84 Arousal; .92 Total Symptom Severity	r=.77 Reexperiencing; .81 Avoidance; .85 Arousal; .83 Total Symptom Severity (2–3 weeks)	Convergent: Kappa=.65, 82% agreement between PDS and PTSD module of the Structured Clinical Interview	Concurrent: r=.67–.79 across all PDS scales with all scales of Beck Depression Inventory, .62–.74 with State-Trait Anxiety Inventory, .51–.80 with IES-R Sensitivity 89%, Specificity 75%	Medoff-Cooper et al., 2020 (USA)	N/R
**PDS German version** (Griesel et al., 2006)	Reexperiencing/ Avoidance; Emotional Numbing/ Hyperarousal; Hypervigilance/ Exaggerated Startle Response; Total Symptom Severity	Trauma Survivors (Germany)	α=.90 Reexperiencing /Avoidance; .89 Numbing/ Hyperarousal; .88 Hypervigilance/ Exaggerated Startle Response; .94 Total Symptom Severity	N/R	Convergent/Discriminant: r = .41–.76 across all scales with Clinician-Administered PTSD scales and total scores; .38–.72 Revised Impact of Events Scale; .43–.75 Beck Depression Inventory; .41–.68 State-Trait Anxiety Inventory; Significant correlation with SCID social phobia diagnosis	100% Sensitivity, 64% Specificity	Landolt et al., 2011 (Switzerland) Modified to surgery related post-traumatic stress post discharge	α=.88/.85 Total Score (Mothers/Fathers)
							Helfricht et al., 2008 (Switzerland)	α=.84–.88

Instrument	Domains or Scales or Indices	Original Instrument Development Properties					CHD Study Properties	
		Sample	Reliability		Validity		Reference (Country)	Reliability
			Internal Consistency	Test-Retest	Construct	Criterion		
**Acute Stress Disorder Scale (ASDS)** (Bryant et al., 2000)	Dissociation; Re-experiencing; Avoidance; Arousal; ASDS Total Score	Trauma Survivors (Australia)	α=.96 Total Score; .84 Dissociation; .87 Re-experiencing, .92 Avoidance; .93 Arousal	r=.94 Total Score; .85 Dissociation; .94 Re-experiencing; .89 Avoidance; .94 Arousal (2–7 days)	Convergent: r=.86 (item and total scores) with Acute Stress Disorder Interview; .81, .87 with Impact of Event Scale Intrusion and Avoidance scales; .78 with Beck Anxiety Scale; .18 with Dissociation Experiences Scale	Sensitivity of 91% and specificity of 93% at optimal cut-off score of 56, although 33% falsely identified to develop PTSD.	Franich-Ray et al., 2013 (Australia)	α=.91/.88 Total Score; .87/.84 Dissociation; .70/.73 Re-experiencing; .81/.75 Avoidance; .83/.74 Arousal (Mother/Father)

Instrument	Domains or Scales or Indices	Original Instrument Development Properties					CHD Study Properties	
		Sample	Reliability		Validity		Reference (Country)	Reliability
			Internal Consistency	Test-Retest	Construct	Criterion		
**Maslach's Burnout Inventory (MBI)** (Maslach et al., 1996)	Emotional Exhaustion; Depersonalization; Personal Accomplishment	Health and Service Occupations (USA)	α=.90 Emotional Exhaustion; .79 Depersonalization; .71 Personal Accomplishment	r=.82/.60 Emotional Exhaustion; .60/.54 Depersonalization; .80/.57 Personal Accomplishment (Social Welfare Grad Students and/or Health Agency Administrators 2–4 weeks/ Teachers 1 yr)	Convergent: r=.28-42/.32-.56. Emotional Exhaustion/ Depersonalization in Mental Health Workers; .31 Emotional Exhaustion and More Direct Contact in Physicians; .20-.34/.24-.25 Emotional Exhaustion/ Personal Accomplishment in Police Officers and Spouses<br>Discriminant: No correlation with Crowne-Marlowe Social Desirability scales	N/R	Cantwell-Bartl & Tibballs, 2017 (Australia)	N/R
**Ottawa Mood Scale** (Cheng, 2011)	Arousal/Self-Regulation; Mood; Anger; Worry; Stress	N/R	N/R	N/R	N/R	N/R	Kumar et al., 2019 (India)	N/R
**Penn State Worry Questionnaire (PSWQ)** (van Rijsoort et al., 1999)	Total Score	College Psychology Students (USA)	α=.91–.95	r=.92 (8–10 weeks); .75/.74 (2/4 weeks)	r=.64 STAI-Trait; .49 STAI-State; .36 Beck Depression Inventory; .58/.40 Emotionality /Worry scales of Test Anxiety Inventory; .33 Self-Handicapping Scale); -0.09 Marlowe -Crowne Social Desirability Scale; .44	Able to significantly discriminate between GAD and PTSD + among criterial GAD levels using DSM-III-R	van der Mheen et al., 2019 (The Netherlands)	N/R

Instrument	Domains or Scales or Indices	Original Instrument Development Properties					CHD Study Properties	
		Sample	Reliability		Validity		Reference (Country)	Reliability
			Internal Consistency	Test-Retest	Construct	Criterion		
					% of Days Feeling Tension; .64 % of Days Feeling Worried; .26–.38 Self-Consciousness Scale subscales; .31–.61 Reactions to Tests; .39 Perfectionism Scale; .47 General Time Urgency; .23 Nervous Energy subscale			
**Maternal Worry Scale** (DeVet & Ireys, 1998)	Total Score	Mothers Of Children with Juvenile Rheumatoid Arthritis, Sickle Cell Anemia, and Diabetes (USA)	α=.94 Total Score	r=.84 (Juvenile Rheumatoid Arthritis subsample 2 weeks)	N/R	Concurrent: r=.34 Beck Depression Inventory; .39 and .27 Psychiatric Symptom Index<br><br>Discriminate Mothers of Children with Greater Disease Severity	Doherty et al., 2009 (UK)<br>McCusker et al., 2012 (UK)<br>McCusker et al., 2010 (UK)	N/R<br>N/R<br>N/R
**Beck Hopelessness Scale (BHS)** (Beck et al., 1974)	Total Score	Hospitalized Patients with Recent Suicide Attempt (USA)	α=.93 Total Score	N/R	N/R	Concurrent: r=.62 Clinical Ratings of Hopelessness in General Practice and Attempted	Lawoko & Soares, 2006 (Sweden)	α=.80-.84

Instrument	Domains or Scales or Indices	Original Instrument Development Properties					CHD Study Properties	
		Sample	Reliability		Validity		Reference (Country)	Reliability
			Internal Consistency	Test-Retest	Construct	Criterion		
						Suicide samples; .63 Stuart Future Test; .63 Beck Depression Inventory Pessimism Item		
**Beck Hopelessness Scale (BHS) Spanish version** (Aguilar Garcia-Iturropse et al., 1995)		N/I	N/I	N/I	N/I	N/I	Lopez et al., 2016 (Chile)	N/R
**Herth Hope Index (HHI) Chinese version** (Chan et al., 2012)	Total Score	Patients with Heart Failure (Hong Kong)	α=.80–.89	ICC=.86 (2 weeks)	Convergent: r=.40 Rosenberg's Self-Esteem Scale - Chinese version<br>Discriminant: r=-.40 Hamilton Repression Rating Scale; Confirmatory Factor Analysis=.96 Herth Hope Index (English)	N/R	Li et al., 2018 (China)	α=.89
**Life Orientation Test (LOT)** (Scheier & Carver, 1985)	Total Score	Under-graduate students (USA)	α.76	r=.79 4 weeks	Convergent: r=.34 (Rotter's Internal-External Control Scale); .48 (Rosenberg's Self-Esteem Scale); .26 (Crowne-Marlowe	N/R	Hoehn et al., 2004 (USA)	N/R

Instrument	Domains or Scales or Indices	Original Instrument Development Properties					CHD Study Properties	
		Sample	Reliability		Validity		Reference (Country)	Reliability
			Internal Consistency	Test-Retest	Construct	Criterion		
					Social Desirability Scale)<br>Discriminant: r=-.47 (Beck Hopelessness Scale); r=-.49 (Beck Depression Inventory); -.55 (Perceived Stress Scale)			
**Emotional Aspects of Having a Child with CHD, Study-Specific Instrument** (Blue et al., 2015)	Total Score	Parents Attending Cardiac Surgical Pre-Admission Clinic (Australia)	N/R	N/R	N/R	N/R	Blue et al., 2015 (Australia)<br>Hunt et al., 2020	N/R<br>N/R
**Personal Feelings Questionnaire (PFQ-2**) (Harder & Zalrna, 1990)	Shame; Guilt	College Students (USA)	α=.78 Shame; .72 Guilt	r=.91 Shame; .85 Guilt (Sample subset 2 weeks)	Convergent: r=.41/.39 Beck Depression Inventory; .39/.46 Self-Derogation; .23/.07 Social anxiety for Shame/Guilt Scales	N/R	Blue et al., 2015 (Australia)	N/R
**Reaction to Diagnosis, Study-Specific Instrument** (Cohn, 1996)	Feelings Items	Parents of Children with CHD	N/R	N/R	N/R	N/R	Cohn et al., 1996 (USA)	N/R
**Caregivers' Behavior Rating Scale (CBRS)** (Campbell et al., 1992)	Behavior/Distress	Parents of Children with CHD Undergoing Cardiac Catheter (USA)	N/R	N/R	N/R	N/R	Campbell et al., 1992 (USA)	r=.85 Interrater Reliability

Instrument	Domains or Scales or Indices	Original Instrument Development Properties					CHD Study Properties	
		Sample	Reliability		Validity		Reference (Country)	Reliability
			Internal Consistency	Test-Retest	Construct	Criterion		
**Parenting Stress and Caregiver Burden**								
**Parenting Stress Index (PSI)** (Abidin, 2012)	Total Stress; Child Domain subscales: Distractibility/Hyperactivity; Adaptability; Reinforces Parent; Demandingness; Mood; Acceptability Parent Domain subscales: Competence; Isolation; Attachment; Health; Role Restriction; Spouse/Parenting Partner Relationship	Normative Sample (USA)	α=.96/.90, .78–.88/.70–.83 (subscales) Child Domain; .96/.93, .75–.87/.70–.86 (subscales) Parent Domain; .98/.95 Total Stress (PSI-4/PSI)	r=.63 Child Domain; .91 Parent Domain; .96 Total Stress (1–3 months; mothers at parenting clinic)	Convergent: r=.85–.99 between PSI and PSI-4; construct validity across multiple languages and clinical and nonclinical populations	N/R	Brosig, et al., 2007a (USA)	N/R
							Golfenshtein et al., 2017 (UK)	N/R
							Majnemer et al., 2006 (Canada)	N/R
							Torowicz et al., 2010 (USA)	α=.80–.87 Subscales ; .91 Total Stress
							Visconti et al., 2002 (USA)	N/R
							Medoff-Cooper et al., 2020 (USA)	N/R
							Goldberg et al., 1991 (Canada)	N/R
							Sarajuuri et al., 2012 (Finland)	N/R
**Parenting Stress Index-Short Form (PSI-SF; PSI SF-4)** (Abidin, 2012)	Parental Distress; Parent-Child Dysfunctional Interaction; Difficult Child; Total Stress	Normative Sample (USA)	α=.90/.87 Parental Distress; .89/.80 Parent-Child Dysfunctional Interaction; .88/.85	r=.85 Parental Distress; .68 Parent-Child Dysfunctional	Convergent: r=.98 PSI-4 and PSI-4-SF; .99 PSI-SF and PSI-4-SF Total Scale	N/R	Carey et al., 2002 (USA) PSI/SF	N/R
							Caris et al., 2016 (USA)	N/R

Instrument	Domains or Scales or Indices	Original Instrument Development Properties					CHD Study Properties	
		Sample	Reliability		Validity		Reference (Country)	Reliability
			Internal Consistency	Test-Retest	Construct	Criterion		
			Difficult Child; .95/.91 Total Stress (PSI-4-SF/)	Interaction; .78 Difficult Child; .84 Total Stress (PSI-SF; 6 months)			Kaugars et al., 2018 (USA)	N/R
							Re et al., 2018 (Australia)	N/R
							Uzark & Jones, 2003 (USA)	N/R
							De Stasio et al., 2019 (Italy)	N/R
**PSI-SF Dutch version** (Brock et al., 1992)	Parental Distress; Parent-Child Dysfunctional Interaction; Difficult Child; Total Stress	N/I	N/I	N/I	N/I	N/I	Jantien Vrijmoet - Wiersma et al., 2009 (The Netherlands)	α=.93 Total Stress
							van der Mheen et al., 2019 (The Netherlands)	N/R
**PSI-SF Thai version** (Not identified)	Parental Distress; Parent-Child Dysfunctional Interaction; Difficult Child; Total Stress	N/I	N/I	N/I	N/I	N/I	Chaisom et al., 2010 (Thailand)	N/R
**PSI-SF Arabic translation** (translated and piloted for CHD study (Ezzat et al., 2016)	Parental Distress; Parent-Child Dysfunctional Interaction; Difficult Child; Total Stress	Mothers of Sequential Infants born with Conotruncal Heart Malformatio	.73 Parental Distress; .75 Parent-Child Dysfunctional Interaction; .79 Difficult Child; .57 Defensive Responding	N/R	N/R	N/R	Ezzat et al., 2016 (Saudi Arabia)	α=.73 Parental Distress; .75 Parent-Child Dysfunctional Interaction; .79 Difficult Child; .87 Total Stress

Instrument	Domains or Scales or Indices	Original Instrument Development Properties					CHD Study Properties	
		Sample	Reliability		Validity		Reference (Country)	Reliability
			Internal Consistency	Test-Retest	Construct	Criterion		
		ns & Age-matched Controls (Egypt)	(removed from analysis); .87 Total Stress					
**PSI-SF Korean version** (Kim, 1997)		N/I	N/I	N/I	N/I	N/I	Lee et al., 2007 (Korea)	α=.90
**PSI-SF Chinese version** (Pearson & Chan, 1993)^		N/I	α=.92 (excluding Life Stress scale)	N/I	N/I	N/I	Chang at al., 2020 (Taiwan)	α=.95
**Swedish Parenthood Stress Questionnaire (SPSQ)** (Östberg et al., 1997)	Incompetence; Role Restriction; Social Isolation; Spouse Relationship Problems; Health Problems; Total Score	Normative Sample of Mothers (Sweden)	α=.75–.82 Incompetence; .68–.79 Role Restriction; .68–.71 Social Isolation; .74–.75 Spouse Relationship Problems; .57–.68 Health Problems; .87–.90 Total Score	r=.85 Incompetence; .83 Role Restriction; .79 Social Isolation; .80 Spouse Relationship Problems; .86 Health Problems; .89 Total Score (Subsample; 30 days)	Convergent: r = .29 EPDS<br><br>Discriminant: *r*=-.46 Social Support Network; -.37 Emotional Support	Concurrent: r=.38/.53 Parenting Stress Index Total Score (Mother/Psychologist)	Morelius et al., 2002 (Sweden)	N/R
**Pediatric Inventory for Parents (PIP)** (Streisand et al., 2001)	Communication; Medical Care; Role Function; Emotional Function; Total Difficulty	Parents of Children in Outpatient Oncology (USA)	α=.80/.82 Communication; .84/.83 Medical Care; .82/.85 Role Function ; .88/.92 Emotional Function; .95/.96	N/R	Convergent: r=.60–.62 STAI State Anxiety; .29–.38 Parenting Stress Index - Short Form	N/R	Caris et al., 2016 (USA)	N/R
							Kaugars at al., 2018 (USA)	N/R
							Poh et al., 2020 (Singapore)	N/R

Instrument	Domains or Scales or Indices	Original Instrument Development Properties					CHD Study Properties	
		Sample	Reliability		Validity		Reference (Country)	Reliability
			Internal Consistency	Test-Retest	Construct	Criterion		
			Total Score (PIP-F/PIP-D)				Bishop et al., 2019 (USA)	α=.97 Total Score
							Jackson et al., 2020 (Australia) PIP-D only	α=.93 PIP-D
							Mussatto et al., 2021 (USA)	N/R
**PIP Korean translation** for study (Choi & Lee, 2021)^	Communication; Medical Care; Role Function; Emotional Function; Total Score	Mothers of Children with CHD	α=.95 PIP-F; .96 PIP-D	N/R	N/R	N/R	Choi &Lee, 2021 (Korea)	α=.95 PIP-F; .96 PIP-D
**PIP-Short Form Dutch version** (Jantien Vrijmoet-Wiersma et al., 2010)	Communication; Medical Care; Role Function; Emotional Function Total Score	Parents of Children with Cancer (The Netherlands)	α=.60/.73 Communication; .85/.84 Medical Care; .88/.91 Role Function; .80/.82 Emotional Function; .94/.95 Total Score (PIP-F/PIP-D)	r=≤.68–≥.87 (14 days)	Convergent: r=.52, .55/.59, .66 State Trait Anxiety Inventory; .54/.51 General Health Questionnaire; .19/.24 Parenting Stress Index – Short Form (Frequency/Difficulty<br><br>Discriminant: between sex, age, treatment, and diagnosis timing	N/R	Jantien Vrijmoet - Wiersma 2009 et al., (The Netherlands)	α=.96 Total Score
**Distress Thermometer for Parents (DT-P)** (Haverman et al., 2013)	Thermometer Score (Overall Distress); Practical Problems; Social Problems; Emotional; Physical; Cognitive; Parenting Problems <2 years; ≥2 years; Total	Outpatient Clinic (The Netherlands)	α=≥.90 All Problem Domains	N/R	Convergent: r=.55–.61; .50–.72; .64 –.76 Hospital Anxiety and Depression Scale (Thermometer Score; Subscales .48; .38–.51 Dutch Parenting Stress	N/R	van der Mheen et al., 2019 (The Netherlands)	N/R

Instrument	Domains or Scales or Indices	Original Instrument Development Properties					CHD Study Properties	
		Sample	Reliability		Validity		Reference (Country)	Reliability
			Internal Consistency	Test-Retest	Construct	Criterion		
	Score (5 or 6 domains); Additional Questions: Enough Support + Need for Referral				Index (Thermometer Score; Subscales)			
**Parent Stress, Study-specific Instrument** (Barsella et al., 2021)	Parent Stress Items	Parents Post-Cardiac Surgery	N/R	N/R	N/R	N/R	Barsella et al., 2021 (USA)	N/R
**Caregiver Task Inventory Chinese version Short Form** (Lee & Mok, 2011)	Learning to Cope with New Role; Providing Care According to Care-Receiver's Needs; Managing Own Emotional Needs; Appraising Supportive Resources; Balancing Caregiving Needs and Own Needs.	Family caregivers	α=.93 Overall; .75 Learning to Cope with New Role; .76 Providing Care According to Care-Receiver's needs; .72 Managing Own Emotional Needs; .67 Appraising Supportive Resources; .86 Balancing Caregiving Needs and Own Needs	N/R	Confirmatory Factor Analysis verified subscales and five factor model	N/R	Zhang et al., 2020 (China)	N/R
**Zarit Caregiver Burden Interview (ZBI) Turkish version** (Inci & Erdem, 2008)	Total Score	N/I	N/I	N/I	N/I	N/I	Bektas et al., 2020 (Turkey)	N/R
**Zarit Caregiver Burden Interview (ZBI) Chinese version** (Wang et al., 2006)	Total Score	N/I	N/I	N/I	N/I	N/I	Zhang et al., 2020 (China)	N/R

Instrument	Domains or Scales or Indices	Original Instrument Development Properties					CHD Study Properties	
		Sample	Reliability		Validity		Reference (Country)	Reliability
			Internal Consistency	Test-Retest	Construct	Criterion		
**The Care of My Child** – CHD modification of Caregiving Burden Scale (Svavarsdottir & McCubbin, 1996)	N/I	N/I	N/I	N/I	N/I	N/I	Svavarsdottir & McCubbin., 1996 (USA)	α=.86–.88 Time Scale; .89– .90 Difficulty Scale
**Quality of Life and Health-Related Quality of Life**								
**36 Item Short-Form Health Survey (SF-36)** (Ware et al., 2000)	Physical Functioning; Role-Physical; Bodily Pain; General Health; Vitality; Social Functioning; Role-Emotional; Mental Health; Reported Health; Transition; Physical Component Summary Score; Mental Component Summary Score	Patients with One or More Chronic Conditions	α=.93 Physical Functioning; .84 Role-Physical; .82 Bodily Pain; .78 General Health; .87 Vitality; .85 Social Functioning; .83 Role-Emotional; .90 Mental Health	r=.43–.90 (Diabetic Population; 6 months)	Physical Functioning scale strongly associated with physical health component (r≥.70) and poorly with mental health component (r≤.30); Mental Health scale strongly associated with mental health component (r≥.70) and poorly with physical health component (r≤.30)	Extensive data available in manual	Bevilacqua et al., 2013 (Italy)	N/R
**SF-36 Turkish version** (Pinar, 2005)	Physical Functioning; Role-Physical; Bodily Pain; General Health; Vitality; Social Functioning; Role-Emotional; Mental Health; Reported Health; Transition; Physical Component Summary Score; Mental	Cancer patients (Turkey)	α=.90 Physical Functioning; .87 Role-Physical; .86 Bodily Pain; .79 General Health; .87 Vitality; .84 Social Functioning; .82 Role-Emotional; .82 Mental Health	ANOVA ICC .93 Physical Functioning; .81 Role-Physical; .90 Bodily Pain; .94 General Health; .86 Vitality; .88 Social	Factor Analysis: presence of 7 factors suggested. Physical functioning accounted for majority of variance (34%). Loading of all items ≥.61 across all subscales.	N/R	Alkan et al. 2017 (Turkey)	N/R

Instrument	Domains or Scales or Indices	Original Instrument Development Properties					CHD Study Properties	
		Sample	Reliability		Validity		Reference (Country)	Reliability
			Internal Consistency	Test-Retest	Construct	Criterion		
	Component Summary Score			Functioning; .87 Role-Emotional; .84 Mental Health (2 weeks)				
**SF-36 German version** (Bullinger, 1995)	Physical Functioning; Role-Physical; Bodily Pain; General Health; Vitality; Social Functioning; Role-Emotional; Mental Health; Reported Health; Transition; Physical Component Summary Score; Mental Component Summary Score	Healthy and Ill Populations (Germany)	α=.88–.92 Physical Functioning; .74–.84 Role-Physical; .72–.86 Bodily Pain; .64 -.76 General Health; .78–.83 Vitality; .73–.85 Social Functioning; .77–.90 Role-Emotional; .82–.87 Mental Health	N/R	Convergent: significant correlation with Nottingham Health Profile measure in psychological and physical domains in student and backpain cohorts<br>Discriminant: clear differences identified between healthy and ill cohorts, particularly in vitality, role functional, and pain scales	N/R	Landolt et al., 2011 (Switzerland)<br>Stoffel et al., 2017 (Switzerland)	α=.71–.93 (all subscales and summary scores)<br>N/R
**SF-36 Persian version** (Montazeri et al., 2009)	Physical Functioning; Role-Physical; Bodily Pain; General Health; Vitality; Social Functioning; Role-Emotional; Mental Health; Reported Health; Transition; Physical Component Summary Score; Mental Component Summary Score	Medical School Staff (Iran)	α=.90 Physical Functioning; .85 Role-Physical; .83 Bodily Pain; .71 General Health; .65 Vitality; .77 Social Functioning; .84 Role-Emotional; .82 Mental Health	N/R	Convergent: significant correlation between Physical Functioning, Physical Component Score and age (.27, .18); and education level, Physical Component Score, and role limitations (.22, .20)	N/R	Edraki et al., 2014 (Iran)	α=.91 Total Score

Instrument	Domains or Scales or Indices	Original Instrument Development Properties					CHD Study Properties	
		Sample	Reliability		Validity		Reference (Country)	Reliability
			Internal Consistency	Test-Retest	Construct	Criterion		
					Correlation with reported physical health in French and US studies			
**SF-36 Dutch version** (Aaronson et al., 1998)		Licit and Illicit Drug, General Community, Migraine and Cancer Populations	α=.90–.93 Physical Functioning; .86–.90 Role-Physical; .88–.92 Bodily Pain; .70–.81 General Health; .78–.83 Vitality; .66–.83 Social Functioning; .83–.87 Role-Emotional; .82–.87 Mental Health	N/R	Known groups comparison: hypothesized differences related to age, gender, and presence of a chronic condition were predominantly detected	N/R	van der Mheen et al., 2019 (The Netherlands)	N/R
**12-Item Short-Form Health Survey (SF-12) Italian version** (Kodraliu et al., 2001)	Physical Functioning; Role-Physical; Bodily Pain; General Health; Vitality; Social Functioning; Role-Emotional; Mental Health; Physical Component Summary Score; Mental Component Summary Score	General Population and Patient Groups (Italy)	N/R	N/R	Predicted correlations between mental health items and summary scores, and physical health items and summary scores on SF-12 across multiple samples	Higher Physical Summary Scores in representative population (48.5) than Asthma cohort (40.4)	Simeone et al., 2018 (Italy)	N/R
**Ulm Quality of Life Inventory for Parents (ULQIE)** (Goldbeck & Storck, 2002; Goldbeck & Melches, 2006)^	Physical and Daily Functioning; Satisfaction with Support from the Family; Emotional Stability; Self-Development; Wellbeing Total Scale	Parents of Children with Cancer, Hematologic al Disease and Diabetes	α=.74–.88 across Subscales; .91 Total Scale	N/I	N/I	N/I	Goldbeck & Melches, 2006 (Germany)	N/R
							Medoff-Cooper et al., 2020 (USA)	N/R

Instrument	Domains or Scales or Indices	Original Instrument Development Properties						CHD Study Properties	
		Sample	Reliability		Validity			Reference (Country)	Reliability
			Internal Consistency	Test-Retest	Construct	Criterion			
**Perceived Quality of Life Scale (PQOL)** (Patrick et al., 2000)	Total Score	Adults with and without Chronic Conditions (USA)	N/R	N/R	N/R	N/R		Mussatto et al., 2021 (USA)	N/R
**World Health Organization Quality of Life (WHOQOL-100)** (Kuyken et al., 1995; Power et al., 1998) #Modified	Physical Health; Psychological Functions; Level of Independence; Social Relationships; Environment; Spirituality /Religion/Personal Beliefs	Multiple Internationa l Field Centers	α=.84 Overall; .76/.81/.81/.71/.86/ .89 Physical Health/ Psychological/Level of Independence/ Social Relationships/ Environment/ Spiritual	r=.78 Overall; .74/.83/.81/ .81/.90/.80 Physical Health/ Psychological/ Level of Independence / Social Relationships/ Environment/ Spiritual	Discriminant: t=29.9 Overall;.35.3/25/48.3/ 20.4/14.8/9.2 Physical Health/ Psychological /Level of Independence/ Social Relationships/ Environment/Spiritual between well and ill subjects	N/R		Hussein & Authman, 2013 (Iran)	α=.91
**World Health Organization Quality of Life Scale BREF (WHOQOL-BREF)** (Skevington et al., 2004; Whoqol, 1998)	Physical Health; Psychological; Social Relationships; Environment	Multiple Internationa l Field Centers	α=.80–.82 Physical Health; .75-.77 Psychological; .66–.69 Social Relationships; .80 Environment	r=.56–.84 Overall; .66/.72/.76/ .87 Physical Health/ Psychological /Social Relationships /Environment (2–8 weeks)	Discriminant: t=24.2–36.4 Physical Health; 10.6–24.0 Psychological; 6.9–16.2 Social Relationships; 2.8–21.1 Environment comparable with WHOQOL-100 in discriminating between ill and well groups	r=.90 with WHOQOL-100 domain scores		Zhang et al., 2021 (China)	N/R
**WHOQOL-BREF - Turkish version** (Eser et al., 1999; Bektas et al., 2020)^		N/I	α=.83 Physical Domain; .53 Social Domain; .66 Spiritual Domain; .73 Environment	r = .57-.81 (Time difference unknown)	N/I	N/I		Bektas et al., 2020 (Turkey)	N/R

Instrument	Domains or Scales or Indices	Original Instrument Development Properties					CHD Study Properties	
		Sample	Reliability		Validity		Reference (Country)	Reliability
			Internal Consistency	Test-Retest	Construct	Criterion		
**WHOQOL-BREF - Sri Lankan/ Sinhala language** (Somasiri & Gunawardana, 1995)		N/I	N/I	N/I	N/I	N/I	Warnakulasooriya & Kasturiaratchi, 2020 (Sri Lanka)	N/R
**Linear Analogue Scale Quality of Life** (Williams, 1990)	N/A	Adults with CHD	N/A	N/I	N/I	N/R	Levert et al., 2017 (The Netherlands)	N/R
**Study-Specific Measures of Parental, Sibling, and Child Quality of Life** (Azhar et al., 2016)	5-part questionnaire Part 3 – Parent Quality of Life; Part 4 – Sibling Quality of Life	Parents of Children with CHD	α=.76 Child with CHD component only	N/R	N/R	N/R	Azhar et al., 2016 (Saudi Arabia)	N/R
**Pediatric Quality of Life (PedsQL) Family Impact Module** (Varni et al., 2004)	Parent Health-Related Quality of Life: Physical Functioning; Emotional Functioning; Social Functioning; Cognitive Functioning; Communication; Worry; Family Functioning; Daily Activities; Family Relationships	Medically Fragile Children Residing in a Care Facility and at Home (USA)	α=.96 Parent Health-Related Quality of Life Summary Score; .90 Family Functioning Summary Score; .97 Total Score	N/R	Statistically significant difference across 7/11 and medium to large effect size across 10/11 scales between 2 pediatric chronically ill populations (small sample size noted)	N/R	Denniss et al., 2019 (Australia)	α=.93 Physical Functioning; .92 Emotional Functioning; .91 Social Functioning; .96 Cognitive Functioning; .83 Communication; .82 Worry; .94 Daily Activities; .97 Family Relationships; .97 Total Score
							Eagleson et al., 2013 (Australia)	N/R

Instrument	Domains or Scales or Indices	Original Instrument Development Properties					CHD Study Properties	
		Sample	Reliability		Validity		Reference (Country)	Reliability
			Internal Consistency	Test-Retest	Construct	Criterion		
							Kaugars et al., 2018 (USA)	N/R
							Hancock et al., 2018 (USA)	N/R
							Lee et al., 2020 (Canada)	N/R
**Family Member Well-being Index (FMWB)** (McCubbin, & Patterson, 1983)	Total Score	N/I	α=.85	N/R	N/I	N/R	Mussatto et al., 2021 (USA)	N/R
**Life Satisfaction Questionnaire (LiSat-11)** (Fugl-Meyer et al., 2002)		Community Sample (Sweden)	α=.85	N/R	N/R	N/R	Bratt et al., 2019 (Sweden)	N/R
**Family Impact or Functioning**								
**Psychosocial Assessment Tool (PAT 2.0)** (Pai et al., 2008)	Family Structure and Resources; Family Social Support; Family Problems; Parent Stress Reactions; Family Beliefs; Child Problems; Sibling Problems	Parents of Children Newly Diagnosed with Cancer (USA)	α=.62–.81 Subscales; .81 Total Score	r=.78/.87 (Mothers/Fathers)	Convergent: r=.45/.38 for mothers scores with physician/nurse reports<br><br>Discriminant: r=-.10/-.05 for mothers/fathers with physician rated treatment intensity	Concurrent: r=.57/.30 Acute Stress Disorder Scale; .41/.28 State Trait Anxiety Inventory-State; .65/.51 Behavioral Assessment for Children Scale; .40/.31 FES-conflict (mother/ father)	Hearps et al., 2014 (Australia) (Modified/ Extended Version)	N/R

Instrument	Domains or Scales or Indices	Original Instrument Development Properties					CHD Study Properties	
		Sample	Reliability		Validity		Reference (Country)	Reliability
			Internal Consistency	Test-Retest	Construct	Criterion		
**Family Assessment Device (FAD)** (Epstein, & et al., 1983; Miller, et al., 1985)	Problem Solving; Communication Roles; Affective Responsiveness; Affective Involvement; Behavior Control; General Functioning	1) Families of Children in Psychiatric Day Hospital, Patients in a Stroke Rehab Unit; 2) Students in Advanced Psychology Course, Adult Psychiatric Inpatients; 3) 45 Nonclinical individuals currently living with one or more family members	α=.72–.83 Subscales; .92 General Functioning (Sample 1)	r=.66–.76 (Sample 3)	Discriminant: Scores consistent with clinician ratings of healthy and unhealthy families in psychiatric patient cohort (Sample 2)	Concurrent: scales closely correlated with Family Unit Inventory and FACES II measures (Sample 3)	Menahem et al., 2008 (Australia)	N/R
							Mussatto et al., 2021 (USA) General Functioning subscale only	N/R
							van der Mheen et al., 2019 (The Netherlands) General Functioning subscale only	N/R
**FAD Turkish version** (Bulut, 1990)	Problem Solving; Communication Roles; Affective Responsiveness; Affective Involvement; Behavior Control; General Functioning	N/I	N/I	N/I	N/I	N/I	Alkan et al., 2017 (Turkey)	N/R
**Family Environment Scale (FES)** (Moos, 1990; Roosa & Beals, 1990)^	3 Dimensions; 10 Subscales<br>Family Relationship Index: Cohesion; Expressiveness; Conflict<br>Personal Growth: Independence; Achievement Orientation; Intellectual-Cultural	Community Sample (USA)	α=.61–.78 Total subscales; .78 Cohesion; .69 Expressiveness; .75 Conflict; .76 Organization; .67 Control	r=.73–.86; .66– .78 for 5 subscales (8 week; 4 months)	Convergent: positively correlations with dyadic and marital adjustment measures; conflict scale with family arguments; organization and control scales with reliance on	Concurrent: associations with adaptation in varying populations including pregnancy and parenthood and chronic	Davis et al., 1998 (USA)	N/R
							Doherty et al., 2009 (UK)	N/R

Instrument	Domains or Scales or Indices	Original Instrument Development Properties					CHD Study Properties	
		Sample	Reliability		Validity		Reference (Country)	Reliability
			Internal Consistency	Test-Retest	Construct	Criterion		
	Orientation; Active-Recreational Orientation; Moral Religious Emphasis System Maintenance and Change: Organization; Control				predictable family routines	childhood illness<br><br>Predictive: associations with treatment outcomes including alcoholism, psychiatric and medical disorders		
**Pediatric Quality of Life (PedsQL) Family Impact Module** (Varni et al., 2004) – As above								
**Impact on Family Scale (IOFS)** (Stein & Riessman, 1980; Stein, 1985)	Financial; Familial/Social; Personal Strain; Mastery; Total Score (Sibling scale removed in development)	Mothers of Children with Chronic Illness (English and Hispanic speaking)	α=.72 Financial; .86 Familial/Social; .81 Personal Strain; .60 Mastery; .88 Total Score	N/R	High Total Scores correlate with wide range of factors including low education and family income, lack of social support, poor child psychological adjustment, and increased maternal psychiatric symptoms	N/R	Almesned et al., 2013 (Saudi Arabia)	N/R
							Brosig, et al., 2007a (USA) includes sibling scale	N/R
							Cantwell-Bartl & Tibballs, 2017 (Australia)	N/R
							Mussatto et al., 2021 (USA)	N/R

Instrument	Domains or Scales or Indices	Original Instrument Development Properties					CHD Study Properties	
		Sample	Reliability		Validity		Reference (Country)	Reliability
			Internal Consistency	Test-Retest	Construct	Criterion		
**Impact on Family Scale Revised (IOFS-R)** (Stein & Jessop, 2003)	Total Score + Optional Scales	Parents of Children with Chronic Illnesses	α=.83–.89 Total Score; .68–.79 Financial Subscale; Sibling Subscale – not reported	N/R	Construct: as for IOFS	Concurrent: r=.97 revised and original IOFS Total Score across 3 cohorts	Garcia et al., 2016 (USA) +financial and sibling subscales	N/R
							Landolt et al., 2011 (Switzerland)	α=.88/.90 Total Score (Mothers/ Fathers)
**IOFS-R German version** (Ravens-Sieberer et al., 2001)		N/I	N/I	N/I	N/I	N/I	Stoffel et al., 2017 (Switzerland)	N/R
**Family Inventory of Life Events and Changes (FILE)** (McCubbin, Patterson et al.,1983)	Intrafamily Strains; Marital Strains; Pregnancy & Childbearing Strains; Finance and Business Strains; Work-Family Transitions and Strains; Illness & Family "Care" Strains"; Losses; Transitions "In and Out"; Family Legal Violations	Families of a Chronically Ill Child (Myelomeningocele or Cerebral Palsy)	α=.72 Total Scale	r=.80 Total Scale (125 adults; 4–5 weeks)	Convergent & Discriminant: r=.23/.24/.16/.14 between FILE Total Life Changes and Family Environment Scale Conflict/ Cohesion/ Independence/ Organization Indices	Predictive: Negative correlation between pile-up family life changes across multiple categories and Total Life Changes and pulmonary functioning in children with Cerebral Palsy	Svavarsdottir & McCubbin., 1996 (USA)	N/R
**Family Hardiness Index (FHI)** (McCubbin, et al., 1986)	Commitment, Challenge, Control Subscales; Total Score	N/I	α.82 (overall); .81/.80/.65 (Commitment, Challenge, Control subscales)	r=.86 (time not specified)	N/R	r=.22 (FACES-II); .23 (Family Time and Routines Index); .11-.20 (Quality of Family Life adapted)	Mussatto et al., 2021 (USA)	N/R

Instrument	Domains or Scales or Indices	Original Instrument Development Properties						CHD Study Properties	
		Sample	Reliability		Validity			Reference (Country)	Reliability
			Internal Consistency	Test-Retest	Construct	Criterion			
**Family Illness Accommodation Questionnaire, Study-Specific Instrument** (Janus & Goldberg, 1997)	Nil	Parents of Children with CHD	α=.85 Total	N/R	N/R	N/R		Janus & Goldberg, 1997 (Canada)	α=.85 Total
**Financial Strain/Economic Hardship Instrument** (Vinokur & Caplan, 1987)	N/R	Unemployed Vietnam War Veterans, and non-Veterans of same generation (USA)	α=.85	N/R	N/R	N/R		Lisanti et al., 2021b (USA)	N/R
**Coping**									
**Coping Inventory for Stressful Situations (CISS)** (Endler & Parker, 1994)^	Task-Oriented; Emotion-Oriented; Avoidance-Oriented	Under-graduate Students (Canada)	α=high 80–low 90	r=.51–.73 (6 weeks)	Convergent and Divergent correlation with Ways of Coping Questionnaire "theoretically meaningful"	N/R		Diffin et al., 2016 (Australia)	N/R
**Coping Inventory for Stressful Situations Korean translation** for study (Ahn & Lee, 2014)	Task-Oriented; Emotion-Oriented; Avoidance-Oriented	Study-Specific Parents and Adolescents with CHD from Outpatient Pediatric Cardiology Clinic (Korea)	α=.73 Task-Oriented; .74 Emotion-Oriented; .74 Avoidance-Oriented	N/R	N/R	N/R		Ahn et al., 2014 (Korea)	α=.73 Task-Oriented; .74 Emotion-Oriented; .74 Avoidance-Oriented

Instrument	Domains or Scales or Indices	Original Instrument Development Properties					CHD Study Properties	
		Sample	Reliability		Validity		Reference (Country)	Reliability
			Internal Consistency	Test-Retest	Construct	Criterion		
**Coping Health Inventory for Parents (CHIP)** (McCubbin et al., 1981; McCubbin, McCubbin, et al., 1983)	Pattern 1: Maintaining Family Integration, Cooperation and Optimistic Definition of the Situation; Pattern 2: Maintaining Social Support, Self-Esteem, and Psychological Stability; Pattern 3: Understanding the Medical Situation through Communication with Other Parents and Medical Staff	Parents of Children with Cystic Fibrosis (USA)	α=.79 Pattern 1; .79 Pattern 2; .71 Pattern 3	N/R	Convergent: r=.21; .19 for Coping Patterns I and III with Family Environment Scale (FES) Family Cohesiveness; .19 for Pattern II FES with Family Expressiveness for mothers; .36 for Coping Pattern I with FES Family Cohesiveness and .32 with Family Organization; .22 for Pattern III with FES Family Organization and .19 with Family Control for fathers in Cystic Fibrosis cohort<br><br>Discriminant: reported in high vs. low conflict cohort of families with a child with Cerebral Palsy	N/R	Sira et al., 2014 (USA)<br><br>Svavarsdottir & McCubbin, 1996 (USA)<br><br>Poh et al. 2020 (Singapore)<br><br>Miller et al., 2021 (USA)<br><br>Mussatto et al., 2021 (USA)	N/R<br><br>N/R<br><br>N/R<br><br>N/R<br><br>N/R
**COPE Inventory** (Carver et al., 1989)	Active Coping; Planning; Suppression of Competing Activities; Restraint Coping; Seeking Social Support – Instrumental; Seeking Social Support – Emotional; Positive Reinterpretation &	Under-graduate Students (USA)	α =.62 Active Coping; .80 Planning; .68 Suppression of Competing Activities; .72 Restraint Coping; .75 Seeking Social Support – Instrumental; .85 Seeking Social	r=.56 Active Coping; .63 Planning; .46 Suppression of Competing Activities; .51 Restraint Coping; .64 Seeking Social	Convergent: r=.32/ .25 for Active Coping /Planning with Optimism and .27/.22 with Self-Esteem<br><br>Discriminant: Not strong correlations with	N/R	Doherty et al., 2009 – Situational (UK)<br><br>McCusker et al., 2010 Partial; Situational (UK)	N/R<br><br>N/R

Instrument	Domains or Scales or Indices	Original Instrument Development Properties					CHD Study Properties	
		Sample	Reliability		Validity		Reference (Country)	Reliability
			Internal Consistency	Test-Retest	Construct	Criterion		
	Growth; Acceptance; Turning to Religion; Focus on & Venting of Emotions; Denial; Behavioral Disengagement; Mental Disengagement; Alcohol-Drug Disengagement		Support – Emotional; .68 Positive Reinterpretation & Growth; .65 Acceptance; .92 Turning to Religion; .77 Focus on & Venting of Emotions; .71 Denial; .63 Behavioral Disengagement; .45 Mental Disengagement	Support – Instrumental; .77 Seeking Social Support – Emotional; .48 Positive Reinterpretation & Growth; .63 Acceptance; .86 Turning to Religion; .69 Focus on & Venting of Emotions; .54 Denial; .66 Behavioral Disengagement .58 Mental Disengagement; .57 Alcohol-Drug Disengagement (8 weeks)	personality variables, social desirability scale. And lack of relation to other coping styles		Rychik et al., 2013 – not specified (USA)	N/R
**Brief COPE** (Carver, 1997)	Active Coping; Planning; Positive Reframing; Acceptance; Humor; Religion; Using Emotional Support; Using Instrumental Support; Self-Distraction; Denial; Venting; Substance; Behavioral	Community Sample Impacted by Hurricane (USA)	α=.68 Active Coping; .73 Planning; .64 Positive Reframing; .57 Acceptance; .73 Humor; .82 Religion; .71 Using Emotional Support; .64 Using Instrumental Support; .71 Self-	N/R	N/R	N/R	Hancock et al., 2018 (USA)	N/R

Instrument	Domains or Scales or Indices	Original Instrument Development Properties						CHD Study Properties	
		Sample	Reliability		Validity			Reference (Country)	Reliability
			Internal Consistency	Test-Retest	Construct	Criterion			
	Disengagement; Self-Blame		Distraction; .54 Denial; .50 Venting; .90 Substance; .65 Behavioral Disengagement; .69 Self-Blame						
**Utrecht Coping List (UCL)** (Tielemans et al., 2014)^	Passive Reactions; Active Problem Solving; Palliative Reactions; Seeking Social Support; Avoidance; Expression of Emotions; Reassuring Thoughts	N/I	α=.43–.89	r=37–.85	N/I	N/I		Spijkerboer et al., 2007 (The Netherlands)	N/R
								Utens et al., 2000 (The Netherlands)	N/R
**Ways of Coping Questionnaire (WCQ)** (Folkman & Lazarus, 1985)	Confrontive Coping; Distancing; Self-Controlling; Seeking Social Support; Accepting Responsibility; Escape-Avoidance; Planful Problem Solving; Positive Reappraisal	Community Sample (USA)	α=.56–.85	N/R	N/R	N/R		Davis et al., 1998 (USA) (overall child illness specified)	α=.79 Palliative (Emotion-focused) Coping; .84 Adaptive (Problem-focused) Coping
**Ways of Coping Questionnaire (WCQ) Hebrew version** – modified (Solomon et al., 1988)	Problem-Focused Coping; Emotion Focused Coping; Seeking Social Support; Distancing	Combat Survivors (Israel)	α=.88, .90 Problem-Focused Coping; .82, .88; Emotion-Focused Coping; .86, .86 Seeking Social Support; .74, .76 Distancing (Time 1, Time 2)	r=.38–.58 across all scales (1 year)	N/R	N/R		Berant et al., 2001 (Modified; tasks of motherhood specified; Israel)	α=.72–.87 Items; .71-.89 across 3 groups CHD and non-CHD
								Berant et al., 2003 (Modified; tasks of motherhood	α=.72–.82 across 4 categories/3 groups CHD and non-CHD

Instrument	Domains or Scales or Indices	Original Instrument Development Properties					CHD Study Properties	
		Sample	Reliability		Validity		Reference (Country)	Reliability
			Internal Consistency	Test-Retest	Construct	Criterion		
							specified; Israel)	
**Coping Scale for Adults, Second Edition-Short Form** (Jackson et al., 2020)	Total Score	N/I	N/I	N/I	N/I	N/I	Jackson et al., 2020 (Australia)	N/R
**Coping Self-Efficacy Scale** developed for study (Jackson et al., 2020)	Total Score (mean)	Health Professionals undertaking training for "Cardiac Blues" post-acute cardiac event/CHD Study	N/R	N/R	N/R	N/R	Jackson et al., 2020 (Australia)	N/R
**Coping Self-Efficacy Scale (CSES) Revised and translated to Korean** for study (Choi & Lee, 2021)	Problem Focused Coping; Stop Unpleasant Emotions and Thoughts; Get Support from Friends and Family	Mothers of Children with CHD	α=.92	N/R	N/R	N/R	Choi & Lee, 2021 (Korea)	α=.92
**Response to Stress Questionnaire (RSQ-CHD)** (Jackson et al., 2017)	Total Score	Adolescent, Emerging Adult and Young adult with CHD	α=.87 (stressor items)	N/R	N/R	N/R	Roberts et al., 2021 (Canada)	N/R

Instrument	Domains or Scales or Indices	Original Instrument Development Properties					CHD Study Properties	
		Sample	Reliability		Validity		Reference (Country)	Reliability
			Internal Consistency	Test-Retest	Construct	Criterion		
**Cognitive Appraisal Scale** Hebrew version (Berant et al., 2001; Berant et al., 2003) Modified	Cognitive Appraisal of Stress and Coping (Tailored to Motherhood)	N/I	N/I	N/I	N/I	N/I	Berant et al., 2001<br><br>Berant et al., 2003	α = .77-.85<br><br>α = 76-.87
**Spiritual Insight and Behavioral Scale** (Sira et al., 2014) Modified	Self-awareness; Perception; Use of Spirituality in Coping	Child Life Specialists (USA)	N/I	N/I	N/I	N/I	Sira et al., 2014 (USA)	α=.85
**Stress Appraisal**								
**Hassles and Uplifts Scale – Combined (HSUP)** (Lazarus & Folkman, 1989)	Hassles; Uplifts	White Middle-Class Adults (USA)	N/R	r=.79 for Daily Hassles Scale Form (across 9 months)	Convergent: r=-.42–.85 intraindividual correlation with same-day illness symptoms; -.70–.44 same-day emotional symptoms (Combined Scales); .36 for women and .02 for men with life event scores (Daily Hassles Scale); .34 with Bradburn negative affect scores; .50–.90 with Hopkins Symptoms Checklist (Daily Hassles Scale frequency scores)	N/R	Davis et al., 1998 (USA) (Hassles only)	N/R
**Life Experiences Survey (LES)**	Life Change Scores: Positive; Negative; Total	College Students (USA)	N/R	r=.19 and .53 for Positive Change	Convergent: r=.46 and .29 (Negative Change); .37 and .24 (Total	N/R	Hoehn et al., 2004 (USA)	N/R

Instrument	Domains or Scales or Indices	Original Instrument Development Properties						CHD Study Properties	
		Sample	Reliability		Validity			Reference (Country)	Reliability
			Internal Consistency	Test-Retest	Construct	Criterion			
(Sarason et al., 1978)				Score; α=.56 and .88 Negative Change; .63 and .74 Total Change Score (5–6 weeks)	Change Score) with State and Trait Anxiety on STAI; -.40 - -.21 with academic achievement (Positive, Negative, Total Change Score); .24 with Beck Depression Inventory and .32 with Locus of Control Scale (Negative change Score)				
**Social Readjustment Rating Scale (SRRS)** (Holmes & Rahe, 1967; Rahe et al., 1970)^	N/A	Convenience Sample (USA)	N/R	N/R	N/R	Predictive: r=.12 between illness scores and life change scores		Mussatto et al., 2021 (USA)	N/R
**Parent Stressor Scale: Infant Hospitalization (PSS:IH)** (Miles & Brunssen, 2003)	Parental Role Alteration; Infant Appearance/ Behavior; Sights and Sounds; Total Score	Fragile Hospitalized Infants - Term and Preterm with Chronic Illness (USA)	α=.76/.82 Parental Role Alteration; .79/.87 Infant Appearance/Behavior ; .78/.75 Sights and Sounds; .87/.90 Total Score (Mothers/ Fathers)	r=.45 Parental Role Alteration; .62 Infant Appearance/ Behavior; .59 Sights and Sounds; .61 Total Score (2 weeks)	Convergent: r=.33–.45, All subscales and total score significantly correlated with maternal worry of child's health<br><br>Discriminant: Greater stress reported in Black mothers and more recently hospitalized infants on Sights and	Predictive: Total scores during hospitalization significantly related to maternal depressive symptoms post discharge on Center for Epidemiologic Studies		Lisanti et al., 2017 (USA)<br><br>Lisanti et al., 2021a (USA)	α=.81 Parental Role Alteration; .83 Infant Appearance/ Behavior; .81 Sights and Sounds; .91 Total Score<br><br>α=.92 Parental Role Alteration; .94 Infant Appearance/ Behavior; .91 Sights and Sounds; .96 Total Score

Instrument	Domains or Scales or Indices	Original Instrument Development Properties					CHD Study Properties	
		Sample	Reliability		Validity		Reference (Country)	Reliability
			Internal Consistency	Test-Retest	Construct	Criterion		
					Sounds subscale (F [3.57] =7.0	Depression scale	Lisanti et al., 2021b	N/R
							Franck et al., 2010 (UK) (Modified)	N/R
**Parental Stressor Scale: Neonatal Intensive Care Unit (PSS:NICU)** (Miles et al., 1993)	Infant Behavior and Appearance; Parental Role Alteration; Sights and Sounds	Parents of Premature Infants in NICU & ICU (USA & Canada)	α=.92/.83 Infant Behavior and Appearance; .90/.83 Parental Role Alterations; .80/.73 Sights and Sounds; .94/.89 Total Score (stress Occurrence Level/Overall Stress Level Metrics)	N/R	r=.44/.41 Infant Behavior and Appearance; .44/.40 Parental Role Alternation; .20/.20 Sights and Sounds; .45/.45 Total Score (stress Occurrence Level/Overall Stress Level Metrics) for anxiety (STAI)	N/R	Diffin et al., 2016	N/R
**Neonatal Unit Parental Stressor Scale (NUPS)** (Reid et al., 2007)	Social/Practical Stress; Illness and Treatments Stress; Role and Relationship with Infant Stress; Total Score	Parents in NICU (UK)	α=.89–.92 Social/Practical Stress; .91–.93 Illness and Treatments Stress; .86–.90 Role and Relationship with Infant Stress; .87–.92 Total Score (Across Women/Men at Time 1 & 2)	N/R	r.32–.44/.35–.40 for anxiety/depression (HADS) across scales and overall; -.01–.23 FAD-GF across scales	N/R	Callahan et al, 2019 (USA)	N/R
**Feeling of Tension Questionnaire** (Campbell et al., 1986; Campbell et al., 1992)	N/R	Children with CHD undergoing Cardiac Catheter (USA)	N/R	N/R	N/R	N/R	Campbell et al., 1986 (USA)	N/R
							Campbell et al., 1992 (USA)	N/R

Instrument	Domains or Scales or Indices	Original Instrument Development Properties					CHD Study Properties	
		Sample	Reliability		Validity		Reference (Country)	Reliability
			Internal Consistency	Test-Retest	Construct	Criterion		
**Stress Awareness, Study-Specific Instrument** (Rahimianfar et al., 2015)<br>**Response to Stress Questionnaire (RSQ-CHD)** As above<br>**Cognitive Appraisal Scale** Hebrew version As above	N/I	Mothers of Children with CHD Post Cardiac Surgery	N/R	N/R	N/R	N/R	Rahimianfar et al., 2015 (Iran)	N/R
**Couple Relationship Satisfaction or Strain**								
**Dyadic Adjustment Scale (DAS)** (Spanier, 1976)	Dyadic Satisfaction; Dyadic Cohesion; Dyadic Consensus; Affectional Expression	White Married People	α=.96 Total Scale;.90 Dyadic Satisfaction; .94 Dyadic Cohesion; .86 Dyadic Consensus; .73 Affectional Expression	N/R	Convergent: r=.86/.88 correlation with Lock-Wallace Marital Adjustment Scale in married/divorced sample	Concurrent: Correlation with marital status – mean total scores married=114.8 ;divorced=70.7	Rychik et. 2013 (USA)<br>Bratt et al., 2019 (Sweden)<br>Lisanti et al. 2021b (USA)	N/R<br>N/R<br>N/R
**Evaluating and Nurturing Relationship Issues Communication and Happiness Scale (ENRICH) Shortened Hebrew version**	Idealistic Distortion; Marital Satisfaction; Personality Issues; Communication; Conflict Resolution; Financial Management; Leisure Activities; Sexual Relationship; Children and Parenting; Family and	N/I	N/I	N/I	N/I	N/I	Berant et al., 2003 (Israel)	α=.75–.89 Subscales; .91 Total

Instrument	Domains or Scales or Indices	Original Instrument Development Properties					CHD Study Properties	
		Sample	Reliability		Validity		Reference (Country)	Reliability
			Internal Consistency	Test-Retest	Construct	Criterion		
(Not identified)	Friends; Equalitarian Roles; Religious Orientation; Marital Cohesion; Marital Change							
**ENRICH Finnish translation** and expanded for study (Riikonen et al., 2019)^	Idealistic Distortion; Marital Satisfaction; Personality Issues; Communication; Conflict Resolution; Financial Management; Leisure Activities; Sexual Relationship; Children and Parenting; Family and Friends; Role Relationships; Spiritual Beliefs	Parents of Children with CHD	α=.85 Marital Satisfaction; 0.85 Idealistic Distortion	N/I	N/I	N/I	Riikonen et al., 2019 (Finland)	α=.85 Marital Satisfaction; .85 Idealistic Distortion
**Sibling Psychosocial Outcomes**								
**Sibling Perception Questionnaire (SPQ)** (Lobato & Kao, 2002; Sahler & Carpenter, 1989) Adapted version	Interpersonal Relationships; Intrapersonal Responses; Communication; Fear of Disease	Siblings of Children with Cancer	α=.65–.86 Subscales; .79/.74 Composite Negative Adjustment Scale Siblings/Parents	N/R	N/R	N/R	Caris et al., 2018 (USA)	N/R
**Perception of Effect on Sibling Scale** (Breslau et al., 1981)	Total Score	Families of Pediatric Patients	α=.75 Total Score	N/R	N/R	N/R	Janus & Goldberg, 1997 (Canada)	α=.88; No correlation with parent reported CBCL

Instrument	Domains or Scales or Indices	Original Instrument Development Properties					CHD Study Properties	
		Sample	Reliability		Validity		Reference (Country)	Reliability
			Internal Consistency	Test-Retest	Construct	Criterion		
**Sibling Relationship Questionnaire (SRQ) Korean version** (Moon et al., 2021)^	Warmth/Closeness; Relative Status/Power; Conflict; Rivalry	N/I	α=.93 Warmth/ Closeness; .81 Relative Status/ Power; .85 Conflict; .77 Rivalry	N/I	N/I	N/I	Moon et al., 2021 (Korea)	α=.96 Warmth/ Closeness; .73 Relative Status /Power; .92 Conflict; .65 Rivalry
**Pediatric Quality of Life (PedsQL) Generic Core Scales Urdu translation** for study (Ladak et al., 2019)	Physical Health; Psychosocial Health; Emotional Health; Social Functioning; School Functioning<br>Total Score; Physical Health Summary Score; Psychosocial Health Summary Score	Children with CHD and their Siblings (Pakistan)	Not reported separately	N/R	N/R	N/R	Ladak et al., 2019 (Pakistan)	α=0.83–0.94 across PedsQL Generic Core Scales, Cognitive Functioning Scale, and Cardiac Module (used for child with CHD)
**Pediatric Quality of Life (PedsQL) Cognitive Functioning Urdu translation** for study (Ladak et al., 2019)	Cognitive Functioning	Children with CHD and their Siblings (Pakistan)	Not reported separately	N/R	N/R	N/R	Ladak et al., 2019 (Pakistan)	α=0.83–0.94 across PedsQL Generic Core Scales, Cognitive Functioning Scale, and Cardiac Module (used for child with CHD)

Note: N/A: Not Applicable; N/R: Not Reported; N/I: Not Identified/Available; *English version data presented; #Full instrument data presented; ^Includes secondary sources.

References

Aaronson, N. K., Muller, M., Cohen, P. D. A., Essink-Bot, M.-L., Fekkes, M., Sanderman, R., Sprangers., M.A.G., te Velde, A., & Verrips, E. (1998). Translation, validation, and norming of the Dutch language version of the SF-36 Health Survey in community and chronic disease populations. *Journal of Clinical Epidemiology, 51*, 1055-1068.

Abell, N. (1991). The Index of clinical stress: A brief measure of subjective stress for practice and research. *Social Work Research Abstracts, 27*(2), 12-15.

Abidin, R. R. (2012). *Parenting Stress Index (4th ed).* PAR.

Aguilar García-Iturrospe, E. J., Hidalgo Montesinos, M. D., Cano García, R., López Manzano, J. C. (1995). Estudio prospectivo de la desesperanza en pacientes psicóticos de inicio: características psicométricas de la escala de desesperanza de Beck en este grupo [Prospective study of hopelessness in first-episode psychotic patients: Psychometric characteristics of the Beck Hopelessness Scale in this group]. *Anales de Psiquiatría, 11*(4), 121–125.

Ahn, J. A., Lee, S., & Choi, J. Y. (2014). Comparison of coping strategy and disease knowledge in dyads of parents and their adolescent with congenital heart disease. *Journal of Cardiovascular Nursing, 29*, 508-516.

Alkan, F., Sertcelik, T., Yalın Sapmaz, S., Eser, E., & Coskun, S. (2017). Responses of mothers of children with CHD: Quality of life, anxiety and depression, parental attitudes, family functionality. *Cardiology in the Young, 27*, 1748-1754.

Almesned, S., Al-Akhfash, A., & Al Mesned, A. (2013). Social impact on families of children with complex congenital heart disease. *Annals of Saudi Medicine, 33*(2), 140-143.

Antony, M. M., Bieling, P. J., Cox, B. J., Enns, M. W., & Swinson, R. P. (1998). Psychometric properties of the 42-Item and 21-Item versions of the Depression Anxiety Stress Scales in clinical groups and a community sample. *Psychological Assessment, 10*, 176-181.

Arrindell, W. A., & Ettema, J. H. M. (1986). *Manual for a Multidimensional Psychopathology Indicator (Handleiding bij een multidimensionele psychopathologie-indicator)*. Lisse: Swets & Zeitlinger.

Awaad, M. I., & Darahim, K.E. (2015). Anxiety and depression in the caregivers of children with congenital heart disease: Prevalence and predictors. *Middle East Current Psychiatry, 22*, 179-185.

Aydemir O, G. T., Kuey L & Kültür S. (1997). Hastane anksiyete ve depresyon ölçeği Türkçe formunun geçerlilik ve guvenilirliği. *Turk Psikiyatri Derg, 8*, 280-287.

Azhar, A. S., AlShammasi, Z. H., & Higgi, R. E. (2016). The impact of congenital heart diseases on the quality of life of patients and their families in Saudi Arabia: Biological, psychological, and social dimensions. *Saudi Medical Journal, 37*, 392-402.

Barsella, R., Amer, K., Simonovich, S. D., & Hibino, N. (2021). Educational tool reduces parental stress at home post pediatric cardiac surgery: A pilot study. *Progress in Pediatric Cardiology, 61*, 101335.

Beck, A. T., Kovacs, M., & Weissman, A. (1975). Hopelessness and suicidal behavior: An overview. *JAMA, 234*, 1146-1149.

Beck, A. T., Steer, Robert A & Brown, Gregory K. (1996). *Beck Depression Inventory Manual*. NCS Pearson, Inc.

Beck, A. T., Weissman, A., Lester, D., & Trexler, L. (1974). The measurement of pessimism: The Hopelessness Scale. *Journal of Consulting and Clinical Psychology, 42*, 861-865.

Beck, J. G., Grant, D. M., Read, J. P., Clapp, J. D., Coffey, S. F., Miller, L. M., & Palyo, S. A. (2007). The Impact of Event Scale-Revised: Psychometric properties in a sample of motor vehicle accident survivors. *Journal of Anxiety Disorders, 22*, 187-198.

Bektas, İ., Kır, M., Yıldız, K., Genç, Z., Bektas, M., & Ünal, N. (2020). Symptom frequency in children with congenital heart disease and parental care burden in predicting the quality of life of parents in Turkey. *Journal of Pediatric Nursing, 53*, e211-e216.

Berant, E., Mikulincer, M., & Florian, V. (2001). The association of mothers' attachment style and their psychological reactions to the diagnosis of infant's congenital heart disease. *Journal of Social and Clinical Psychology, 20*, 208-232.

Berant, E., Mikulincer, M., & Florian, V. (2003). Marital satisfaction among mothers of infants with congenital heart disease: The contribution of illness severity, attachment style, and the coping process. *Anxiety, Stress and Coping, 16*, 397-415.
Bevilacqua, F., Palatta, S., Mirante, N., Cuttini, M., Seganti, G., Dotta, A., & Piersigilli, F. (2013). Birth of a child with congenital heart disease: emotional reactions of mothers and fathers according to time of diagnosis. *The Journal of Maternal-Fetal & Neonatal Medicine, 2013, 26*, 1249-1253.
Bishop, M. N., Gise, J. E., Donati, M. R., Shneider, C. E., Aylward, B. S., & Cohen, L. L. (2019). Parenting stress, sleep, and psychological adjustment in parents of infants and toddlers with congenital heart disease. *Journal of Pediatric Psychology, 44*, 980-987.
Blue, G. M., Kasparian, N. A., Sholler, G. F., Kirk, E. P., & Winlaw, D. S. (2015). Genetic counselling in parents of children with congenital heart disease significantly improves knowledge about causation and enhances psychosocial functioning. *International Journal of Cardiology, 178*, 124-130.
Bratt, E.-L., Järvholm, S., Ekman-Joelsson, B.-M., Johannsmeyer, A., Carlsson, S.-Å., Mattsson, L.-Å., & Mellander, M. (2019). Parental reactions, distress, and sense of coherence after prenatal versus postnatal diagnosis of complex congenital heart disease. *Cardiology in the Young, 29*, 1328-1334.
Breslau, N., Weitzman, M., & Messenger, K. (1981). Psychologic functioning of siblings of disabled children. *Pediatrics, 67*, 344-353.
Brock, A. D., Vermulst, A. A., Gerris, J. R. M., & Abidin, R. R. (1992). *NOSI, Nijmeegse Ouderlijke Stress Index*. Experimentele versie. Handleiding.
Brosig, C. L., Mussatto, K. A., Kuhn, E. M., & Tweddell, J. S. (2007). Neurodevelopmental outcome in preschool survivors of complex congenital heart disease: Implications for clinical practice. *Journal of Pediatric Health Care, 21*, 3-12.
Brosig, C. L., Whitstone, B. N., Frommelt, M. A., Frisbee, S. J., & Leuthner, S. R. (2007). Psychological distress in parents of children with severe congenital heart disease: The impact of prenatal versus postnatal diagnosis. *Journal of Perinatology, 27*, 687-692.
Bryant, R., Moulds, M., & Guthrie, R. M. (2000). Acute Stress Disorder Scale: A self-report measure of acute stress disorder. *Psychological Assessment, 12*, 61-68.
Bullinger, M. (1995). German translation and psychometric testing of the SF-36 Health Survey: Preliminary results from the IQOLA project. *Social Science & Medicine (1982), 41*(10), 1359-1366.
Bulut I. (1990). *Aile Değerlendirme Ölçeği El Kitabı.* Öz Güzeliş Matbaası, Ankara.
Callahan, K., Steinwurtzel, R., Brumarie, L., Schechter, S., & Parravicini, E. (2019). Early palliative care reduces stress in parents of neonates with congenital heart disease: Validation of the "Baby, Attachment, Comfort Interventions". *Journal of Perinatology, 39*, 1640-1647.
Campbell, L., Clark, M., & Kirkpatrick, S. E. (1986). Stress management training for parents and their children undergoing cardiac catheterization *American Journal of Orthopsychiatry, 56*, 234-243.
Campbell, L. A., Kirkpatrick, S. E., Berry, C. C., & Lamberti, J. J. (1995). Preparing children with congenital heart disease for cardiac surgery. *Journal of Pediatric Psychology, 20*, 313-328.
Campbell, L. A., Kirkpatrick, S. E., Berry, C. C., Penn, N. E., Waldman, J. D., & Mathewson, J. W. (1992). Psychological preparation of mothers of preschool children undergoing cardiac catheterization. *Psychology & Health, 7*, 175-185.
Cantwell-Bartl, A., & Tibballs, J. (2017). Parenting a child at home with hypoplastic left heart syndrome: Experiences of commitment, of stress, and of love. *Cardiology in the Young, 27*, 1341-1348.
Carey, L. K., Nicholson, B. C., & Fox, R. A. (2002). Maternal factors related to parenting young children with congenital heart disease. *Journal of Pediatric Nursing, 17*(3), 174-183.
Caris, E. C., Dempster, N., Wernovsky, G., Butz, C., Neely, T., Allen, R., Stewart, J., Miller-Tate, H., Fonseca, R., Texter, K., Nicholson, L., & Cua, C. L. (2016). Anxiety scores in caregivers of children with hypoplastic left heart syndrome. *Congenital Heart Disease, 11*, 727-732.
Caris, E. C., Dempster, N., Wernovsky, G., Miao, Y., Moore-Clingenpeel, M., Neely, T., Fonseca, R., Miller-Tate, H., Allen, R., Fichtner, S., Stewart, J., & Cua, C. L. (2018). Perception scores of siblings and parents of children with hypoplastic left heart syndrome. *Congenital Heart Disease, 13*, 528-532.
Carver, C. (1997). You want to measure coping but your protocol's too long: Consider the brief cope. *International Journal of Behavioral Medicine, 4*, 92-100.

Carver, C. S., Scheier, M. F., & Kumari Weintraub, J. (1989). Assessing coping strategies: A theoretically based approach. *Journal of Personality and Social Psychology, 56*, 267-283.
Chaisom, P., Yenbut, J., Chontawan, R., Soivong, P., & Patumanond, J. (2010). Predicting factors of dependent care behaviors among mothers of toddlers with congenital heart disease. *Chiang Mai University Journal of Natural Sciences, 9*, 193-200.
Chan, K. S., Li, H. C. W., Chan, S. W. c., & Lopez, V. (2012). Herth Hope Index: Psychometric testing of the Chinese version. *Journal of Advanced Nursing, 68*, 2079-2085.
Chang, L. Y., Chiu, S. N., Wang, C. C., Weng, W. C., & Chang, H. Y. (2020). Parenting stress mediates the association between cyanotic congenital heart disease and internalising problems in children and adolescents. *European Journal of Cardiovascular Nursing, 19*, 301-309.
Chen, C., Zhang, H. J., Jiang, H., Li, W. J., & Lü, L. (2010). Assessing the general mental health of college students: psychometric properties of GHQ-28. *Journal of Shandong University (Health Science), 48*(3), 159-162.
Cheng, M. (2011). *The Ottawa Mood Scales*. Ottawa: Michael Cheng.
Chesney, M. A., Chambers, D. B., Taylor, J. M., Johnson, L. M., & Folkman, S. (2003). Coping effectiveness training for men living with HIV: Results from a randomized clinical trial testing a group-based intervention. *Psychosomatic Medicine, 65*, 1038-1046.
Chien, C. H., Lee, T. Y., & Lin, M. T. (2021). Factors affecting motor development of toddlers who received cardiac corrective procedures during infancy. *Early Human Development, 158*, 105392.
Choi, Y., & Lee, S. (2021). Coping self-efficacy and parenting stress in mothers of children with congenital heart disease. *Heart and Lung, 50*, 352-356.
Cohen, S., & Williamson, G. (1988). Perceived stress in a probability sample of the US. In: Spacapam S, Oskamp S. (Eds), *The Social Psychology of Health: Claremont Symposium on Applied Social Psychology.* SAGE Publications.
Cohn, J. K. (1996). An empirical study of parents' reaction to the diagnosis of congenital heart disease in infants. *Social Work in Health Care, 23*, 67-79.
Coşkuntürk, A. E., & Gözen, D. (2018). The effect of interactive therapeutic play education program on anxiety levels of children undergoing cardiac surgery and their mothers. *Journal of Perianesthesia Nursing, 33*, 781-789.
Cox, J. L., Holden, J. M., & Sagovsky, R. (1987). Detection of postnatal depression. Development of the 10-item Edinburgh Postnatal Depression Scale. *The British Journal of Psychiatry, 150*, 782-786.
Davis, C. C., Brown, R. T., Bakeman, R., & Campbell, R. (1998). Psychological adaptation and adjustment of mothers of children with congenital heart disease: Stress, coping, and family functioning. *Journal of Pediatric Psychology, 23*, 219-228.
De Stasio, S., Boldrini, F., Ragni, B., Bevilacqua, F., Bucci, S., Giampaolo, R., Messina, V., & Gentile, S. (2019). Sleep quality, emotion regulation and parenting stress in children with congenital heart disease. *Mediterranean Journal of Clinical Psychology, 7*(3), 1-20.
de Vries, J., & van Heck, G. L. (2013). Development of a short version of the Dutch version of the Spielberger STAI trait anxiety scale in women suspected of breast cancer and breast cancer survivors. *Journal of Clinical Psychology in Medical Settings, 20*, 215-226.
Denniss, D. L., Sholler, G. F., Costa, D. S. J., Winlaw, D. S., & Kasparian, N. A. (2019). Need for routine screening of health-related quality of life in families of young children with complex congenital heart disease. *The Journal of Pediatrics, 205*, 21-28.e2.
Derogatis, L. R. (2001). *BSI 18, Brief Symptom Inventory 18: Administration, scoring and procedures manual*. NCS Pearson, Incorporated.
Derogatis, L. R., & Cleary, P. A. (1977). Confirmation of the dimensional structure of the scl-90: A study in construct validation. *Journal of Clinical Psychology, 33*, 981-989.
Derogatis, L. R., & Melisaratos, N. (1983). The Brief Symptom Inventory: An introductory report. *Psychological Medicine, 13*, 595-605.
Derogatis, L. R., Rickels, K., & Rock, A. F. (1976). The SCL-90 and the MMPI: A step in the validation of a new self-report scale. *British Journal of Psychiatry, 128*, 280-289.
Derogatis, L. R., & Savitz, K. L. (1999). The SCL-90-R, Brief Symptom Inventory, and Matching Clinical Rating Scales.

DeVet, K. A., & Ireys, H. T. (1998). Psychometric properties of the Maternal Worry Scale for children with chronic illness. *Journal of Pediatric Psychology, 23*, 257-266.
Diffin, J., Spence, K., Naranian, T., Badawi, N., & Johnston, L. (2016). Stress and distress in parents of neonates admitted to the neonatal intensive care unit for cardiac surgery. *Early Human Development, 103*, 101-107.
Doherty, N., McCusker, C. G., Molloy, B., Mulholland, C., Rooney, N., Craig, B., Sands, A., Stewart, M., & Casey, F. (2009). Predictors of psychological functioning in mothers and fathers of infants born with severe congenital heart disease. *Journal of Reproductive and Infant Psychology, 27*, 390-400.
Dulfer, K., Duppen, N., Van Dijk, A. P., Kuipers, I. M., Van Domburg, R. T., Verhulst, F. C., van der Ende, J., Helbing, W.A., & Utens, E. M. (2015). Parental mental health moderates the efficacy of exercise training on health-related quality of life in adolescents with congenital heart disease. *Pediatric Cardiology, 36*, 33-40.
Eagleson, K. J., Justo, R. N., Ware, R. S., Johnson, S. G., & Boyle, F. M. (2013). Health-related quality of life and congenital heart disease in Australia. *Journal of Paediatrics and Child Health, 49*, 856-864.
Edraki, M., Kamali, M., Beheshtipour, N., Amoozgar, H., Zare, N., & Montaseri, S. (2014). The effect of educational program on the quality of life and self-efficacy of the mothers of the infants with congenital heart disease: A randomized controlled trial. *International Journal of Community-Based Nursing Midwifery, 2*, 51-59.
El Missiry, A., Sorour, A., Sadek, A., Fahy, T., Abdel Mawgoud, M., & Asaad, T. (2003). Homicide and psychiatric illness: an Egyptian study [MD thesis]. Cairo: Faculty of Medicine, Ain Shams University.
Elo, A.-L., Leppänen, A., & Jahkola, A. (2003). Validity of a single-item measure of stress symptoms. *Scandinavian Journal of Work, Environment & Health, 29*, 444-451.
Endler, N. S., & Parker, J. D. A. (1994). Assessment of multidimensional coping: Task, emotion, and avoidance strategies. *Psychological Assessment, 6*, 50-60.
Epstein, N. B., Baldwin, L.M, & Bishop, D.S. (1983). The McMaster Family Assessment Device. *Journal of Marital and Family Therapy, 9*, 171-180.
Eser, E., Fidaner, H., Fidaner, C., Eser, S. Y., Elbi, H., & Göker, E. (1999). WHOQOL-100 ve WHOQOL-BREF'in psikometrik özellikleri. *Psikiyatri Psikoloji Psikofarmakoloji (3P) Dergisi, 7(Suppl 2)*, 23-40.
Ezzat, S., Saeedi, O., Saleh, D. A., Hamzeh, H., Hamid, M. A., Crowell, N., Boostrom, C., Loffredo, C. & Jillson, I. A. (2016). Parental perceptions of congenital cardiovascular malformations in their children. *Cardiology in the Young, 26*, 1158-1167.
Fischer, A. L., Butz, C., Nicholson, L., Blankenship, A., Dyke, P., & Cua, C. L. (2012). Caregiver anxiety upon discharge for neonates with congenital heart disease. *Congenital Heart Disease, 7*, 41-45.
Foa, E. B., Cashman, L., Jaycox, L., & Perry, K. (1997). The validation of a self-report measure of posttraumatic stress disorder: the Posttraumatic Diagnostic Scale. *Psychological Assessment, 9*, 445-451.
Folkman, S., & Lazarus, R. S. (1985). If it changes it must be a process: study of emotion and coping during three stages of a college examination. *Journal of Personality and Social Psychology, 48*, 150-170.
Fontanesi, F., Gobetti, C., Zimmermann-Tansella, C., & Tansella, M. (1985). Validation of the Italian version of the GHQ in a general practice setting. *Psychological Medicine, 15*, 411-415.
Fowers, B. J., & Olson, D. H. (1989). ENRICH marital inventory: a discriminant validity and cross-validation assessment. *Journal of Marital Family Therapy, 15*, 65-79.
Franck, L. S., McQuillan, A., Wray, J., Grocott, M. P., & Goldman, A. (2010, Oct). Parent stress levels during children's hospital recovery after congenital heart surgery. *Pediatric Cardiology, 31*, 961-968.
Franich-Ray, C., Bright, M. A., Anderson, V., Northam, E., Cochrane, A., Menahem, S., & Jordan, B. (2013). Trauma reactions in mothers and fathers after their infant's cardiac surgery. *Journal of Pediatric Psychology, 38*, 494-505.
Frydenberg, E., & Lewis, R. (2014). *Coping scale for adults – second edition (CSA-2)*. Melbourne, Australia: ACER Press.
Fugl-Meyer, A. R., Melin, R., & Fugl-Meyer, K. S. (2002). Life satisfaction in 18-to 64-year-old Swedes: in relation to gender, age, partner and immigrant status. *Journal of Rehabilitation Medicine, 34*(5), 239-246.
Furman, W., & Buhrmester, D. (1985). Children's perceptions of the qualities of sibling relationships. *Child Development*, 448-461.

Garcia, R. U., Aggarwal, S., & Natarajan, G. (2016). Parental perception of functional status and impact on the family of children with congenital heart surgery. *Early Human Development, 96*, 45-51.

Gaskin, K. L., Barron, D., & Wray, J. (2021). Parents' experiences of transition from hospital to home after their infant's first-stage cardiac surgery: Psychological, physical, physiological, and financial survival. *The Journal of Cardiovascular Nursing, 36*, 283-292.

Ghareeb, A. (2000). *Inventory for measuring depression BDI-II (2-D), instructions, validity, reliability, tables of standard and scoring index*. Cairo: Anglo Egyptian Library.

Goldbeck, L., M. Storck. (2002). Das Ulmer Lebensqualitäts-Inventar für Eltern chronisch kranker kinder (ULQIE). [ULQIE: a quality-of-life inventory for parents of chronically ill children]. Zeitschrift für Klinische Psychologie und Psychotherapie*, 31*, 31-39.

Goldbeck, L., & Melches, J. (2006). The impact of the severity of disease and social disadvantage on quality of life in families with congenital cardiac disease. *Cardiology in the Young, 16*, 67-75.

Goldberg, D. P., & Blackwell, B. (1970). Psychiatric illness in General Practice: A detailed study using a new method of case identification. *British Medical Journal, 2*(5707), 439-443.

Goldberg, S., Simmons, R. J., Newman, J., Campbell, K., & Fowler, R. S. (1991). Congenital heart disease, parental stress, and infant-mother relationships. *Journal of Pediatrics, 119*, 661-666.

Golfenshtein, N., Hanlon, A. L., Deatrick, J. A., & Medoff-Cooper, B. (2017). Parenting stress in parents of infants with congenital heart disease and parents of healthy infants: the first year of life. *Comprehensive Child and Adolescent Nursing*, *40*, 294-314.

Griesel, D., Wessa, M., & Flor, H. (2006). Psychometric qualities of the German version of the Posttraumatic Diagnostic Scale (PTDS). *Psychological Assessment, 18*, 262-268.

Guan, G., Liu, H., Wang, Y., Han, B., & Jin, Y. (2013). Behavioural and emotional outcomes in school-aged children after surgery or transcatheter closure treatment for ventricular septal defect. *Cardiology in the Young, 24*, 910-917.

Hancock, H. S., Pituch, K., Uzark, K., Bhat, P., Fifer, C., Silveira, M., Yu, S., Welch, S., Donohue, J., Lowery, R., & Aiyagari, R. (2018). A randomised trial of early palliative care for maternal stress in infants prenatally diagnosed with single-ventricle heart disease. *Cardiology in the Young, 28*, 561-570.

Harder, D. H., & Zalrna, A. (1990). Two promising shame and guilt scales: A construct validity comparison. *Journal of Personality Assessment, 55*(3-4), 729-745.

Haverman, L., van Oers, H. A., Limperg, P. F., Houtzager, B. A., Huisman, J., Darlington, A.-S., Maurice-Stam, J., & Grootenhuis, M. A. (2013). Development and validation of the distress thermometer for parents of a chronically ill child. *The Journal of Pediatrics, 163*, 1140-1146.

Hearps, S. J., McCarthy, M. C., Muscara, F., Hearps, S. J. C., Burke, K., Jones, B., & Anderson, V. A. (2014). Psychosocial risk in families of infants undergoing surgery for a serious congenital heart disease. *Cardiology in the Young, 24*, 632-639.

Helfricht, S., Latal, B., Fischer, J. E., Tomaske, M., & Landolt, M. A. (2008). Surgery-related posttraumatic stress disorder in parents of children undergoing cardiopulmonary bypass surgery: A prospective cohort study. *Pediatric Critical Care Medicine, 9*, 217-223.

Hisli, N. (1989). Beck depresyon envanterinin universite ogrencileri icin gecerliligi, guvenilirligi. (A reliability and validity study of Beck Depression Inventory in a university student sample). *Journal of Psychology, 7*, 3-13.

Hoehn, K. S., Wernovsky, G., Rychik, J., Tian, Z.-y., Donaghue, D., Alderfer, M. A., Gaynor, J.W., Kazak, A.E., Spray, T.L., & Nelson, R. M. (2004). Parental decision-making in congenital heart disease. *Cardiology in the Young, 14*, 309-314.

Holmes, T. H., & Rahe, R. H. (1967). The social readjustment rating scale. *Journal of Psychosomatic Research, 11*, 213-218.

Hornblow, A. R., & Kidson, M. A. (1976). The visual analogue scale for anxiety: A validation study. *Australian and New Zealand Journal of Psychiatry, 10*(4), 339-341.

Humphreys, D., Ibáñez, C., Fullerton, C., Acuña, J., Florenzano, R., & Marchandon, A. (1991). *Validación preliminar en Chile de una versión abreviada del Cuestionario General de Salud de Goldberg (GHQ-12)*. XLVI Jornadas anuales de la Sociedad de Neurología, Psiquiatría y Neurocirugía.

Hunt, A. V., Hilton, D. C. K., Verrall, C. E., Barlow-Stewart, K. K., Fleming, J., Winlaw, D. S., & Blue, G. M. (2020). Why and how did this happen? Development and evaluation of an information resource for parents of children with CHD. *Cardiology in the Young, 30*, 346-352.

Hussein, K. A., & Authman, N. R. (2013). Quality of life for caregivers of children with congenital heart disease in surgical specialty hospital–Cardiac Center Kurdistan Region/Iraq. *Journal of Kufa for Nursing Science Vol, 3*(2), 148-161.

İnci, F., & Erdem, M. (2008). Bakım Verme Yükü Ölçeği'ninTürkçe'ye uyarlanmasıgeçerlilik ve güvenilirliği. *Journal of Anatolia Nursing and Health Sciences*, 11(4), 85-95.

Jackson, A. C., Frydenberg, E., Koey, X. M., Fernandez, A., Higgins, R. O., Stanley, T., Liang, R.P., Le Grande, M.R. & Murphy, B. M. (2020). Enhancing parental coping with a child's heart condition: A co-production pilot study. *Comprehensive Child and Adolescent Nursing, 43*, 314-333.

Jackson, J. L., Gerardo, G. M., Daniels, C. J., & Vannatta, K. (2017). Perceptions of disease-related stress: A key to better understanding patient-reported outcomes among survivors of congenital heart disease. *Journal of Cardiovascular Nursing, 32*, 587-593.

Jantien Vrijmoet-Wiersma, C. M. J., Hoekstra-Weebers, J. E. H. M., Margreet de Peinder, W. M. G., Koopman, H. M., Tissing, W. J. E., Treffers, P. D. A., Bierings, M.B., Jansen, N.C.A, Grootenhuis, M.A, & Egeler, R. M. (2010). Psychometric qualities of the Dutch version of the Pediatric Inventory for Parents (PIP): A multi-center study. *Psycho-Oncology, 19*, 368-375.

Jantien Vrijmoet-Wiersma, C. M., Ottenkamp, J., van Roozendaal, M., Grootenhuis, M. A., & Koopman, H. M. (2009). A multicentric study of disease-related stress, and perceived vulnerability, in parents of children with congenital cardiac disease. *Cardiology in the Young, 19*, 608-614.

Janus, M., & Goldberg, S. (1997). Treatment characteristics of congenital heart disease and behaviour problems of patients and healthy siblings. *Journal of Paediatrics and Child Health, 33*, 219-225.

Jordan, B., Franich-Ray, C., Albert, N., Anderson, V., Northam, E., Cochrane, A., & Menahem, S. (2014). Early mother-infant relationships after cardiac surgery in infancy. *Archives of Disease in Childhood, 99*, 641.

Kaugars, A., Shields, C., & Brosig, C. (2018). Stress and quality of life among parents of children with congenital heart disease referred for psychological services. *Congenital Heart Disease, 13*, 72-78.

Kiliçarskan-Törüner E, K. F., Keçialan R. (2012). Effect of planned information provision on anxiety of parents of children who underwent cardiac surgery. *Hemşirelikte Araştırma Geliştirme Dergisi, 14*(1), 11-20.

Kim, D. H. (1997). *A comparative study of parenting stress between mothers of normal neonate and low birth weight neonate*. Unpublished Master's Thesis, Yonsei University, Seoul.

Kim, J. T., & Shin, D. K. (1978). A study based on the standardization of the STAI for Korea. *The New Medical Journal, 21(11)*, 69-75.

Kodraliu G, M. P., Groth N, Carmosino G, Perilli A, Gianicolo, E.A.L, Rossi C and Apolone G. (2001). Subjective health status assessment: Evaluation of the Italian version of the SF-12 Health Survey. Results from the MiOS Project. *Journal of Epidemiology and Biostatistics, 6*(3), 305-316.

Koeter, M. W. J., & Ormel, J. (1991). *General Health Questionnaire. Dutch manual*. Lisse: Swets & Zeitlinger BV.

Kroenke, K., & Spitzer, R.L. (2002). The PHQ-9: a new depression diagnostic and severity measure. *Psychiatric Annals*, *32*, 509-515.

Kroenke, K., Spitzer, R.L., & Williams, J.B.W. (2001). The PHQ-9: validity of a brief depression severity measure. *Journal of General Internal Medicine, 16*, 606-613.

Kumar, A., Das, S., Chauhan, S., Kiran, U., & Satapathy, S. (2019). Perioperative anxiety and stress in children undergoing congenital cardiac surgery and their parents: Effect of brief intervention-a randomized control trial. *Journal of Cardiothoracic Vascular Anesthesia, 33*, 1244-1250.

Kuyken, W., Orley, J., Power, M., Herrman, H., Schofield, H., Murphy, B., Metelko, Z.,Szabo, S., Pibernik-Okanovic, M., Quemada, N., Caria, A., Rajkumar, S., Kumar, S, Saxena, S., Bar-On, D., Amir, M., Tazaki, M., Noji, A., Vanheck, G..., & Burkovsky, G. (1995). The World Health Organization quality of life assessment (WHOQOL): position paper from the World Health Organization. *Social Science &Medicine, 41*, 1403-1409.

Ladak, L. A., Hasan, B. S., Gullick, J., Awais, K., Abdullah, A., & Gallagher, R. (2019). Health-related quality of life in surgical children and adolescents with congenital heart disease compared with their age-matched healthy sibling: A cross-sectional study from a lower middle-income country, Pakistan. *Archives of Disease in Childhood, 104*, 419-425.

Landolt, M. A., Buechel, E. V., & Latal, B. (2011). Predictors of parental quality of life after child open heart surgery: a 6-month prospective study. *The Journal of Pediatrics, 158*, 93-99.

Lawoko, S., & Soares, J. J. (2006). Psychosocial morbidity among parents of children with congenital heart disease: a prospective longitudinal study. *Heart & Lung, 35*(5), 301-314.

Lazarus, R. S., & Folkman, S. (1989). *Manual Hassles and uplifts scales: Sampler set: Manual and test booklet* (Research ed. ed.). Mind Garden.

Lee, J. S., Cinanni, N., Di Cristofaro, N., Lee, S., Dillenburg, R., Adamo, K. B., Mondal, T., Barrowman, N., Shanmugam, G., Timmons., & Longmuir, P. W. (2020). Parents of very young children with congenital heart defects report good quality of life for their children and families regardless of defect severity. *Pediatric Cardiology, 41*, 46-53.

Lee, R. L. T., & Mok, E. S. B. (2011). Evaluation of the psychometric properties of a modified Chinese version of the Caregiver Task Inventory–refinement and psychometric testing of the Chinese Caregiver Task Inventory: A confirmatory factor analysis. *Journal of Clinical Nursing, 20*(23-24), 3452-3462.

Lee, S., Yoo, J.-S., & Yoo, I.-Y. (2007). Parenting stress in mothers of children with congenital heart disease. *Asian Nursing Research, 1*, 116-124.

Levert, E. M., Helbing, W. A., Dulfer, K., van Domburg, R. T., & Utens, E. M. (2017). Psychosocial needs of children undergoing an invasive procedure for a CHD and their parents. *Cardiology in the Young, 27*, 243-254.

Li, Y., Solomon, P., Zhang, A., Franklin, C., Ji, Q., & Chen, Y. (2018). Efficacy of solution-focused brief therapy for distress among parents of children with congenital heart disease in China. *Health & Social Work, 43*, 30-40.

Lisanti, A. J., Allen, L. R., Kelly, L., & Medoff-Cooper, B. (2017). Maternal stress and anxiety in the Pediatric Cardiac Intensive Care Unit. *American Journal of Critical Care, 26*, 118-125.

Lisanti, A. J., Demianczyk, A. C., Costarino, A., Vogiatzi, M. G., Hoffman, R., Quinn, R., Chittams, J.L, & Medoff-Cooper, B. (2021a). Skin-to-skin care is associated with reduced stress, anxiety, and salivary cortisol and improved attachment for mothers of infants with critical congenital heart disease. *Journal of Obstetric, Gynecologic, and Neonatal Nursing, 50*, 40-54.

Lisanti, A. J., Kumar, A., Quinn, R., Chittams, J. L., Medoff-Cooper, B., & Demianczyk, A. C. (2021b). Role alteration predicts anxiety and depressive symptoms in parents of infants with congenital heart disease: A pilot study. *Cardiology in the Young, 31*, 1842-1849.

Lobato, D. J., & Kao, B. T. (2002). Integrated sibling-parent group intervention to improve sibling knowledge and adjustment to chronic illness and disability. *Journal of Pediatric Psychology, 27*, 711-716.

Lopez, R., Frangini, P., Ramirez, M., Valenzuela, P. M., Terrazas, C., Perez, C. A., Borchert, E., & Trachsel, M. (2016). Well-being and agency in parents of children with congenital heart disease: A survey in Chile. *World Journal of Pediatric Congenital Heart Surgery, 7*, 139-145.

Lovibond, S.H., & Lovibond P.F. (1995). Manual for the Depression Anxiety Stress Scales (2nd ed.) Psychology Foundation of Australia.

Lu, L., Wang, L., Yang, X., & Feng, Q. (2009). Zarit Caregiver Burden Interview: Development, reliability and validity of the Chinese version. *Psychiatry and Clinical Neurosciences, 63*, 730-734.

Majnemer, A., Limperopoulos, C., Shevell, M., Rohlicek, C., Rosenblatt, B., & Tchervenkov, C. (2006). Health and well-being of children with congenital cardiac malformations, and their families, following open-heart surgery. *Cardiology in the Young, 16*, 157-164.

Maslach, C., Jackson, S. E., & Leiter, M. P. (1996). *Maslach Burnout Inventory manual* (3rd ed.). Consulting Psychologists Press.

McCubbin, H. I., & Patterson, J. (1983). Family Member Wellbeing-Index (FMWB). In H.I. McCubbin, A.I Thompson, & M.A. McCubbin (Eds.), *Family assessment: Resiliency, coping and adaptation - Inventories for research and practice* (pp. 753-782). University of Wisconsin Press.

McCubbin, H.I., Patterson, J., & Wilson, L. (1983). Family Inventory of Life Events and Changes (FILE). In H.I. McCubbin, A.I Thompson, & M.A. McCubbin (Eds.), *Family assessment: Resiliency, coping and adaptation - Inventories for research and practice*. (pp. 103-178). University of Wisconsin Press].

McCubbin, H. I., McCubbin, M. A., Nevin, R., & Cauble, E. (1981). Coping health inventory for parents (CHIP). In H.I. McCubbin, A.I Thompson, & M.A. McCubbin (Eds.), *Family assessment: Resiliency, coping, and adaptation-Inventories for research and practice,* 407-453.

McCubbin, H. I., McCubbin, M. A., Patterson, J. M., Cauble, A. E., Wilson, L. R., & Warwick, W. (1983). CHIP. Coping Health Inventory for Parents: An assessment of parental coping patterns in the care of the chronically ill child. *Journal of Marriage and the Family, 45*, 359-370.

McCubbin, M. A., McCubbin, H. I., & Thompson, A. I. (1986). Family Hardiness Index (FHI). In A. I. T. H.I. McCubbin, & M.A. McCubbin (Ed.), *Family assessment: Resiliency, coping and adaptation - Inventories for research and practice* (pp. pp. 239-305). University of Wisconsin Press.

McCusker, C. G., Doherty, N. N., Molloy, B., Rooney, N., Mulholland, C., Sands, A., Craig, B., Stewart, M., & Casey, F. (2010). A controlled trial of early interventions to promote maternal adjustment and development in infants born with severe congenital heart disease. *Child: Care, Health and development, 36*, 110-117.

McCusker, C. G., Doherty, N. N., Molloy, B., Rooney, N., Mulholland, C., Sands, A., Craig, B., Stewart, M., & Casey, F. (2012). A randomized controlled trial of interventions to promote adjustment in children with congenital heart disease entering school and their families. *Journal of Pediatric Psychology, 37*, 1089-1103.

McKechnie, A. C., Pridham, K., & Tluczek, A. (2016). Walking the "Emotional Tightrope" from Pregnancy to Parenthood: Understanding Parental Motivation to Manage Health Care and Distress after a Fetal Diagnosis of Complex Congenital Heart Disease. *Journal of Family Nursing, 22*, 74-107.

Medoff-Cooper, B., Marino, B. S., Fleck, D. A., Lisanti, A. J., Golfenshtein, N., Ravishankar, C., Costello, J.M., Huang, L., Hanlon, A.L., , & Curley, M. A. Q. (2020). Telehealth home monitoring and postcardiac surgery for congenital heart disease. *Pediatrics, 146*, e20200531.

Menahem, S., Poulakis, Z., & Prior, M. (2008). Children subjected to cardiac surgery for congenital heart disease. Part 2 - Parental emotional experiences. *Interactive Cardiovascular and Thoracic Surgery, 7*, 605-608.

Miles, M. S., & Brunssen, S. H. (2003). Psychometric properties of the parental stressor scale: Infant hospitalization. *Advanced Neonatal Care, 3*, 189-196.

Miles, M. S., Funk, S. G., & Carlson, J. (1993). Parental Stressor Scale: Neonatal Intensive Care Unit. *Nursing Research, 42*, 148-152.

Miller, I. W., Epstein, N. B., Bishop, D. S., & Keitner, G. I. (1985). The McMaster Family Assessment Device: Reliability and validity. *Journal of Marital and Family Therapy, 11*, 345-356.

Miller, V. A., Newcombe, J., Radovich, P., Johnston, F., Medina, E., Jr., & Nelson, A. (2021). The healing hearts at home© mobile application usability and influence on parental perceived stress: A pilot study. *International Journal of E-Health and Medical Communications, 12*, 90-105.

Montazeri, A., Vahdaninia, M., Mousavi, S. J., & Omidvari, S. (2009). The Iranian version of 12-item Short Form Health Survey (SF-12): Factor structure, internal consistency and construct validity. *BMC Public Health, 9*, 341-341.

Moon, Y., Jung, J. W., & Lee, S. (2021). Sibling relationships of adolescents with congenital heart disease. *International Journal of Environmental Research and Public Health, 18*(5), 1-9.

Moons, P., Deyk, K. V., Bleser, L. D., Marquet, K., Raes, E., Geest, S. D., & Budts, W. (2006). Quality of life and health status in adults with congenital heart disease: A direct comparison with healthy counterparts. *European Journal of Preventive Cardiology, 13*, 407-413.

Moos, R. H. (1990). Conceptual and empirical approaches to developing family-based assessment procedures: Resolving the case of the Family Environment Scale. *Family Process, 29*, 199-208.

Mörelius, E.-L., Ulla, U., & Nelson, N. (2002). Parental stress in relation to the severity of congenital heart disease in the offspring. *Pediatric Nursing, 28*, 28-34.

Murphy, B. M., Higgins, R. O., Shand, L., Page, K., Holloway, E., Le Grande, M. R., & Jackson, A. C. (2017). Improving health professionals' self-efficacy to support cardiac patients' emotional recovery: The 'Cardiac Blues Project'. *European Journal of Cardiovascular Nursing, 16*, 143-149.

Mussatto, K. A., Van Rompay, M. I., Trachtenberg, F. L., Pemberton, V., Young-Borkowski, L., Uzark, K., Dunbar-Masterson, C., Infinger, P., Walter., P.,, & Sawin, K. (2021). Family function, quality of life, and well-being in parents of infants with hypoplastic left heart syndrome. *Journal of Family Nursing, 27*, 222-234.

Östberg, M., Hagekull, B., & Wettergren, S. (1997). A measure of parental stress in mothers with small children: Dimensionality, stability and validity. *Scandinavian Journal of Psychology, 38*, 199-208.

Özer, N., Yurttaş, A., & Akyıl, R. Ç. (2012). Psychometric evaluation of the Turkish version of the Zarit Burden Interview in family caregivers of inpatients in medical and surgical clinics. *Journal of Transcultural Nursing, 23*, 65-71.

Page, M. J., McKenzie, J. E., Bossuyt, P. M., Boutron, I., Hoffmann, T. C., Mulrow, C. D., Shamseer, L., Tetzlaff, J.M., Akl, E.A., Brennan, S.E., Choi, R., Glanville, J., Grimshaw, J.M., Hrobjartsson, A., Lalu, M.M., Li, T., Loder, E.W., Mayo-Wilson, E., McDonald, S... & Moher, D. (2021). The PRISMA 2020 statement: An updated guideline for reporting systematic reviews. *International Journal of Surgery*, *88*, 105906.

Pai, A. L. H., Patiño-Fernández, A. M., McSherry, M., Beele, D., Alderfer, M. A., Reilly, A. T., Hwang, W., & Kazak, A. E. (2008). The Psychosocial Assessment Tool (PAT2.0): Psychometric properties of a screener for psychosocial distress in families of children newly diagnosed with cancer. *Journal of Pediatric Psychology, 33*, 50-62.

Patrick, D. L., Kinne, S., Engelberg, R. A., & Pearlman, R. A. (2000). Functional status and perceived quality of life in adults with and without chronic conditions. *Journal of Clinical Epidemiology, 53*, 779-785.

Pearson, V., & Chan, T. W. L. (1993). The relationship between parenting stress and social support in mothers of children with learning disabilities: A Chinese experience. *Social Science & Medicine, 37*, 267-274.

Pinar, R. (2005). Reliability and construct validity of the SF-36 in Turkish cancer patients. *Quality of Life Research, 14*, 259-264.

Pinto, N. M., Weng, C., Sheng, X., Simon, K., Byrne, J. B., Miller, T., & Puchalski, M. D. (2016). Modifiers of stress related to timing of diagnosis in parents of children with complex congenital heart disease. *The Journal of Maternal-Fetal & Neonatal Medicine, 29*, 3340-3346.

Poh, P. F., Lee, J. H., Loh, Y. J., Tan, T. H., & Cheng, K. K. F. (2020). Readiness for hospital discharge, stress, and coping in mothers of children undergoing cardiac surgeries: A single-center prospective study. *Pediatric Critical Care Medicine*, E301-E310.

Power, M. J., Kuyken, W., Orley, J., Herman, H., Schofield, H., Murphy, B., Metelko, Z., Szabo, S., Pibernik-Okanovic, M., Quemada, N., Caria, A., Rajkumar, S., Kumar, S., Saxena, S., Chandiramani, K., Amir, M., Bar-on, D., Noji, A., van Heck, G.L., & de Vries, J. (1998). The World Health Organization quality of life assessment (WHOQOL): Development and general psychometric properties. *Social Science & Medicine, 46*(12), 1569-1585.

Prinsen, C. A. C., Vohra, S., Rose, M. R., Boers, M., Tugwell, P., Clarke, M., Williamson, P. R., & Terwee, C. B. (2016). How to select outcome measurement instruments for outcomes included in a “Core Outcome Set” – a practical guideline. *Trials, 17*(1), 449.

Radloff, L. S. (1977). The CES-D Scale: A self-report depression scale for research in the general population. *Applied Psychological Measurement, 1*, 385-401.

Rahe, R. H., Mahan, J. L., & Arthur, R. J. (1970). Prediction of near-future health change from subjects' preceding life changes. *Journal of Psychosomatic Research, 14*, 401-406.

Rahimianfar, A. A., Forouzannia, S. K., Sarebanhassanabadi, M., Dehghani, H., Namayandeh, S. M., Khavary, Z., Rahimianfar, F., & Aghbageri, H. (2015). Anxiety determinants in mothers of children with congenital heart diseases undergoing cardiac surgery. *Advanced Biomedical Research, 4*(1), 255.

Ravens-Sieberer, U., Morfeld, M., Stein, R. E.K., Jessop, D. J., Bullinger, M., & Thyen, U. (2001). Der Familien-Belastungs-Frage-bogen (FaBel-Fragebogen). *PPmP Psychotherapie Psychosomatik Medizinische Psychologie, 51*, 384-393.

Re, J. M., Dean, S., Mullaert, J., Guedeney, A., & Menahem, S. (2018). Maternal distress and infant social withdrawal (ADBB) following infant cardiac surgery for congenital heart disease. *World Journal for Pediatric and Congenital Heart Surgery, 9*, 624-637.

Reid, T., Bramwell, R., Booth, N., & Weindling, A. M. (2007). A new stressor scale for parents experiencing neonatal intensive care: the NUPS (Neonatal Unit Parental Stress) scale. *Journal of Reproductive and Infant Psychology, 25*, 66-82.

Riikonen, A., Aho, A. L., & Rantanen, A. (2019). The relationship satisfaction of parents to children with congenital heart disease. *Interpersona, 13*, 57-71.

Roberts, S. D., Kazazian, V., Ford, M. K., Marini, D., Miller, S. P., Chau, V., Seed, M., Linh, G.L., Williams, T.S., & Sananes, R. (2021). The association between parent stress, coping and mental health, and neurodevelopmental outcomes of infants with congenital heart disease. *Clinical Neuropsychologist, 35*, 948-972.

Rona, R., Smeeton, N., Beech, R., Barnett, A., & Sharland, G. (1998). Anxiety and depression in mothers related to severe malformation of the heart of the child and foetus. *Acta Pædiatrica, 87*, 201-205.

Roosa, M., & Beals, J. (1990). Measurement issues in family assessment: the case of the Family Environment Scale. *Family Process, 29*(2), 191-211.

Rychik, J., Donaghue, D. D., Levy, S., Fajardo, C., Combs, J., Zhang, X., Szwast, A., & Diamond, G. S. (2013). Maternal psychological stress after prenatal diagnosis of congenital heart disease. *The Journal of Pediatrics, 162*, 302-307.e1.

Sahler, O. J. Z., & Carpenter, P. J. (1989). Evaluation of a camp program for siblings of children with cancer. *American Journal of Diseases of Children, 143*, 690-696.

Sarajuuri, A., Lonnqvist, T., Schmitt, F., Almqvist, F., & Jokinen, E. (2012). Patients with univentricular heart in early childhood: parenting stress and child behaviour. *Acta Paediatrica, 101*, 252-257.

Sarason, I. G., Johnson, J. H., & Siegel, J. M. (1978). Assessing the impact of life changes: Development of the Life Experiences Survey. *Journal of Consulting and Clinical Psychology, 46*, 932-946.

Scheier, M. F., & Carver, C. S. (1985). Optimism, coping, and health: assessment and implications of generalized outcome expectancies. *Health Psychol, 4*(3), 219-247. https://doi.org/10.1037/0278-6133.4.3.219

Schreurs, P., Van de Willege, G, Brosschot JF, Tellegen B, Graus GMH. (1993). *Herziene handleiding Utrechtse Coping Lijst (UCL)*. Swets & Zeitlinger B.V.

Shenaar-Golan, V. W., Ofra. (2015). Mother-daughter relationship and daughter's body image. *Health, 7*, 547-559.

Sica, C. G. M. (2007). The Italian Versions of the Beck Anxiety Inventory and the Beck Depression Inventory-II: Psychometric Properties and Discriminant Power. In M. A. Lange (Ed.), *Leading-Edge Psychological Tests and Testing Research*. Nova Science Publishers, Inc.

Simeone, S., Platone, N., Perrone, M., Marras, V., Pucciarelli, G., Benedetti, M., Dell'Angelo, G., Rea, T., Guillari, A., da Valle, P., Gargiulo, G., Botti, S., Artioli, G., Comentale, G., Salvatore, F., Palma, G., & Baratta, S. (2018). The lived experience of parents whose children discharged to home after cardiac surgery for congenital heart disease. *Acta Biomedica, 89*(4-s), 71-77.

Sira, N., Desai, P. P., Sullivan, K. J., & Hannon, D. W. (2014). Coping strategies in mothers of children with heart defects: A closer look into spirituality and internet utilization. *Journal of Social Service Research, 40*, 606-622.

Skevington, S. M., Lotfy, M., & O'Connell, K. A. (2004). The World Health Organization's WHOQOL-BREF quality of life assessment: psychometric properties and results of the international field trial. A report from the WHOQOL group. *Quality of life Research, 13*, 299-310.

Solomon, Z., Mikulincer, M., & Avitzur, E. (1988). Coping, locus of control, social support, and combat-related posttraumatic stress disorder: a prospective study. *Journal of Personality and Social Psychology, 55*, 279-285.

Somasiri & Gunawardana. (1995). Assessment of quality of life using Sinhala translation of WHOQOL-100.

Spanier, G. B. (1976). Measuring Dyadic Adjustment: New Scales for Assessing the Quality of Marriage and Similar Dyads. *Journal of Marriage and Family, 38*, 15-28.

Spielberger, C. D., Gorsuch, R. L., Lushene, R., Vagg, P. R. (1983). *Manual for the State-Trait Anxiety Inventory*. Consulting Psychologists Press.

Spijkerboer, A., Helbing, W., Bogers, A., Van Domburg, R., Verhulst, F., & Utens, E. (2007). Long-term psychological distress, and styles of coping, in parents of children and adolescents who underwent invasive treatment for congenital cardiac disease. *Cardiology in the Young, 17*, 638-645.

Spitzer, R. L., Kroenke, K., Williams, J. B. W., & Löwe, B. (2006). A brief measure for assessing generalized anxiety disorder: the GAD-7. *Archives of Internal Medicine, 166*, 1092-1097.

Stein, R. E. K., & Jessop, J. D. (2003). The Impact on Family Scale Revisited: Further psychometric data. *Journal of Developmental & Behavioral Pediatrics, 24*, 9-16.

Stein, R. E. K., & Riessman, K. C. (1980). The Development of an Impact-on-Family Scale: Preliminary findings. *Medical Care, 18*, 465-472.

Stein, R. E. K., & Jessop, J.D. (1985). *Tables documenting the psychometric properties of a measure of the impact of chronic illness on the family*. Albert Einstein College of Medicine.

Stoffel, G., Spirig, R., Stiasny, B., Bernet, V., Dave, H., & Knirsch, W. (2017). Psychosocial impact on families with an infant with a hypoplastic left heart syndrome during and after the interstage monitoring period – a prospective mixed-method study. *Journal of Clinical Nursing, 26*(21-22), 3363-3370.

Streisand, R., Braniecki, S., Tercyak, K. P., & Kazak, A. E. (2001). Childhood illness-related parenting stress: The Pediatric Inventory for Parents. *Journal of Pediatric Psychology, 26*, 155-162.

Svavarsdottir, E. K., & McCubbin, M. (1996). Parenthood transition for parents of an infant diagnosed with a congenital heart condition. *Journal of Pediatric Nursing, 11*(4), 207-216.

Tallon, M. M., Kendall, G. E., & Snider, P. D. (2015). Development of a measure for maternal confidence in knowledge and understanding and examination of psychosocial influences at the time of a child's heart surgery. *Journal for Specialists in Pediatric Nursing, 20*, 36-48.

Taylor, J. A. (1951). The relationship of anxiety to the conditioned eyelid response. *Journal of Experimental Psychology, 41*(2), 81-92.

Taylor, J. A. (1953). A personality scale of manifest anxiety. *The Journal of Abnormal and Social Psychology, 48*(2), 285-290.

Terwee, C. B., Bot, S. D., de Boer, M. R., van der Windt, D. A., Knol, D. L., Dekker, J., Bouter, L. M., & de Vet, H. C. (2007). Quality criteria were proposed for measurement properties of health status questionnaires. *Journal of Clinical Epidemiology, 60*, 34-42.

Tielemans, N. S., Visser-Meily, J. M., Schepers, V. P., Post, M. W., & van Heugten, C. M. (2014). Proactive coping post stroke: Psychometric properties of the Utrecht Proactive Coping Competence Scale. *Archives of Physical Medicine and Rehabilitation, 95*, 670-675.

Torowicz, D., Irving, S. Y., Hanlon, A. L., Sumpter, D. F., & Medoff-Cooper, B. (2010). Infant temperament and parental stress in 3-month-old infants after surgery for complex congenital heart disease. *Journal of Developmental & Behavioral Pediatrics, 31*, 202-208.

Uhm, J. Y., & Kim, H. S. (2019). Impact of the mother–nurse partnership programme on mother and infant outcomes in paediatric cardiac intensive care unit. *Intensive and Critical Care Nursing, 50*, 79-87.

Ulusoy, M., Sahin, N. H., & Erkmen, H. (1998). Turkish version of the Beck Anxiety Inventory: Psychometric properties. *Journal of Cognitive Psychotherapy, 12*, 163-172.

Utens, E. M., Versluis-Den Bieman, H. J., Verhulst, F. C., Witsenburg, M., Bogers, A. J., & Hess, J. (2000). Psychological distress and styles of coping in parents of children awaiting elective cardiac surgery. *Cardiology in the Young, 10*, 239-244.

Uzark, K., & Jones, K. (2003). Parenting stress and children with heart disease. *Journal of Pediatric Health Care, 17*, 163-168.

Üzger, A., Başpinar, O., Bülbül, F., Yavuz, S., & Kilinç, M. (2015). Evaluation of depression and anxiety in parents of children undergoing cardiac catheterization. *Turk Kardiyol Dern Ars, 43*, 536-541.

van der Mheen, M., Meentken, M. G., van Beynum, I. M., van der Ende, J., van Galen, E., Zirar, A., Aendekerk, E.W.C., van den Adel, T.P.L., Bogers, A.J.J.C., McCusker, C.G., Hillegers, M.H.J., Helbing, W.A.., & Utens, E. (2019). CHIP-Family intervention to improve the psychosocial well-being of young children with congenital heart disease and their families: Results of a randomised controlled trial. *Cardiology in the Young, 29*, 1172-1182.

van Rijsoort, S., Emmelkamp, P., & Vervaeke, G. (1999). The Penn state worry questionnaire and the worry domains questionnaire: Structure, reliability and validity. *Clinical Psychology & Psychotherapy: An International Journal of Theory & Practice, 6*, 297-307.

Varni, J. W., Burwinkle, T. M., Katz, E. R., Meeske, K., & Dickinson, P. (2002). The PedsQL™ in pediatric cancer: reliability and validity of the pediatric quality of life inventory™ generic core scales, multidimensional fatigue scale, and cancer module. *Cancer, 94*, 2090-2106.

Varni, J. W., Seid, M., & Rode, C. (1999). The PedsQL ™: Measurement model for the pediatric quality of life inventory. *Medical Care, 37*, 126-139.

Varni, J. W., Sherman, S. A., Burwinkle, T. M., Dickinson, P. E., & Dixon, P. (2004). The PedsQL™ Family Impact Module: Preliminary reliability and validity. *Health and Quality of Life Outcomes, 2*(1), 55.

Veit, C. T., & Ware, J. E. (1983). The structure of psychological distress and well-being in general populations. *Journal of Consulting and Clinical Psychology, 51*, 730-742.

Vinokur, A., & Caplan, R. D. (1987). Attitudes and social support: Determinants of job-seeking behavior and well-being among the unemployed. *Journal of Applied Social Psychology, 17*, 1007-1024.

Visconti, J. K., Saudino, J. K., Rappaport, A. L., Newburger, W. J., & Bellinger, C. D. (2002). Influence of parental stress and social support on the behavioral adjustment of children with Transposition of the Great Arteries. *Journal of Developmental & Behavioral Pediatrics, 23*, 314-321.

Wang, J., Kelly, B. C., Liu, T., Zhang, G., & Hao, W. (2013). Factorial structure of the Brief Symptom Inventory (BSI)-18 among Chinese drug users. *Drug and Alcohol Dependence, 133*, 368-375.

Wang, L., Yang, X. S., & Hou, Z. (2006). Application and evaluation of Chinese version of zarit caregiver burden interview. *Chinese Journal of Public Health, 22*(8), 970-972.

Ware, J. E., Kosinski, M., & Gandek, B. (2000). *SF-36 health survey: manual & interpretation guide* [2nd ed.]

Warnakulasooriya, P. H., & Kasturiaratchi, K. (2020). Quality of life of caregivers with children having congenital heart disease awaiting cardiac surgery at the Lady Ridgeway Hospital for Children, Colombo, Sri Lanka. *Sri Lanka Journal of Child Health, 49*(1), 17-22.

Weathers, F. W., Litz, B. T., Herman, D.S. Huska, J.A., Keane, T.M. (1993). *The PTSD Checklist (PCL): Reliability, validity, and diagnostic utility* 9th Annual Conference of the ISTSS, San Antonio, TX.

Weiss, D. S. (2007). The Impact of Event Scale: Revised. In J. P. S.-K. T. Wilson, Catherine C (Ed.), *Cross-cultural Assessment of Psychological Trauma and PTSD*. Springer.

Weiss, D. S. M., & Charles R. (1997). The Impact of Event Scale - Revised. In J. P. K. Wilson, Terence M (Ed.), *Assessing Psychological Trauma and PTSD*. Guilford Publications.

Werner, O., El Louali, F., Fouilloux, V., Amedro, P., & Ovaert, C. (2019). Parental anxiety before invasive cardiac procedure in children with congenital heart disease: Contributing factors and consequences. *Congenital Heart Disease, 14*, 778-784.

Whoqol Group. (1998). Development of the World Health Organization WHOQOL-BREF quality of life assessment. *Psychological Medicine, 28*, 551-558.

Williams, A. (1990). EuroQol - a new facility for the measurement of health-related quality of life. *Health Policy, 16*, 199-208.

Yildiz, A., Celebioglu, A., & Olgun, H. (2009). Distress levels in Turkish parents of children with congenital heart disease. *Australian Journal of Advanced Nursing, 26*(3), 39-46.

Zhang, Q. L., Xu, N., Huang, S. T., Cao, H., & Chen, Q. (2021). WeChat-assisted pre-operative health education improves the quality of life of parents of children with ventricular septal defects: A prospective randomised controlled study. *Journal of Paediatrics and Child Health, 57*, 664-669.

Zhang, Q. L., Xu, N., Ultimos, P., Huang, S. T., Chen, Q., & Cao, H. (2020). WeChat-assisted preoperative health education reduces burden of care on parents of children with simple congenital heart disease: A prospective randomized controlled study. *Brazilian journal of Cardiovascular Surgery, 36,* 663-669.

Zigmond, A. S., & Snaith, R. P. (1983). The Hospital Anxiety and Depression Scale. *Acta Psychiatrica Scandinavica, 67*, 361-370.

Zung, W. W. (1971). A rating instrument for anxiety disorders. *Psychosomatics, 12*, 371-379.

Referenced Websites

Symptom Checklist-90-Revised (SCL-90-R): https://www.pearsonassessments.com/store/usassessments/en/Store/Professional-Assessments/Personality-%26-Biopsychosocial/Symptom-Checklist-90-Revised/p/100000645.html

Brief Symptom Inventory (BSI): https://www.pearsonassessments.com/store/usassessments/en/Store/Professional-Assessments/Personality-%26-Biopsychosocial/Brief-Symptom-Inventory/p/100000450.html

Brief Symptom Inventory 18 (BSI-18): https://www.pearsonassessments.com/store/usassessments/en/Store/Professional-Assessments/Personality-%26-Biopsychosocial/Brief-Symptom-Inventory-18/p/100000638.html

General Health Questionnaire (GHQ-60, GHQ-30, GHQ-28, GHQ-12): https://eprovide.mapi-trust.org/instruments/general-health-questionnaire; https://support.gl-education.com/knowledge-base/assessments/general-health-questionnaire-support/about-the-general-health-questionnaire/faqs/

Perceived Stress Scale (PSS): https://www.cmu.edu/dietrich/psychology/stress-immunity-disease-lab/scales/index.html

Index of Clinical Stress: https://shop.walmyr.com/shop/ICS

Depression, Anxiety and Stress Scale (DSS) & Depression, Anxiety and Stress Scale-21 (DASS 21): http://www2.psy.unsw.edu.au/dass/

Hospital Anxiety and Depression Scale (HADS): https://www.gl-assessment.co.uk/products/hospital-anxiety-and-depression-scale-hads/

Beck Depression Inventory-Second Edition (BDI-2): https://www.pearsonassessments.com/store/usassessments/en/Store/Professional-Assessments/Personality-%26-Biopsychosocial/Beck-Depression-Inventory-II/p/100000159.html

Patient Health Questionnaire (PHQ): https://www.apa.org/pi/about/publications/caregivers/practice-settings/assessment/tools/patient-health

Edinburgh Postnatal Depression Scale (EPDS): https://www.cope.org.au/health-professionals/health-professionals-3/calculating-score-epds/; https://www.mhcs.health.nsw.gov.au/publications/epds?collectionfilter=1

State-Trait Anxiety Inventory (STAI): https://www.mindgarden.com/145-state-trait-anxiety-inventory-for-adults

Beck Anxiety Inventory (BAI): https://www.pearsonassessments.com/store/usassessments/en/Store/Professional-Assessments/Personality-%26-Biopsychosocial/Beck-Anxiety-Inventory/p/100000251.html?tab=overview

Generalized Anxiety Disorder-7 (GAD-7): https://eprovide.mapi-trust.org/instruments/generalized-anxiety-disorder-7

Impact of Event Scale-Revised (IES-R): https://eprovide.mapi-trust.org/instruments/impact-of-event-scale-revised

PTSD Checklist – Civilian Version (PCL-C): https://istss.org/clinical-resources/assessing-trauma/ptsd-checklist-dsm-5/ptsd-checklist-dsm-iv

Posttraumatic Diagnostic Scale (PDS): https://eprovide.mapi-trust.org/instruments/posttraumatic-diagnostic-scale-r

Acute Stress Disorder Scale (ASDS): https://www.healthcaretoolbox.org/sites/default/files/images/pdf/ASDS.pdf

Maslach's Burnout Inventory (MBI): https://www.mindgarden.com/117-maslach-burnout-inventory-mbi

Ottawa Mood Scale: https://www.drcheng.ca/resources/Articles/mood_scales-facesforallages.pdf

Beck Hopelessness Scale: https://www.pearsonclinical.com.au/products/view/42

Parenting Stress Index Fourth Edition (PSI-4): https://www.parinc.com/Products/Pkey/333

Parenting Stress Index-Short Form Fourth Edition (PSI-4-SF): https://www.parinc.com/products/pkey/335

Zarit Caregiver Burden: https://eprovide.mapi-trust.org/instruments/zarit-burden-interview

36-Item Short-Form Health Survey (SF-36): https://www.rand.org/health-care/surveys_tools/mos/36-item-short-form.html

12-item Short-Form Health Survey (SF-12): https://www.qualitymetric.com/health-surveys/the-sf-12v2-health-survey/

Perceived Quality of Life Scale (PQOL): http://depts.washington.edu/seaqol/PQOL

PedsQL Family Impact Module: https://eprovide.mapi-trust.org/instruments/pediatric-quality-of-life-inventory-family-impact-module

World Health Organization Quality of Life (WHOQOL-100): https://www.who.int/tools/whoqol/whoqol-100

World Health Organization Quality of Life BREF (WHOQOL-BREF): https://www.who.int/tools/whoqol/whoqol-bref

Family Member Well-being Index (FMWB): https://www.mccubbinresilience.org/measures.html

Quality of Life Linear Analogue Scale: https://euroqol.org/eq-5d-instruments/

Psychosocial Assessment Tool (PAT2.0): https://www.psychosocialassessmenttool.org/

Family Assessment Device (FAD): https://www.nctsn.org/measures/family-assessment-device

Family Environment Scale (FES): https://www.mindgarden.com/96-family-environment-scale

Impact on Family Scale (IOFS): https://www.apa.org/pi/about/publications/caregivers/practice-settings/assessment/tools/family-impact

Family Inventory of Life Events (FILE): https://www.mccubbinresilience.org/measures.html

Family Hardiness Index (FHI): https://www.mccubbinresilience.org/measures.html

Coping Inventory for Stressful Situations (CISS): https://paa.com.au/product/ciss/

Coping Health Inventory for Parents (CHIP): https://www.mccubbinresilience.org/measures.html; https://www.apa.org/pi/about/publications/caregivers/practice-settings/assessment/tools/coping-health

COPE Inventory: https://local.psy.miami.edu/faculty/ccarver/sclCOPEF.phtml

Brief COPE: https://local.psy.miami.edu/faculty/ccarver/sclBrCOPE.phtml

Ways of Coping Questionnaire (WCQ): https://www.mindgarden.com/158-ways-of-coping-questionnaire

Coping Scale for Adults Second Edition: https://shop.acer.org/coping-scale-for-adults-second-edition-csa-2.html

Response to Stress Questionnaire (RSQ-CHD): https://my.vanderbilt.edu/stressandcoping/rsq/

Hassles and Uplifts Scale (HSUP): https://www.mindgarden.com/108-hassles-uplifts#horizontalTab4

Parent Stressor Scale: Infant Hospitalization (PSS-IH): https://eprovide.mapi-trust.org/instruments/parental-stressor-scale-infant-hospitalization

Parent Stressor Scale: NICU (PSS-NICU): https://eprovide.mapi-trust.org/instruments/parental-stressor-scale-neonatal-intensive-care-unit

Dyadic Adjustment Scale (DAS): https://paa.com.au/product/das/

Evaluating and Nurturing Relationship Issues Communication and Happiness Scale (ENRICH): https://www.prepare-enrich.com/
